# Proximity biotinylation at the host-*Shigella* interface reveals UFMylation as an antibacterial pathway

**DOI:** 10.1038/s41467-026-77860-y

**Published:** 2026-09-17

**Authors:** Ana T. López-Jiménez, Fabien Théry, Kathryn Wright, Hannah Painter, Shelby T. Hoffmeister, Lucas Jarche, Jeremy Benjamin, Gerbrand J. van der Heden van Noort, Dominik Brokatzky, Margarida C. Gomes, Sydney L. Miles, Damián Lobato-Márquez, Eva-Maria Frickel, John Rohde, Jonathan N. Pruneda, Francis Impens, Serge Mostowy

**Affiliations:** 1 Department of Infection Biology, London School of Hygiene and Tropical Medicine, London, UK; 2 Department of Microbiology and Molecular Medicine, University of Geneva, Geneva, Switzerland; 3 VIB-UGent Center for Medical Biotechnology, VIB, Ghent, Belgium; 4 Department of Biomolecular Medicine, Ghent University, Ghent, Belgium; 5 Department of Molecular Microbiology and Immunology, Oregon Health & Science University, Portland, Oregon USA; 6 Department of Microbiology and Immunology, Dalhousie University, Halifax, Nova Scotia Canada; 7 Department of Cell and Chemical Biology, Leiden University Medical Centre, Leiden, The Netherlands

**Keywords:** Cellular microbiology, Bacteria, Bacterial infection

## Abstract

Host cells contest invasion by intracellular bacterial pathogens with multiple strategies that recognise and/or damage the bacterial surface. To identify host defence factors targeted to intracellular bacteria, we developed a proximity biotinylation approach coupled to quantitative mass spectrometry that maps the host-bacterial interface during infection. Using this method, we discovered that intracellular *Shigella* and *Salmonella* become targeted by UFM1-protein ligase 1 (UFL1), an E3 ligase that catalyses the covalent attachment of Ubiquitin-fold modifier 1 (UFM1) to target substrates in a process called UFMylation. *Shigella* antagonises UFMylation in a dual manner: first, using its lipopolysaccharide to shield from UFL1 recruitment; second, preventing UFM1 decoration by the bacterial effector IpaH9.8. Absence of UFMylation leads to an increase of bacterial burden in human cells and zebrafish larvae. Contrary to ubiquitylation, the protective role of UFMylation is independent of autophagy. Altogether, our proximity mapping of the host-bacterial interface identifies UFMylation as an ancient antibacterial pathway and holds great promise to reveal other cell-autonomous immunity mechanisms.

## Abstract (web summary)

Here, López-Jiménez et al. develop a proximity biotinylation approach to map the host-bacterial interface and show that intracellular *Shigella* and *Salmonella* are targeted by UFMylation, a ubiquitin-like system.

## Introduction

*Shigella flexneri* is a human-adapted enteroinvasive pathogen that causes over 200 million episodes of bacillary dysentery annually worldwide^1^. Its virulence relies on a Type 3 Secretion System (T3SS) that injects around 30 bacterial effectors into host cells to modulate key aspects of the infection process^2,3^. Once in the gastrointestinal tract, *S. flexneri* invades colonic epithelial cells and escapes from the phagosome to the cytosol within 10 min^4^. *S. flexneri* has evolved to thrive in the host cytosol, where it replicates and hijacks the actin cytoskeleton to form distinctive actin tails that enable intracellular motility and cell-to-cell spread^5,6^.

Cells protect their nutrient-rich cytosol from bacterial intruders with a plethora of highly conserved mechanisms collectively known as cell-autonomous immunity^7^. These include the prevention of actin tail motility by the entrapment of *S. flexneri* in septin cage-like structures^8,9^ and guanylate-binding protein (GBP) coats^10,11^. GBPs have also been shown to bind and disrupt the bacterial lipopolysaccharide (LPS)^12^, which exposes bacterial membranes to permeabilisation by Apolipoprotein L3^13^. In addition, bacterial LPS becomes ubiquitylated by the E3 ubiquitin ligase ring finger protein 213 (RNF213), which is required for the subsequent extension of the ubiquitin bacterial coat by other host E3 ligases (LRSAM1, Parkin, LUBAC and SMURF1)^14^. Although bacterial ubiquitylation by E3 ligases typically targets intracellular bacteria such as *Salmonella* to degradation by autophagy (xenophagy) via specific cargo receptors, *S. flexneri* avoids autophagosome recruitment by the effectors IcsB, VirA, and likely other mechanisms yet to be understood^15–20^.

In addition to canonical ubiquitylation, eukaryotic cells possess multiple ubiquitin-like (UBL) systems. These are post-translational modification machineries where small peptides with low sequence homology but high structural similarity to ubiquitin (including the β-grasp fold or ubiquitin fold) become conjugated to target substrates by the sequential action of an E1 activating, an E2 conjugating and an E3 ligating enzyme^21^, in a similar process to ubiquitylation. Although several UBL systems (FAT10ylation^22^, ISGylation^23^, NEDDylation^24,25^, SUMOylation^26,27^) are protective against various bacterial infections, a direct antibacterial role at the bacterial surface analogous to ubiquitylation remains less well established.

Here, we develop a versatile nanobody-based proximity biotinylation approach to map host proteins enriched in the vicinity of cytosolic *S. flexneri* during infection using quantitative mass spectrometry. Using this method, we discover that *S. flexneri* and *Salmonella enterica* subsp. *enterica* srv. Typhimurium becomes targeted by UFMylation, a highly conserved UBL system^28^ that has recently emerged as a major regulator of cell homoeostasis. This is particularly well studied at the endoplasmic reticulum (ER), where UFMylation is involved in stress tolerance through ribosome-associated quality control^29^ and selective ER autophagy (ER-phagy)^30^. In addition, UFMylation has been shown to be involved in DNA damage responses^31^, telomere length maintenance^32^, lipid droplet biogenesis^33^, autophagy regulation^34^, and immune signalling^35,36^. Here, we show that UFMylation also has a cell-autonomous immunity role against intracellular bacteria. We show that *S. flexneri* counteracts UFMylation via two distinct mechanisms: its LPS hampers the recruitment of the E3 ligase UFL1, and the secreted effector IpaH9.8 antagonises decoration of the UBL molecule UFM1 on the surface of intracellular bacteria. While UFMylated bacteria are not targeted for degradation by autophagy, the lack of UFMylation leads to increased bacterial burden in human epithelial cells and zebrafish larvae. Together, we identify UFMylation as a highly conserved cell-autonomous immunity pathway against bacterial infection.

## Results

### Proximity biotinylation approach at the host-bacterial interface identifies UFL1 as targeted to intracellular bacteria

**Fig. 1:**
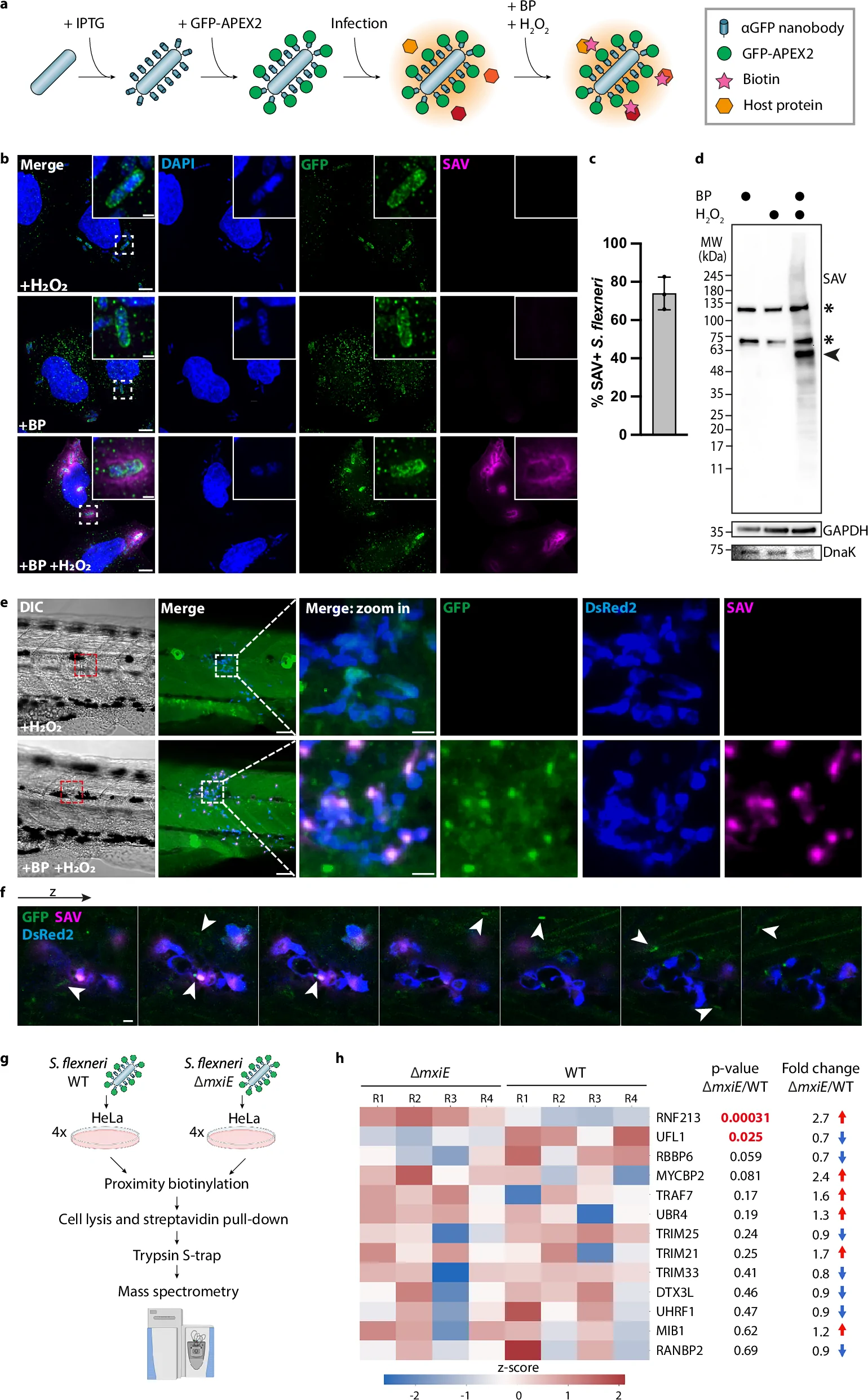
Proximity biotinylation approach. **a** A strategy for in vitro functionalisation of *Shigella* for proximity mapping. **b** Airyscan confocal images showing biotinylation in the vicinity of functionalised *Shigella* during HeLa cell infection, in the presence or absence of BP and H_2_O_2_ at 1.5 h.p.i. Representative images from 2 independent experiments. GFP-APEX2 was detected using an anti-GFP antibody. SAV stands for Alexa Fluor 647-conjugated streptavidin. Scale bar, 5 µm; inset, 1 µm. **c** Quantification of functionalised *Shigella* presenting a streptavidin halo in the presence of BP and H_2_O_2_, as shown in (**b**) (*n* = 560 bacteria, 3 independent experiments). Results are represented as mean ± SD. **d** Western blot shows a smear of biotinylated proteins in HeLa cells infected with functionalised *Shigella* upon addition of BP and H_2_O_2,_ detected using HRP-conjugated streptavidin. Asterisk shows unspecific bands, arrow shows band likely corresponding to GFP-APEX2. GAPDH is used as HeLa cell loading control, and DnaK shows a similar level of infection across samples. Representative blot from 3 independent experiments. **e** Proximity biotinylation occurs in zebrafish larvae at 45 min post infection of functionalised *Shigella* at the tail musculature. Representative images of *n* = 5 larvae (BP and H_2_O_2_) and *n* = 4 larvae (H_2_O_2_ alone). Transgenic zebrafish *Tg(lyzC:DsRed2)*^*nz50*^ used to visualise fluorescent neutrophils; GFP-APEX2 detected using anti-GFP antibody. Scale bar, 50 µm; inset, 10 µm. **f** Representative high magnification image of zebrafish larvae (*n* = 5) infected with functionalised *Shigella* and treated with BP and H_2_O_2_, as shown in (**e**). Representative z-planes from the same z-stack are shown; arrows indicate GFP-positive bacteria. Scale bar, 5 µm. **g** An experimental workflow to identify the proximity interactome of *Shigella* WT and Δ*mxiE* mutant using mass spectrometry in HeLa cells. Created in BioRender. Lopez, A. (2026) https://BioRender.com/qa8tfz4. **h** Heatmap of E3 ligases found in proximity interactome of *Shigella* during infection of HeLa cells at 1.5 h.p.i., inversely ordered by *P* value from 4 biological replicates. Source data provided as Source Data file.

Recognition and restriction of intracellular pathogens by cell-autonomous immunity provides an immediate and localised defence against infection before the organism can mount an immune response. Importantly, many cell-autonomous immunity mechanisms that host cells deploy against bacterial intruders are delivered to the bacterial surface. We exploited this feature to develop a proximity biotinylation approach that specifically maps host proteins at the interface between the host and the bacterial surface during *S. flexneri* infection (i.e., the bacterial “proximity interactome”). For this, we engineered *S. flexneri* to display anti-GFP nanobodies on its surface^37^, which enabled their functionalisation in vitro with GFP-fused recombinant proteins such as APEX2, an engineered ascorbate peroxidase widely used for proximity biotinylation campaigns^38^ (Fig. 1a and Supplementary Fig. 1a, b). In a second step, bacteria functionalised with GFP-APEX2 can be used in infection assays for the biotinylation of host proteins in close proximity to bacteria and subsequent identification by mass spectrometry. In vitro, we observed specific biotinylation of the *S. flexneri* surface when the anti-GFP nanobody was expressed, and exogenous biotin-phenol and H_2_O_2_ were added (Supplementary Fig. 1c–f). We showed that GFP-APEX2 functionalised bacteria were able to infect HeLa cells and form actin tails and septin cages, which are considered hallmarks of the *S. flexneri* cytosolic lifestyle (Supplementary Fig. 2a, b). In addition, we detected de novo DNA synthesis in functionalised bacteria during HeLa cell infection using the clickable thymidine analogue EdU (Click-iT EdU, Supplementary Fig. 2c, d), confirming they were metabolically active; we also verified that biotinylation reagents did not affect the fitness of bacteria in broth (Supplementary Fig. 2e).

In HeLa, biotinylated proteins appeared as a halo around 73.9 ± 8.5% of bacteria during infection by fluorescence microscopy (Fig. 1b, c) and as a smear by western blot (Fig. 1d). Biotinylation of the *S. flexneri* microenvironment also occurred in vivo during infection of zebrafish larvae, highlighting the versatility of the method beyond tissue culture cells (Fig. 1e, f and Supplementary Movie 1).

Using this approach, we identified the proximity interactome of *S. flexneri* during infection in HeLa cells by label-free quantitative mass spectrometry. We compared the proximity interactome generated by wildtype (WT) bacteria and bacteria lacking the transcriptional regulator MxiE, which controls expression of late T3SS effectors, including important factors that antagonise cell-autonomous immunity (Fig. 1g and Supplementary Fig. 2f–i). To this end, HeLa cells were infected with GFP-APEX2 functionalised WT or Δ*mxiE S. flexneri* and biotinylated proteins were pulled-down for liquid chromatography-tandem mass spectrometry (LC-MS/MS analysis, Fig. 1g). We identified and quantified a total of 1298 proteins of which 9 were *S. flexneri* proteins and 1289 were human host proteins (Supplementary Data File 1 and Supplementary Fig. 3). GO term enrichment analysis of the host proteins identified multiple pathways related to host-pathogen interactions relevant for *S. flexneri* infection, including actin filament-based transport, outer-mitochondrial membrane organisation or stress granule assembly (Supplementary Fig. 3c). These data included proteins well known to localise to the *S. flexneri* surface, such as Septin 7^8^ or the Arp2/3 component Actin-related protein 2^6^ (Supplementary Data File 2). In addition, proteomic screening revealed that multiple members of the ubiquitylation machinery are recruited to the bacterial surface during infection, including 13 E3 ligases (Fig. 1h and Supplementary Fig. 3d). One of the most enriched hits in the proximity interactome of the *S. flexneri* Δ*mxiE* was RNF213, which was discovered to ubiquitylate the LPS of cytosolic *S*. Typhimurium^14^ and *S. flexneri*^18–20^. It has been recently shown that the MxiE*-*dependent effector IpaH1.4 prevents RNF213 recruitment to *S. flexneri*, explaining why RNF213 is enriched to Δ*mxiE* compared to WT bacteria in our proximity data. Considering the pivotal role of ubiquitin E3 ligases in host defence^39^, we decided to focus on the E3 UFM1-protein Ligase 1 (UFL1, Fig. 1h), an enzyme that catalyses the covalent attachment of Ubiquitin-like modifier 1 (UFM1) to target proteins in a process called UFMylation, similar to ubiquitylation. UFL1 had the second lowest *P* value among the E3 ligases identified (Fig. 1h). Together, we show that our proximity biotinylation approach is effective to identify host factors in close proximity to *S. flexneri* during infection, including UFL1 and other E3 ligases.

### *S. flexneri* LPS shields cytosolic bacteria from recognition by the UFMylation ligase UFL1

**Fig. 2:**
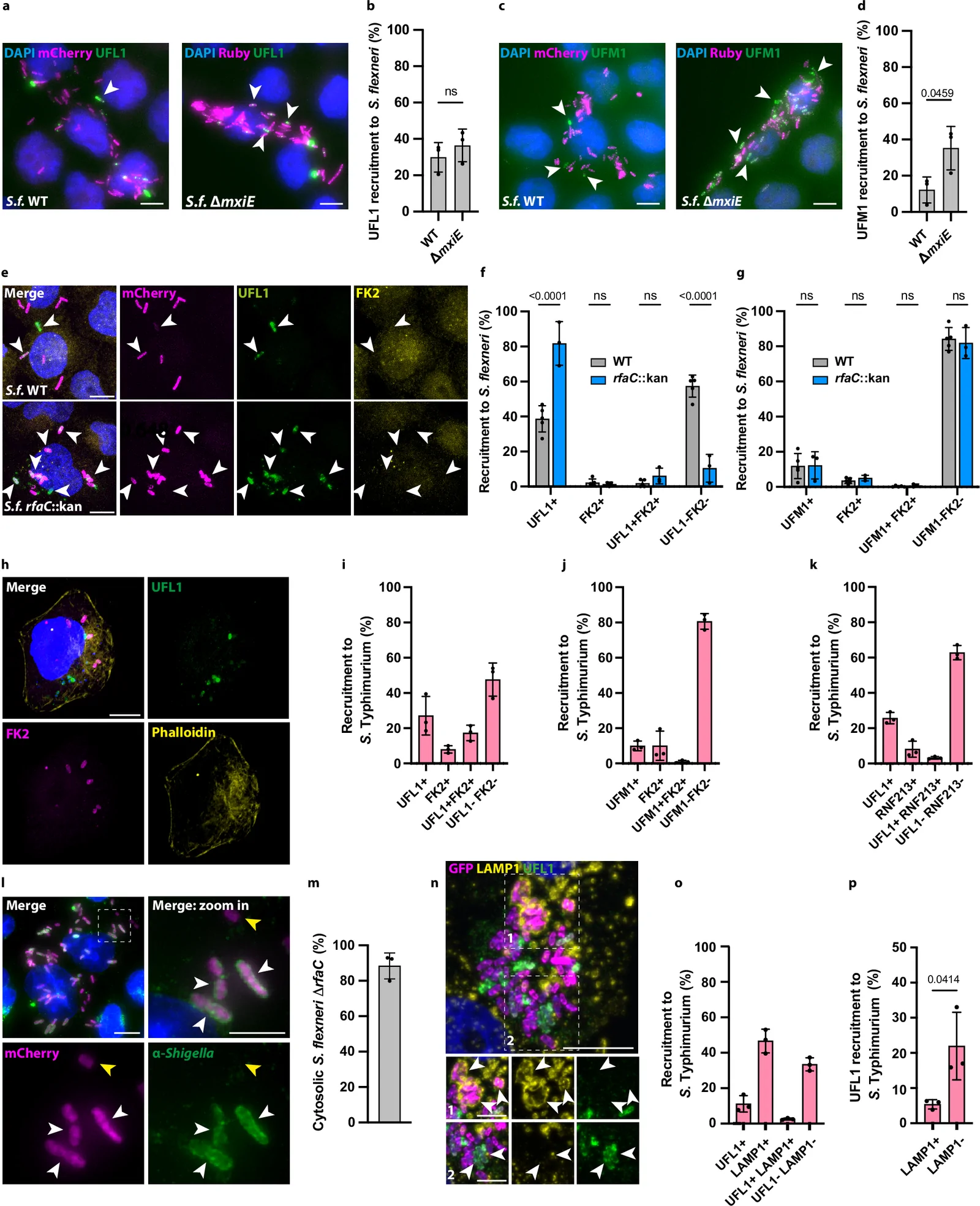
UFMylation pathway members recruited to cytosolic *Shigella* and *Salmonella.* **a**,** c** Representative widefield images of HeLa cells infected with mCherry *Shigella* WT and Ruby *Shigella* Δ*mxiE* at 3 h.p.i. Arrows show UFL1 and UFM1 recruitment to bacteria, respectively. **b**,** d** Percentage of mCherry *Shigella* WT (*n* = 1011) and Ruby *Shigella* Δ*mxiE* (*n* = 776) colocalising with UFL1 (*n* = 1011 for WT; *n* = 776 for Δ*mxiE*) and UFM1 (*n* = 1,620 for WT; *n* = 1044 for Δ*mxiE*), 3 independent experiments. **e** Representative confocal images of HeLa cells infected with mCherry *Shigella* WT and *rfaC*::kan at 3 h.p.i. Arrows show UFL1 recruitment to bacteria. **f** Percentage of mCherry *Shigella* WT (*N* = 5 independent experiments, *n* = 1311) and *rfaC*::kan (*N* = 3 independent experiments, *n* = 630) colocalising with UFL1 and ubiquitin (FK2). **g** Percentage of mCherry *Shigella* WT (*N* = 5 independent experiments, *n* = 882) and *rfaC*::kan (*N* = 3 independent experiments, *n* = 643) colocalising with UFM1 and ubiquitin (FK2). **h** Representative confocal image of HeLa cells infected with *Salmonella* and immunostained for UFL1 and ubiquitin (FK2) at 3 h.p.i. **i–k** Percentage of *Salmonella* (*n* = 740) colocalising with UFL1 and ubiquitin (FK2) (*n* = 740), UFM1 and FK2 (*n* = 823), and UFL1 and GFP-RNF213 (*n* = 735), 3 independent experiments. **l** Representative widefield image of digitonin-permeabilised cells infected with mCherry *Shigella rfaC*::kan at 3 h.p.i. White and yellow arrows show positive and negative bacteria for the anti-*Shigella* antibody, respectively. **m** Percentage of mCherry *Shigella rfaC*::kan (*n* = 1063) positive for anti-*Shigella* antibody, 3 independent experiments. **n** Representative widefield image of HeLa cells infected with GFP *Salmonella* at 3 h.p.i., immunostained for LAMP1. Arrows in insets 1 and 2 show vacuolar and cytosolic bacteria, respectively. **o** Percentage of GFP *Salmonella* (*n* = 743) colocalising with UFL1 and LAMP1, 3 independent experiments. **p** Percentage of UFL1 positive *Salmonella* (*n* = 743) found in *Salmonella*-containing vacuole (LAMP1-positive) or cytosol (LAMP1-negative). **b**, **d**, **p** Two-sided Student’s t-test. **f**, **g** Two-way ANOVA and Tukey’s test, two-sided. Results represented as mean ± SD. n.s: non-significant. Scale bars, 10 µm; insets, 5 µm. Source data are provided as Source Data file.

Microscopy experiments validated the localisation of UFL1 and UFM1 to the surface of *S. flexneri* during infection in HeLa cells, using both HA-tagged exogenous expression and antibodies against the endogenous proteins (Fig. 2a–d and Supplementary Fig. 3e). Microscopy-based quantification did not reveal significant differences in the proportion of UFL1 decorated bacteria between *S. flexneri* WT and Δ*mxiE* (Fig. 2a, b), but we observed that UFM1 recruitment to *S. flexneri* Δ*mxiE* was increased compared to WT (WT 12.1 ± 7.2%, Δ*mxiE* 35.3 ± 12.0% bacteria, Fig. 2c, d), suggesting MxiE-dependent antagonism downstream of UFL1 recruitment to the bacteria.

Considering that LPS is the bacterial substrate for ubiquitylation by the E3 ligase RNF213^14^, we tested the impact of UFL1 and UFM1 recruitment to an *S. flexneri* mutant *rfaC*::kan, which lacks the O-antigen and the LPS outer core^40^. We observed a significant increase in UFL1 recruitment in *S. flexneri rfaC*::kan (2.1 ± 0.2-fold), which was restored to WT levels upon complementation with *rfaC* (Fig. 2e, f and Supplementary Fig. 4c, e), suggesting that LPS protects intracellular bacteria from UFL1 binding. However, this increase in UFL1 binding did not correlate with an increase in UFM1 decoration of *S. flexneri* (WT 12.2 ± 5.0%, *rfaC*::kan 13.1 ± 8.5% bacteria, Fig. 2g and Supplementary Fig. 4f), suggesting that additional bacterial factors may prevent UFM1 recruitment to bacteria, in agreement with our Δ*mxiE* results (Fig. 2c, d**)**. In both *S. flexneri* WT and *rfaC*::kan, we observed similar and low values of ubiquitylation (3.9 ± 2.9 and 6.6 ± 3.6 bacteria, respectively), and there was little overlap between UFM1 and ubiquitin-positive bacteria (Fig. 2g), consistent with IpaH1.4 promoting RNF213 degradation^18–20^.

We hypothesised that the UFMylation machinery may recognise other intracellular bacteria beyond *S. flexneri*. We tested *S*. Typhimurium, a bacterium that typically resides within vacuoles but occasionally escapes into the cytosol, where it hyperreplicates^41^. We observed that UFL1 and UFM1 were also recruited to the surface of *S*. Typhimurium at 3 h post infection (h.p.i., Fig. 2h-j), indicating that targeting of bacteria by the UFMylation machinery is not a phenotype exclusive to *S. flexneri*. Most bacteria recruiting UFL1 or UFM1 did so in the absence of GFP-RNF213 recruitment or ubiquitylation (Fig. 2h–k and Supplementary Fig. 4g). Despite some overlap between UFL1 and FK2 decoration (17.3 ± 4.4% of the bacteria), robust recruitment of UFL1 and FK2 rarely coincided (Fig. 2h). Together, these results suggest that UFMylation and ubiquitylation are functionally separate machineries.

*S. flexneri* is a professional intracellular pathogen, with the majority of bacteria escaping from a phagocytic vacuole to the cytosol within 10 min after internalisation^4,42^, suggesting that UFMylation primarily targets cytosolic bacteria. We assessed localisation of the *S. flexneri rfaC*::kan mutant, which shows increased UFL1 association, as well as *S*. Typhimurium, which resides in a vacuole but a fraction can subsequently escape to the cytosol^43^. To determine localisation of the *S. flexneri rfaC*::kan mutant, we performed a digitonin permeabilisation assay, where the plasma membrane becomes selectively permeabilised while intracellular membranes remain largely intact^44^. Using this approach, we labelled 88.4 ± 7.4% of bacteria with anti-*Shigella* antibody, showing that most bacteria were accessible to the antibody in the cytosol (Fig. 2l, m and Supplementary Fig. 4h). These findings are consistent with previous findings reporting that loss of O-Antigen does not impair cytosolic escape of *S. sonnei*^45^. In the case of *S*. Typhimurium, we used the *Salmonella*-containing vacuole marker LAMP1 to discriminate cytosolic from vacuolar bacteria. Using both LAMP1-specific antibodies and cells expressing fluorescent mScarlet-I tagged LAMP1, we observed that the vast majority of UFL1-positive *S*. Typhimurium were not associated with LAMP1 (Fig. 2n–p and Supplementary Fig. 4i, j). In addition, UFL1 recruitment was observed in cells containing large numbers of *S*. Typhimurium (Supplementary Fig. 4k), characteristic of its hyperreplicative cytosolic stage. Finally, we infected HeLa cells with bacteria harbouring the reporter SINA1.7^46^, in which expression of the far-red fluorescent protein smURFP is driven by the P_*uhpT*_ promoter, activated upon exposure to cytosolic gluose-6-phosphate. We observed that UFL1 was recruited to *S*. Typhimurium expressing high levels of smURFP (Supplementary Fig. 4l), showing targeting of cytosolic bacteria. Together, we conclude that UFMylation machinery targets cytosolic bacteria and that LPS limits UFL1 recognition.

### UFM1 decoration of intracellular *S. flexneri* is antagonised by the bacterial effector IpaH9.8

The increased UFM1 decoration of *S. flexneri* Δ*mxiE* (Fig. 2c, d) during infection led us to hypothesise that additional bacterial effectors may intersect with the UFMylation cascade beyond UFL1 recruitment. Among T3SS effectors, members of the Invasion Plasmid Antigen H (IpaH) family of proteins have been shown to have important roles in counteracting cell-autonomous immunity mechanisms^47–49^. Importantly, a Yeast-Two-Hybrid screening for the *S. flexneri* secreted effector IpaH9.8 identified UFM1 as a potential interaction partner (Supplementary Table 1), suggesting that IpaH9.8 may prevent UFM1 association with *S. flexneri*.

**Fig. 3:**
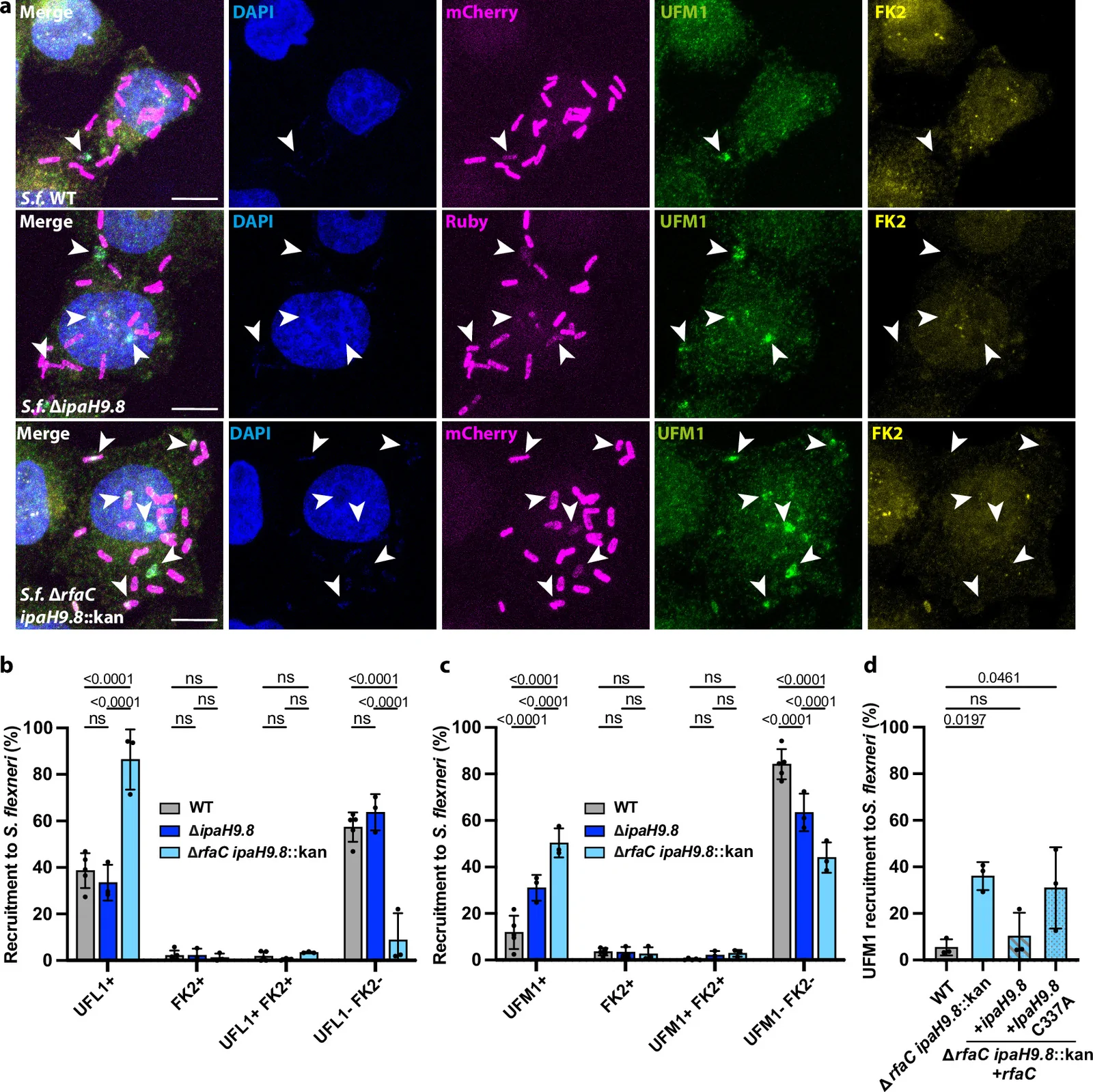
*Shigella* effector IpaH9.8 prevents UFM1 conjugation. **a** Representative confocal images of HeLa cells infected with mCherry *Shigella* WT, Ruby *Shigella* Δ*ipaH9.8* and mCherry *Shigella* Δ*rfaC ipaH9.8*::kan at 3 h.p.i. Arrows show UFM1 recruitment to bacteria. Scale bar, 10 µm. **b** Percentage of mCherry *Shigella* WT (*N* = 5 independent experiments, *n* = 1311), Ruby *Shigella* Δ*ipaH9.8* (*N* = 3 independent experiments, *n* = 463) and mCherry *Shigella* Δ*rfaC ipaH9.8*::kan (*N* = 3 independent experiments, *n* = 553) colocalising with UFL1 and ubiquitin (FK2) at 3 h.p.i. **c** Percentage of mCherry *Shigella* WT (*N* = 5 independent experiments, *n* = 882), Ruby *Shigella* Δ*ipaH9.8* (*N* = 3 independent experiments, *n* = 476) and mCherry *Shigella* Δ*rfaC ipaH9.8*::kan (*N* = 3 independent experiments, *n* = 526) colocalising with UFM1 and ubiquitin (FK2) at 3 h.p.i. UFL1 and UFM1 quantifications for mCherry WT *Shigella* same as Fig. 2f, g. **d** Percentage of *Shigella* WT (*n* = 843), Δ*rfaC ipaH9.8*::kan (*n* = 576) and complemented mutants Δ*rfaC ipaH9.8*::kan + rfaC + ipaH9.8 (*n* = 559) and Δ*rfaC ipaH9.8*::kan + rfaC + ipaH9.8C337A (catalytically dead, *n* = 654) colocalising with UFM1 at 3 h.p.i. **b**,** c** Two-way ANOVA and Tukey’s test, two-sided. **d** One-way ANOVA and Dunnett’s test, two-sided. Results represented as mean ± SD. n.s: non-significant. Source data are provided as Source Data file.

To test a role for IpaH9.8 in counteracting UFM1 decoration of *S. flexneri*, we scored UFL1 and UFM1 recruitment to *S. flexneri* WT, Δ*ipaH9.8* and the double mutant Δ*rfaC ipaH9.8*::kan. While deletion of *ipaH9.8* did not affect UFL1 recruitment to *S. flexneri*, we observed a higher decoration of UFM1 in *S. flexneri* Δ*ipaH9.8* compared to WT at 3 h.p.i. (2.6 ± 0.5-fold), which further increased (4.4 ± 0.6-fold) in the case of the double mutant Δ*rfaC ipaH9.8*::kan (Fig. 3a, c, and Supplementary Fig. 4a, b, d–f**)**. At later timepoints (5 h.p.i.) the levels of UFM1 decoration were higher and non-significantly different for *S. flexneri* WT, Δ*ipaH9.8* and the double mutant Δ*rfaC ipaH9.8*::kan (37.8 ± 30.6%, 54.6 ± 26.7%, 60.6 ± 12.1%, respectively, Supplementary Fig. 4f). Consistent with previous reports^10^, deletion of *ipaH9.8* in the absence of IFN-γ did not impact *S. flexneri* ubiquitylation (Fig. 3b, c), further suggesting that UFMylation operates independently of the ubiquitin axis. Notably, the increased UFM1 decoration observed for *S. flexneri* Δ*rfaC ipaH9.8*::kan returned to baseline levels by complementation with *rfaC* and *ipaH9.8*, but not with a catalytically inactive IpaH9.8 lacking E3 ligase activity (Fig. 3d). Together, these results indicate that the enzymatic activity of *S. flexneri* T3SS effector IpaH9.8 is required to obstruct UFM1 decoration of the bacterial surface during infection.

**Fig. 4:**
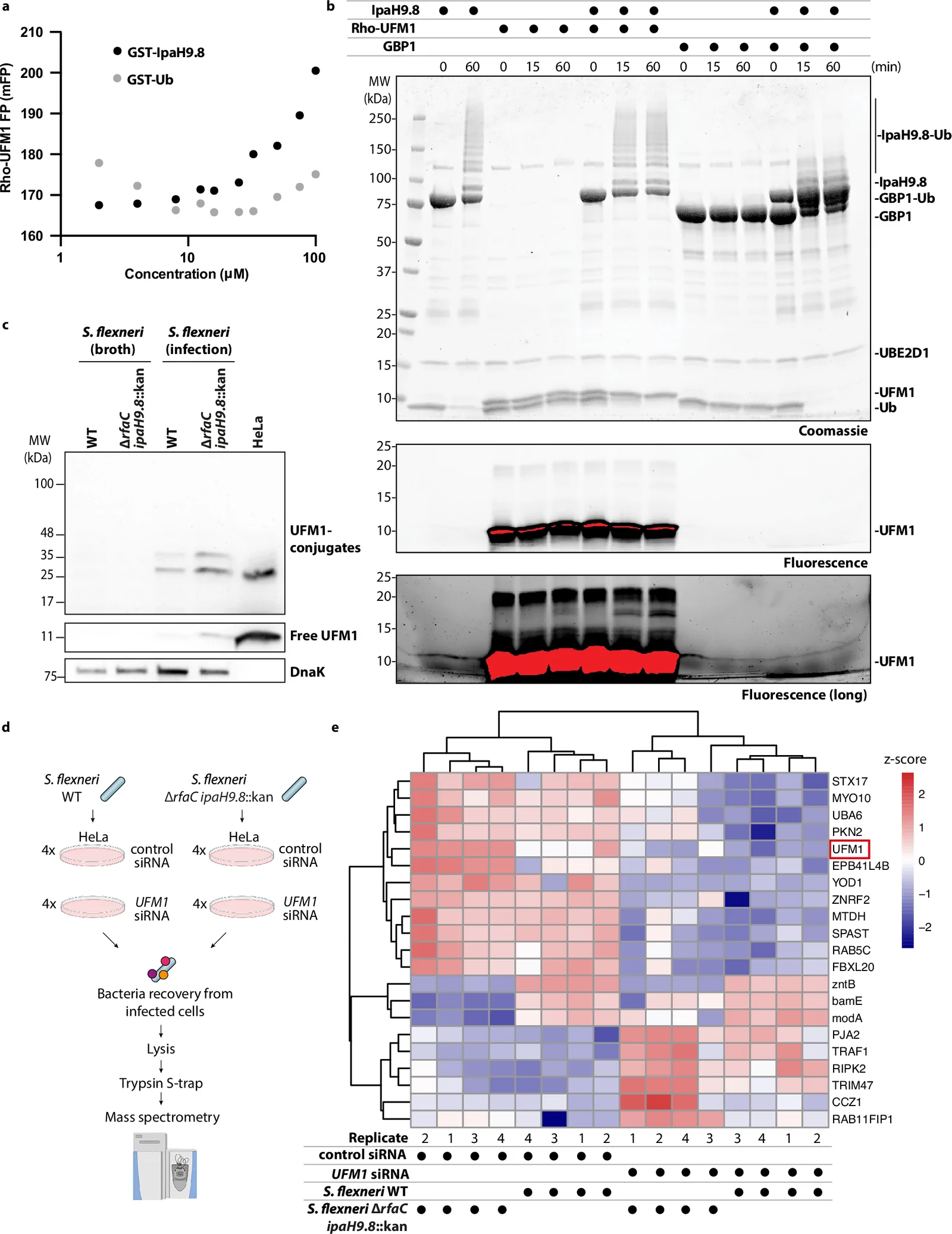
*Shigella* effector IpaH9.8 interacts with UFM1. **a** A Protein-protein interaction assay monitoring fluorescence polarisation (FP) of rhodamine-labelled UFM1 in response to increasing concentrations of recombinant GST-IpaH9.8. To control for possible interactions with the GST tag of GST-IpaH9.8, GST-ubiquitin is titrated instead. Mean from 2 independent experiments. **b** In vitro ubiquitylation assay using human UBA1 and UBE2D1 alongside IpaH9.8 (E3 ligase). Rhodamine-labelled UFM1 was assessed as a substrate for IpaH9.8-dependent ubiquitylation, alongside His-GBP1 (positive control). A low level of UFM1 ubiquitylation is observed in a long exposure fluorescence image. GBP1 ubiquitylation by IpaH9.8 is used as a positive control. Representative gel from 2 independent experiments. **c** Western blot showing UFM1-conjugates associated with *Shigella* WT expressing AfaI and *Shigella* Δ*rfaC ipaH9.8*::kan bacterial pellets isolated from HeLa cell infection (3 h.p.i), that are absent in bacteria grown in broth. UFM1 species present in uninfected HeLa cells are shown as a control. Different exposure times were used to detect free UFM1 (low) and UFM1-conjugates (high) due to relative differences in abundance, using anti-UFM1 antibodies (Abcam). Representative blot from 2 independent experiments. **d** Workflow to identify proteins from *Shigella* WT expressing AfaI and *Shigella* Δ*rfaC ipaH9.8*::kan mutant recovered from control or *UFM1*-depleted HeLa cells at 3 h.p.i. using mass spectrometry. Created in BioRender. Lopez, A. (2026) https://BioRender.com/qa8tfz4. **e** Heat map showing selected, significantly regulated proteins associated with *Shigella* WT expressing AfaI and *Shigella* Δ*rfaC ipaH9.8*::kan in control and *UFM1*-depleted HeLa cells, 4 biological replicates. Proteins identified by LC-MS/MS and their intensities between control and *UFM1-*depleted HeLa cells were compared using |log2(fold change)| ≥ 2 and adjusted *P* value ≤ 0.05. *Shigella* proteins in lowercase, human proteins in uppercase. Source data are provided as Source Data file.

To further investigate how IpaH9.8 modulates UFMylation during *S. flexneri* infection, we tested whether IpaH9.8 directly interacts with UFM1. Binding studies using purified proteins confirmed a weak but direct interaction between GST-tagged IpaH9.8 and UFM1 (Fig. 4a). As a control, GST-tagged ubiquitin showed no interaction with UFM1. Given that the E3 ligase activity of IpaH9.8 is required to antagonise UFMylation (Fig. 3d), we hypothesised that UFM1 itself may be a substrate for IpaH9.8-mediated ubiquitylation, similar to previously described targeting of GBP1^10,11^. To test this, we performed an in vitro ubiquitylation assay (Fig. 4b). As expected, IpaH9.8 efficiently promoted time-dependent ubiquitylation of GBP1. In contrast, ubiquitylation of UFM1 was only weakly detected under these conditions, suggesting that either it is not a direct substrate, or additional host factors or specific conjugated forms of UFM1 may be required for efficient modification.

To determine whether UFM1 is associated with bacteria during infection, we isolated bacteria from infected cells and probed for the presence of UFM1-conjugates by western blot (Fig. 4c). We detected several UFM1-positive bands that were absent in bacteria from broth, indicating UFM1-conjugates at the bacterial surface. We observed a similar pattern in a *S. flexneri* double mutant Δ*rfaC ipaH9.8*::kan (Fig. 4c), suggesting that LPS is not the bacterial substrate for UFMylation and that IpaH9.8 does not prevent conjugation of specific substrates. To characterise these UFM1 substrates, we analysed *S. flexneri* WT and Δ*rfaC ipaH9.8*::kan isolated from HeLa cells treated with control or *UFM1* siRNA by proteomics (Fig. 4d, e and Supplementary Data File 3). As an internal control, RfaC and IpaH9.8 were markedly reduced in *S. flexneri* Δ*rfaC ipaH9.8*::kan compared to WT, consistent with their genetic deletion. UFM1 was detected on bacteria isolated from infected cells, indicating increased abundance in the *S. flexneri* Δ*rfaC ipaH9.8*::kan mutant in agreement with our microscopy data (Fig. 3a, c), and reduced abundance upon *UFM1* depletion. These findings support enrichment of UFM1-conjugates on isolated bacteria. We were unable to confidently identify UFMylation-specific K-ε-VG remnants on bacterial or host proteins (similar to the K-ε-GG remnant of ubiquitylation^50^) and could not conclude on the precise identity of UFMylated substrates. Nevertheless, we detected differential recruitment of host proteins to *S. flexneri* depending on the presence of UFM1 (Fig. 4e). Together, these results suggest that UFM1 decoration of *S. flexneri* is antagonised by the E3 ligase activity of IpaH9.8, altering the repertoire of proteins recruited to bacteria.

### UFMylation has a conserved antibacterial role independent of autophagy

Considering the ubiquitous expression of the UFMylation pathway in human cells (Human Cell Atlas) and its high conservation among eukaryotes^28^, we decided to test the localisation of UFL1 and UFM1 to *S. flexneri* in THP-1 macrophages and zebrafish larvae. In both cases, UFL1 and UFM1 localised to *S. flexneri* (Supplementary Fig. 5), suggesting a broad and conserved role during infection.

**Fig. 5:**
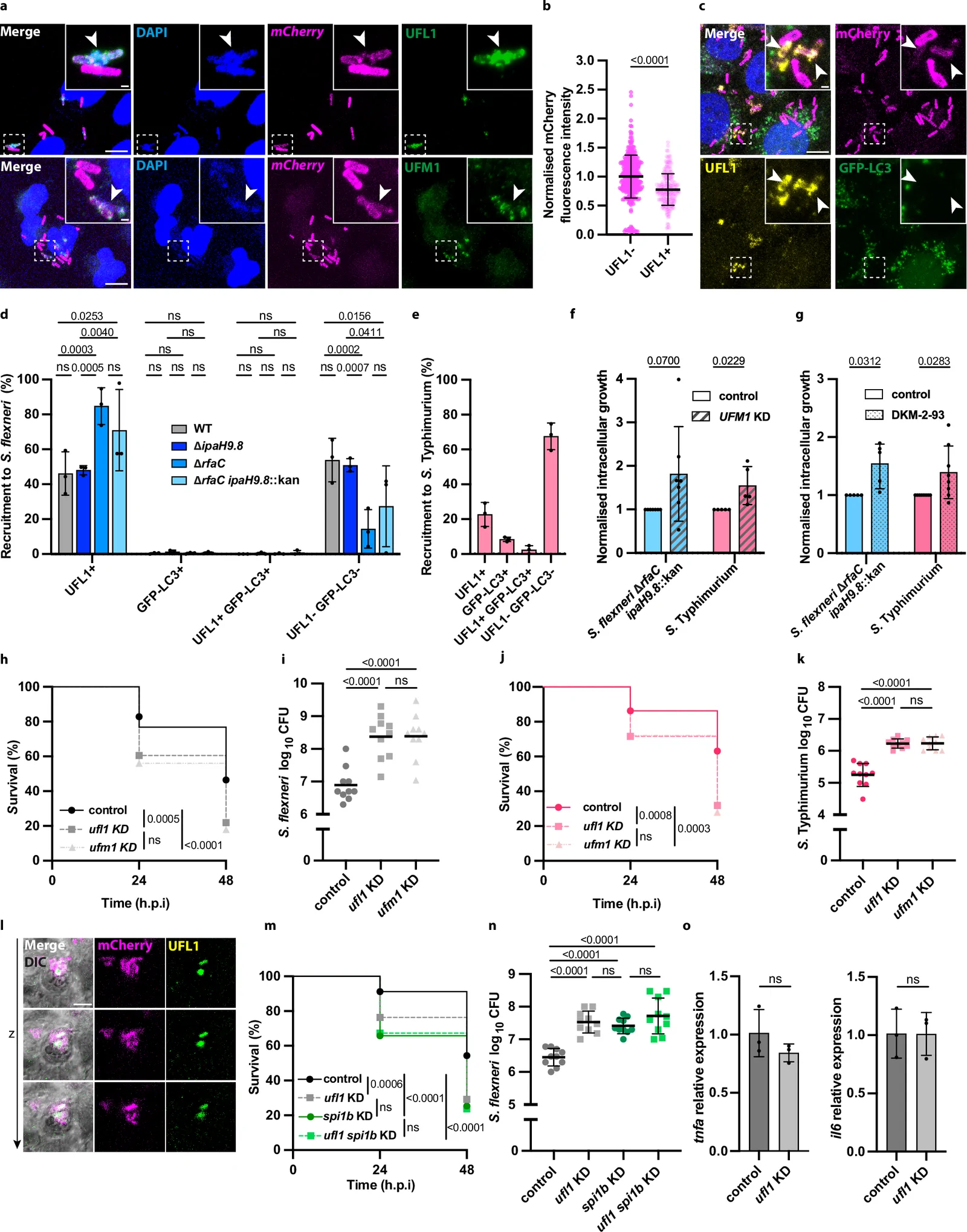
UFMylation has a conserved antibacterial role independent of autophagy. **a** Confocal images of HeLa cells infected with mCherry *Shigella* (3 h.p.i., 3 independent experiments). Arrows show UFL1/UFM1-positive bacteria with decreased mCherry fluorescence. Scale bar, 10 μm; inset, 1 μm. **b** mCherry fluorescence in UFL1-positive (*n* = 209) and UFL1-negative (*n* = 348) *Shigella*, 3 independent experiments. **c** Confocal image of GFP-LC3-expressing HeLa cells infected with mCherry *Shigella* (3 h.p.i, 3 independent experiments). Arrows indicate UFL1-positive and LC3-negative *Shigella* with decreased mCherry fluorescence. Scale bar, 10 μm; inset, 1 μm. **d**,** e** Quantification of UFL1 and GFP-LC3 recruitment to mCherry *Shigella* WT (*n* = 655), Ruby *Shigella* Δ*ipaH9.8* (*n* = 733), mCherry *Shigella rfaC*::kan (*n* = 422), mCherry *Shigella* Δ*rfaC ipaH9.8*::kan (*n* = 627) and *Salmonella* (*n* = 582) at 3 h.p.i., 3 independent experiments. **f**,** g** *Shigella* Δ*rfaC ipaH9.8*::kan and *Salmonella* intracellular growth in *UFM1* knockdown HeLa cells (*N* = 7, *N* = 5), or treated with UBA5 inhibitor DKM 2-93 (*N* = 5, *N* = 8) normalised to control, 5 h.p.i. **h, j**, Zebrafish survival after *Shigella* or *Salmonella* caudal vein infection. WT larvae (46.40%, *n* = 76 / 63.15%, *n* = 54), *ufl1* (21.85% *n* = 81 / 31.81%, *n* = 61) and *ufm1* (18.00%, *n* = 70 / 27.91%, *n* = 55) KD crispants, 2 independent experiments. **i**,** k** *Shigella* and *Salmonella* recovered from infected WT larvae, *ufl1* and *ufm1* KD crispants (*n* = 10 each, 2 independent experiments), 48 h.p.i. **l** Zebrafish tail fin infected with *Shigella* showing UFL1 recruitment to intracellular bacteria (representative of *n* = 6), 2 h.p.i. The arrow indicates the z-planes of the same stack. Scalebar, 5 μm. **m** Zebrafish survival following *Shigella* caudal vein infection for WT larvae (54.39%, *n* = 114), *ufl1* (29.21%, *n* = 89), *spi1b* (25.32%, *n* = 79), and *spi1b ufl1* (23.76%, *n* = 101) KD crispants, 2 independent experiments. **n** *Shigella* recovered from infected WT larvae, *ufl1*, *spi1b*, and *spi1b ufl1* KD crispants at 48 h.p.i. (*n* = 10 each, 2 independent experiments). **o** *tnfa* and *il6* RT-PCR in control or *ufl1-*depleted larvae. **b**,** f**,** g**,** o** Two-sided Student’s t-test. **d** Two-way ANOVA and Tukey’s test, two-sided. **h**,** j**,** m** Two-sided Log rank (Mantel-Cox) test. **i**,** k**,** n** One-way ANOVA and Tukey’s test, two-sided. Results represented as mean ± SD. n.s: non-significant. Source data are provided as Source Data file.

We next tested whether UFMylation has an antibacterial role, similar to ubiquitylation. We observed that intracellular *S. flexneri* with a strong recruitment of UFL1 or UFM1 displayed a fluorescence loss of their mCherry constitutive reporter, suggesting UFMylated bacteria were subjected to degradation (Fig. 5a). Indeed, the mean fluorescence signal for mCherry was reduced 22% upon UFL1 recruitment in HeLa cells (Fig. 5b), and similar loss of fluorescence was also observed in THP-1 macrophages and zebrafish larvae (Supplementary Fig. 5). Considering ubiquitylation classically targets intracellular bacteria to degradation by the autophagic machinery, we assessed the colocalisation of UFMylation and the main autophagic marker GFP-LC3 during infection. In the case of *S. flexneri*, we observed no overlap between UFL1 recruitment and GFP-LC3 engulfment of intracellular bacteria (Fig. 5c, d), consistent with ubiquitylation results. *S. flexneri* mutants with increased UFL1 and/or UFM1 colocalisation (Δ*ipaH9.8*, *rfaC*::kan, Δ*rfaC ipaH9.8*::kan) did not increase the number of bacteria in autophagosomes (Fig. 5d). In the case of *S*. Typhimurium, most of the bacteria entrapped in an autophagosome also did not recruit UFL1 (Fig. 5e). Together, these results suggest that contrary to ubiquitylation, targeting of intracellular bacteria by the UFMylation machinery leads to bacterial degradation in a process independent of autophagy.

To further investigate the antibacterial role of the UFMylation pathway, we assessed its impact on *S. flexneri* and *S*. Typhimurium burden in cells (Fig. 5f, g, Supplementary Figs. 6 and 7). Depletion of *UFM1* using siRNA did not lead to an increase in intracellular bacterial replication nor bacterial spread in the case of *S. flexneri*, consistent with its capacity to antagonise the UFMylation pathway (Supplementary Fig. 6). *S. flexneri* Δ*rfaC ipaH9.8*::kan exhibited more pronounced growth upon *UFM1* depletion, but it was non-significant (*P* value = 0.070), suggesting that incomplete depletion of *UFM1* was insufficient or that additional effectors may counteract this pathway. In the case of *S*. Typhimurium, a vacuolar pathogen with a limited arsenal for cytosolic survival, we observed a significant increase in intracellular replication upon *UFM1* knockdown (Fig. 5f and Supplementary Fig. 6), which was amplified at later time points (Supplementary Fig. 6e). We interrogated whether these results may be amplified upon stimulation of IFN-γ, however, IFN-γ did not induce expression of *UFL1* nor *UFM1* in HeLa cells, nor did it significantly impact bacterial burden upon *UFM1* depletion (Supplementary Fig. 7a, b). Notably, pharmacological inhibition of UBA5 (the E1 enzyme of the UFMylation pathway) with DKM 2–93^51^ did not affect *S. flexneri* WT burden but led to a significant increase in intracellular replication of both *S. flexneri* Δ*rfaC ipaH9.8*::kan and *S*. Typhimurium (Fig. 5g and Supplementary Fig. 7). Together with the loss of fluorescence of UFMylation targeted cells, these results indicate that UFMylation plays a host defence role against cytosolic bacteria.

Finally, we interrogated the role of the UFMylation pathway in vivo using the zebrafish infection model. Depletion of *ufm*1 and *ufl1* did not affect larvae development (Supplementary Fig. 8a), but significantly reduced zebrafish larvae survival during infection, both in the case of *S. flexneri* and *S*. Typhimurium (Fig. 5h, j and Supplementary Fig. 8b–f). This increased susceptibility correlated with increased bacterial load of more than an order of magnitude in both cases (Fig. 5i, k). In agreement with UFL1 recruitment to *S. flexneri* in human tissue culture cells (HeLa, THP-1; Fig. 2a, e and Supplementary Fig. 5), we observed UFL1 recruitment to *S. flexneri* within individual infected cells in vivo (Fig. 5l and Supplementary Fig. 9). To test the role of professional immune cells, we depleted the Pu.1 transcription faction (*spi1b*) essential for myeloid lineage development^52^. *spi1b* depletion phenocopied the *ufl1* knockdown phenotype and had no additive effect when combined with *ufl1* depletion (Fig. 5m, n), consistent with macrophages and neutrophils being critical for controlling *S. flexneri* infection in this infection model^53–56^. To exclude the possibility that observed antibacterial phenotypes resulted from altered inflammatory responses, we quantified expression of pro-inflammatory cytokines *tnfa* and *il6*. No significant differences were detected between WT and *ufl1*-depleted larvae (Fig. 5o), suggesting that increased bacterial burden and associated larvae mortality are not due to impaired inflammatory signalling. Collectively, these results provide evidence for an important and conserved defence role against bacterial infection and highlight the UFMylation pathway as a conserved / ancient cell-autonomous immunity mechanism (Supplementary Fig. 10).

## Discussion

Proximity mapping techniques have emerged as key discovery tools in a wide variety of research fields. For infection biology, recent applications include screenings for host-interacting partners of *Legionella*^57^ and *S*. Typhimurium^58^ secreted effectors, as well as the surface proteome (including host-bound proteins) of *Vibrio cholerae* recovered from rabbit diarrhoeal fluid^59^. Here, we pioneered a proximity biotinylation approach to specifically map the host-bacterial interface during infection. This strategy is particularly relevant to understand the proteomic microenvironment of cytosol-dwelling bacteria, since the isolation and proteomic characterisation of bacterial-containing vesicles (BCVs) has been performed successfully for multiple vacuolar pathogens^60–64^. In addition, our proximity biotinylation approach is highly versatile, as it is functional in tissue culture cells and in animal models, and it can be easily adapted to any other genetically tractable Gram-positive or negative bacteria.

Our proximity biotinylation screening identified multiple E3 ligases in close proximity to *S. flexneri* during infection. Among these, RNF213 was found enriched in bacteria lacking MxiE, an important transcriptional regulator for the expression of late T3SS effectors. Consistent with these results and serving as an internal positive control for our screening, multiple studies have recently reported that *S. flexneri* deploys the bacterial effector IpaH1.4 (under MxiE transcriptional control) to target RNF213 to degradation^18–20^. The proximity interactome of *S. flexneri* also revealed UFL1, the only identified E3 ligase of the UFMylation pathway, the latest UBL system discovered.

Our work has shown that both UFL1 and its substrate peptide UFM1 are recruited to the surface of *S. flexneri* and *S*. Typhimurium during infection, which showed little to no overlap with bacterial ubiquitylation. The *S. flexneri* surface became associated with UFM1 during infection, as bacteria recovered from infected cells presented UFM1-conjugates absent in bacteria from broth. In contrast to RNF213, which ubiquitylates LPS^14^, the nature of the UFMylated substrates during *S. flexneri* infection is likely proteinaceous, as they appeared as discrete, well-defined bands on a western blot, although whether it is of host or bacterial origin remains to be established. Together, these findings indicate that bacterial ubiquitylation by RNF213 and UFMylation by UFL1 are separate pathways that protect the host cytosol in a multilayered manner.

Consistent with a functional role of UFMylation at the host–bacteria interface, proteomic analysis of *S. flexneri* isolated from HeLa cells revealed differential association with host proteins upon depletion of *UFM1*, suggesting that UFM1-conjugates associated with *S. flexneri* may act as a platform that promotes recruitment of specific downstream effectors, similarly to ubiquitylation^39^; investigating how these may contribute to bacterial restriction is of great interest. The detection of UFM1 in this second proteomic dataset but not in our original proximity biotinylation approach likely reflects differences in sample preparation and enrichment affecting recovery of this low–molecular–weight modifier.

*S. flexneri* has evolved strategies to avoid targeting by the UFMylation machinery, which underscores its relevance as an antibacterial pathway. First, the S*. flexneri* mutant *rfaC*::kan with shortened LPS showed an increased recruitment of UFL1, indicating that the LPS can shield intracellular bacteria from host recognition. It is worth noting that *S. flexneri rfaC*::kan is also more susceptible to septin cage entrapment^40^. Collectively, these results suggest that the protective role of the LPS may contribute not only to avoiding extracellular recognition^65,66^, but also intracellular detection. Likely reflecting the host-pathogen arms race, host cells also evolved to recognise intracellular LPS by caspase-4/-11^67^ and the TRAF-interacting forkhead-associated protein A (TIFA)^68,69^ for inflammasome and NF-κB activation, respectively. In this context, it is not surprising that bacterial pathogens, including *S. flexneri*, tightly modulate their LPS production during infection^70,71^.

A second strategy deployed by *S. flexneri* to counteract UFMylation is the secreted effector IpaH9.8, which did not impact UFL1 recruitment to the bacteria but their decoration with UFM1. This adds to the list of known functions of IpaH9.8, which has been shown to dampen the host inflammatory response^72,73^ and interfere specifically with established cell-autonomous immune programmes by targeting GBPs to proteasomal degradation^10,11^. Here we show that IpaH9.8 can directly interact with UFM1 and that its E3 ligase activity is required to antagonise UFM1 association with *S. flexneri*. However, in vitro ubiquitylation assays revealed only limited ubiquitylation of UFM1, consistent with the lack of overlap between UFM1 and ubiquitin decoration of *S. flexneri* by microscopy. This could indicate that IpaH9.8 ubiquitylates UFM1 at low levels or selectively targets a defined pool of UFM1, such as substrate-conjugated UFM1, or another substrate. In addition, this activity may depend on other host factors, as in the case for ABIN-1-dependent ubiquitylation of NEMO by IpaH9.8^73^, or the E2-dependent interaction of OspG with ubiquitin^74^. Ubiquitylation of UFM1 by IpaH9.8 would represent a process analogous to ubiquitylation of GBP1^10^ and distinct from direct RNF213-mediated ubiquitylation of the *S. flexneri* surface. In this context, UFM1 decoration of *S. flexneri* is independent of ubiquitylation, consistent with bacteria expressing the effector IpaH1.4, which targets RNF213 for degradation^18–20^. Future structural and biochemical studies will delve into the specificity and molecular mechanism underlying the IpaH9.8 – UFM1 interaction.

Our work has revealed UFMylation as a cell-autonomous immune pathway against cytosolic bacteria. This pathway is active during infection of human cells (epithelial cells and THP-1 macrophages) and also in zebrafish larvae. Together with the high degree of conservation of the UFMylation cascade among eukaryotes^28^, this strongly suggests a broader antibacterial role across evolution. Remarkably, UFMylation has very recently been shown to participate in the regulation of interferon and NF-κB-mediated responses during viral and bacterial infections^35,75–78^, which may support the different susceptibility to *S. flexneri* and *S*. Typhimurium observed in cellulo and in vivo. Taken together, the UFMylation machinery should now be recognised as a major contributor to host defence.

## Methods

### Reagents

The following primary antibodies were used: mouse anti-Ubiquitinylated proteins, clone FK2 (Sigma, #04-263), mouse anti-CD107a (LAMP1, BD Biosciences, #555798), rabbit anti-UFL1 (Proteintech, #26087-1-AP), rabbit anti-SEPT7 (IBL, #18991), mouse anti-HA-Tag (6E2) (Cell Signalling, #2367), rabbit anti-Myc-Tag (71D10) (Cell Signalling, #2278), mouse anti-GFP (Abcam, #ab1218) rabbit anti-*Shigella* group antigen (Bio-Rad, #8289-9506), rabbit anti-protein disulphide isomerase (Proteintech, #11245-1-AP). Rabbit anti-UFM1 (Proteintech, #15883-1-AP) was used for immunofluorescence, and rabbit anti-UFM1 (Proteintech, #15883-1-AP; Abcam #109305) was used for western blot as indicated in the figure legends. The following secondary antibodies were used: Alexa Fluor 488, 555 and 647-conjugated goat anti-mouse antibodies (Invitrogen #A-11001, #A-21424 and #A-21236), and Alexa Fluor 488, 555 and 647-conjugated goat anti-rabbit antibodies (Invitrogen, #A-11008, #A-21428, # A-21244). The following reagents were used: HRP-conjugated streptavidin (Invitrogen, #S911), Alexa Fluor 647-conjugated phalloidin (Invitrogen, #A22287), Congo red (Sigma-Aldrich, #C6767), IFN-γ (R&D Systems, #285-IF), DKM 2–93 (Cayman Chemical, #CAY37293) and Nalidixic acid (Sigma, #N8878).

### Bacterial strains and culture conditions

The bacterial strains and plasmids described in this study are listed in Supplementary Tables 2 and 3. *Shigella flexneri* 5a str. M90T was grown in trypticase soy broth (TSB) agar containing 0.01% of Congo red to select for red colonies, indicative of a functional T3SS. Conical polypropylene tubes containing 5 ml of TSB were inoculated with individual red colonies of *S. flexneri* and were grown ~16 h at 37 °C with shaking at 200 rpm. The following day, bacterial cultures were diluted in fresh prewarmed TSB (1:50 or 1:100 v/v) and cultured until an optical density (OD, measured at 600 nm) of 0.6.

For *Salmonella enterica* subsp. *enterica* srv. Typhimurium str. SL1344, single bacterial colonies were grown in conical polypropylene tubes containing 5 mL of LB for ~16 h at 37 °C in static conditions. The following day, bacteria were subcultured in fresh prewarmed LB (1:50 or 1:100 v/v) and grown for 3 h.

### Mammalian cell culture

HeLa cells (ATCC #CCL-2) were cultured in Dulbecco’s modified Eagle Medium (DMEM, GIBCO) in the presence of 10% heat-inactivated foetal bovine serum (hi-FBS, Gibco, #10500064) under standard conditions (37 °C and 5% CO_2_). GFP–LC3B-producing HeLa cells^79^ were grown as above.

THP-1 cells (male monocytes, ATCC #TIB-202) were grown in RPMI-1640 medium supplemented with 10% hi-FBS at 37 °C and 5% CO_2._ THP-1 cells were differentiated via phorbol 12-myristate 13-acetate (PMA, Sigma-Aldrich, #P8139) treatment^80^ into macrophages using 100 nM PMA in RPMI-1640 with 10% hi-FBS for 48 h followed by 24 h recovery phase in RPMI with 10% hi-FBS.

### Cloning

Plasmids used or generated in this study are listed in Supplementary Table 3. Primers used in this study were designed using Benchling (https://benchling.com) or NEBuilder Assembly Tool (https://nebuilder.neb.com/#!/), were synthesised by Sigma and are listed in Supplementary Table 4.

For GFP-APEX2 purification, the pTRC-GFP-APEX2 plasmid was generated by Gibson assembly using pTRC-APEX2^38^ and pLVX-msGFP–SEPT6^40^ as templates.

For Yeast-Two-Hybrid experiments, DNA fragment encoding IpaH9.8 C337A was amplified by PCR from plasmid pJR006^47^ and cloned as an EcoRI-BamHI fragment into the vector pGKBT7 to create the Yeast-Two-Hybrid bait vector pJB179-10.

For complementation assays, DNA fragments encoding IpaH9.8 and IpaH9.8 C337A were amplified by PCR and cloned as an EcoRI-HindIII fragment into the vector pUC19 to create pUCipaH9.8 and pUCipaH9.8C337A.

*S. flexneri* mutants were engineered using λ-Red-mediated recombination^81^. In brief, kanamycin resistance-encoding DNA cassettes were amplified using the pKD4 plasmid as template and primers containing 50 bp nucleotides homologous to the site of insertion. Resulting DNA fragments were electroporated in *S. flexneri* electrocompetent cells producing λ-Red recombinase and plated in TSA plates supplemented with 0.01% of Congo red and 50 μg/ml kanamycin. All strains were verified by PCR as shown in Supplementary Fig. 4.

*S*. Typhimurium was transformed with the pmCherry plasmid, containing an *mCherry* gene controlled under a *P*_*BAD*_ arabinose-inducible promoter. *mCherry* was PCR-amplified from a pRK2-mCherry plasmid^82^ and cloned between ZraI and SpeI restriction sites in a pFUS-P_BAD_ plasmid^83^.

### siRNA treatment

HeLa cells were transfected using siRNA targeting *UFM1* (Thermo Fisher Scientific #148645) or using negative control siRNA (Thermo Fisher Scientific, #4390843). HeLa cells were seeded in a 6-well plate, and 200 nM of siRNA was transfected per well using Oligofectamine (Invitrogen, #12252011) as described by the manufacturer. Cells were incubated for 48 h to ensure sufficient knockdown of targeted proteins.

### DNA transfections

HeLa cells were transfected using jetPEI transfection reagent (Polyplus, #101000053) according to the manufacturer’s instructions. Cells were incubated for 24 h before the experiment.

### Protein purification

For the in vitro binding of APEX2 to anti-GFP nanobodies displayed on the surface of *S. flexneri*, a monomeric superfolder version of GFP was added between the His_6_ tag and the N-terminus of APEX2 in the pTRC-APEX2 plasmid. GFP-APEX2 was purified as described previously^38^. Briefly, *Escherichia coli* BL21-DE3 cells transformed with the pTRC-GFP-APEX2 plasmid were grown to an OD (600 nm) of 0.5 in LB supplemented with 10 μg/mL ampicillin at 37 °C with shaking at 200 rpm. Protein expression was induced with 1 mM IPTG (Sigma-Aldrich, #I5502) and supplemented with 1 mM 5-aminolevulinic acid hydrochloride (Sigma-Aldrich, #A7793) overnight at room temperature to promote heme incorporation. Harvested cells were lysed using B-PER in the presence of 1 mM PMSF (Thermo Fisher Scientific, #36978) and cOmplete™ Protease Inhibitor Cocktail (Roche, #4693116001). The clarified lysates were incubated with Ni-NTA agarose chromatography matrix (Qiagen, #30210) for 30 min and loaded on an Econo-Pac column (Bio-Rad, #7321010) using gravity flow at 4 °C. The matrix was washed with binding buffer (50 mM Tris-HCl, 300 mM NaCl, pH 7.8) and washing buffer (binding buffer with 30 mM imidazole). GFP-APEX2 was eluted in binding buffer containing 200 mM imidazole. Purified protein was dialysed in PBS at 4 °C and concentrated using a 30 kDa Amicon Ultra centrifugal filter unit (Millipore, #Z648035).

Expression plasmids for GST-IpaH9.8 and GST-ubiquitin, both in the pGEX6P-1 vector, were gifts from Dr. Neal Alto and Dr. David Komander, respectively. The expression plasmid for His-GBP1 in the pETM11 vector was a gift from Dr. Felix Randow. Protein expression was performed as described above, with the exception of using Rosetta *E. coli* induced with 0.2 mM IPTG overnight at 18 °C. Cells were resuspended in 25 mM Tris-HCl, 200 mM NaCl, 2 mM *β*-mercaptoethanol, pH 8.0 and lysed by sonication in the presence of lysozyme, DNase, and protease inhibitor cocktail. The clarified lysates were loaded onto GST agarose (Pierce) or HisPur cobalt resin (Thermo Fisher) in an Econo-Pac column for 60 min at 4 °C. The matrix was washed with the lysis buffer and eluted in lysis buffer containing 50 mM glutathione (IpaH9.8 and Ub) or 250 mM imidazole (GBP1). Purified protein was dialysed in 25 mM Tris-HCl, 200 mM NaCl, 2 mM *β*-mercaptoethanol, pH 8.0 at 4 °C, concentrated using a 30 kDa Amicon Ultra centrifugal filter unit (Millipore, #Z648035), and quantified by absorbance at 280 nm prior to flash freezing and storage at −80 °C.

### In vitro coating of bacteria with GFP-APEX2

*S. flexneri* WT or Δ*mxiE* mutant carrying pNVgfp plasmid for nanobody display on the bacterial surface^37^ and pAC*afaI* plasmid to promote invasion of HeLa cells^84^ were grown overnight in the presence of the appropriate antibiotics. The following day, bacterial cultures were diluted 1:50 and grown until OD (600 nm) 0.6 in the presence of the appropriate antibiotics and 1 mM of IPTG. 1.5 mL of bacterial culture was washed twice in PBS and incubated in 75 μL of PBS containing 300 nM of purified GFP-APEX2 at room temperature on rotation for 1 h. Bacteria were washed twice with PBS to remove unbound GFP-APEX2 and used for subsequent infection assays.

### Proximity biotinylation

Proximity biotinylation was adapted from previously published protocols^85^. 6 μM hemin-Cl (Sigma-Aldrich, #H9039) was added to all samples for the duration of the experiment to increase functionality of purified GFP-APEX2. For the biotinylation reaction, 500 μM of biotin-phenol (Sigma-Aldrich, #SML2135) was added to the sample and incubated for 30 min. 1 mM of H_2_O_2_ (Sigma-Aldrich, #H1009) was added, and samples were incubated at 37 °C for 1 min (in vitro assay, in cellulo assay), or for 5 min (for zebrafish larvae). The biotinylation reaction was stopped by washing the samples in ice-chilled quencher buffer containing 10 mM sodium ascorbate (Sigma-Aldrich, #A92902), 5 mM trolox (Sigma-Aldrich, #238813) and 10 mM sodium azide (Thermo Fisher Scientific #190380050) three times. Cells were collected using a cell scraper for western blot analysis or purification of biotinylated proteins. For immunofluorescence, samples were first fixed with 4% paraformaldehyde (in PBS) at room temperature and proximity biotinylation was performed on fixed cells or zebrafish larvae to prevent diffusion of biotinylated proteins.

### Purification of biotinylated proteins

Purification of biotinylated proteins was performed as described before^85^. After proximity biotinylation, collected cells were centrifuged for 10 min at 3000 x *g* at 4 °C and lysed in RIPA buffer (750 μL for 10^7^ cells) supplemented with cOmplete™ Protease Inhibitor Cocktail, 1 mM PMSF and quenchers (10 mM sodium azide, 10 mM sodium ascorbate and 5 mM trolox) and incubated on ice for 2 min. Lysates were clarified by centrifugation at 15,000 x *g* for 10 min at 4 °C. Pierce™ Streptavidin magnetic beads (Thermo Fisher Scientific, #88816) were added to whole cell lysates (60 μL for 10^7^ cells) and incubated on rotation 1 h at room temperature. A magnetic rack was used to pellet the beads and collect the supernatant (flow-through). Beads were washed to remove nonspecific binders in ice-cold conditions twice with RIPA buffer, once with 1 M KCl, once with 0.1 M Na_2_CO_3_, once with 2 M urea in 10 mM Tris-HCl, pH 8.0, and twice with RIPA buffer again. Beads were snap frozen and subsequently analysed by mass spectrometry or alternatively processed for western blot analysis. For western blot, biotinylated proteins were eluted from the beads by boiling 50 μL of beads in 50 μL Laemmli buffer (3x) supplemented with 2 mM biotin and 20 mM DTT for 10 min and finally recovered from the beads using a magnetic rack.

### Proximity biotinylation sample preparation for LC-MS/MS analysis

4 × 10^7^ HeLa cells were treated with 1 ng/uL IFN-γ for 16 h and were infected with GFP-APEX2-coated S*. flexneri* WT or Δ*mxiE* at MOI 80 in quadruplicates. Proximity biotinylation was performed at 1.5 h.p.i., and biotinylated proteins were purified. Streptavidin beads were resuspended with 23 µL of a buffer containing 5% SDS (Merck, #L4509) with 100 mM triethylammonium bicarbonate (TEAB) (Merck, #T7408) at pH 8.0. Proteins were reduced and alkylated with 13 mM tris(2- carboxyethyl)phosphine (TCEP) (Thermo Fisher Scientific, #20491), combined with 40 mM chloroacetamide (CAA) (Merck, #C0267) and incubated for 10 min at 95 °C under agitation. Samples were acidified by adding 2.5 μL of 27% Phosphoric acid (final 2.5%, v/v) (Merck, #438081). Samples were mixed with 90% methanol (Thermo Fisher Scientific, #A456-212) and 100 mM TEAB and loaded onto S-TrapTM micro columns (≤100 μg) (ProtiFi, #C2-micro) according to the manufacturer’s protocol. Proteins were digested with 2 μg trypsin (#V5111, Promega) in 20 μL of 50 mM TEAB at pH 8.0 for overnight at 37 °C on the S-trap. Peptides were eluted by the sequential addition of three elution buffers containing: 50 mM TEAB (buffer 1), 0.2% formic acid (FA) (Thermo Fisher Scientific, #270480010) (buffer 2) and 50% acetonitrile (ACN) (Thermo Fisher Scientific, #A955-212) with 0.2% FA (buffer 3). Peptides were dried under reduced pressure and desalted a second time using an OMIX C18 pipette (#A57003MB, Agilent Technologies). Purified peptides were completely dried and stored at −20 °C until LC-MS/MS analysis. Peptides were re-dissolved in 33 µL loading solvent (0.1% TFA in H_2_O/ACN, 99.5:0.5, (v/v)) of which 2 µg of the sample measured on Dropsense16 (Unchained Labs) was injected for LC-MS/MS analysis on an Ultimate 3000 Pro Flow nanoLC system in-line connected to a Q Exactive HF mass spectrometer (Thermo Fisher Scientific).

### LC-MS/MS analysis for proximity biotinylation

Trapping was performed at 20 μL/min for 2 min in loading solvent on a 5 mm trapping column (Thermo Fisher Scientific, Pepmap, 300 μm internal diameter (I.D.), 5 μm beads). Peptides were separated on a 250 mm Aurora Ultimate, 1.7 µm C18, 75 µm inner diameter (Ionopticks) kept at a constant temperature of 45 °C. Peptides were eluted by a non-linear gradient starting at 0.5% MS solvent B (0.1% FA in ACN) and MS solvent A (0.1% FA in water). Gradient reached 33% at 70 min, 55% at 85 min and 70% at 90 min of MS solvent B. The gradient was followed by a 5-min wash at 70% MS solvent B and re-equilibration with MS solvent A for 15 min. The mass spectrometer was operated in data-dependent mode (DDA), automatically switching between MS and MS/MS acquisition with a top 12 method. Full-scan MS spectra ranging from 375–1500 m/z with a target value of 3E6, a maximum fill time of 50 ms and a resolution of 60,000 (at 200 m/z). Target accumulation was set at 3E6 for 60 ms in MS1. The 12 most intense ions above a threshold value of 1.3E4 were isolated in the trap with an isolation window of 1.5 Da for a maximum of 60 ms to a target AGC value of 1E3. Only precursor ions with a charge state equal to 2–6 were selected. Peptide match was set on “preferred”, and isotopes were excluded. Dynamic exclusion time was set to 12 s. Fragmentation was performed at a normalised collision energy of 30% (NCE) with HCD fragmentation. MS/MS spectra were acquired at a fixed first mass 145 m/z at a resolution of 15,000 (at 200 m/z) in the Orbitrap analyser. The MS1 spectrum data type was set to profile, MS2 spectrum data type was set to centroid. The polydimethylcyclosiloxane background ion at 445.12003 Da was used for internal calibration (lock mass), and QCloud was used to control instrument longitudinal performance during the project^86^.

### Data analysis for LC-MS/MS for proximity biotinylation

LC-MS/MS runs of all samples were searched using the MaxQuant algorithm (version 2.2.0.0)^87^. Spectra were searched against the protein sequences in the Swiss-Prot database (www.uniprot.org), from human (UP000005640_9606) and *Shigella flexneri* (strain 301 / Serotype 2a) (UP000001006_623) (downloaded in May 2023). Additionally, the database included the protein sequence for GFP-APEX2. Mainly, the default settings were used. The false discovery rate was set at 1% on the peptide (precursor) and protein level. Enzyme specificity was set as C-terminal to arginine and lysine, also allowing cleavage at proline bonds with a maximum of two missed cleavages. Variable modifications were set to oxidation of methionine residues, acetylation of protein N-termini, and Biotin of lysine residues. Matching between runs was turned OFF. Proteins were quantified with MaxLFQ intensities based on razor and unique peptides with a minimum of two ratio counts per protein. Further data analysis was performed with an in-house script in the R programming language by loading the proteinGroups table from MaxQuant. Briefly, proteins identified in the reverse database were removed. Protein expression matrices were prepared as follows: only proteins with intensity in three replicates were retained for analysis. Missing intensity values were imputed by randomly sampling from a normal distribution centred around each sample’s noise level. Fold change and adjusted *P*-value at FDR = 0.05 were calculated with the package Limma in R^88^. GO-term enrichment analysis was performed using the Term Enrichment Service from AmiGO^89^ (https://amigo.geneontology.org/amigo) and plotted using R.

### Bacterial growth assays in broth

*S. flexneri* functionalised with GFP-APEX2 was subcultured (1:200) in TSB or TSB supplemented with 500 μM of biotin-phenol and 6 μM hemin-Cl at 37 °C with shaking at 200 rpm. OD (600 nm) was measured every 30 min.

### Yeast-two-hybrid screening

The Yeast-Two-Hybrid bait vector pJB79-10 encoding IpaH9.8 C337A was transformed into the Matchmaker Gold strain of *Saccharomyces cerevisiae* (Takara). A high-complexity normalised human cDNA Mate and Plate library (Takara) was screened following the protocol provided by the Matchmaker Gold Yeast-Two-Hybrid System (Takara). Positive prey clones were identified by plasmid rescue and DNA sequencing according to previously described^90^.

### Fluorescence polarisation binding assay

The fluorescent substrate rhodamine-UFM1 (Rho-UFM1) was chemically synthesised as described previously^91^. Rho-UFM1, GST-IpaH9.8, and GST-ubiquitin were diluted to ‘2x’ concentrations in 25 mM Tris-HCl, 150 mM NaCl, 2 mM *β*-mercaptoethanol, 0.1 mg/mL BSA, pH 7.4. To set up the binding assay, 10 µL of 200 nM Rho-UFM1 was mixed with buffer or with 2x GST-tagged protein. The mixture was left to equilibrate in the dark at room temperature for 15 min, after which time it was loaded into black Greiner low-volume 384-well plates. Fluorescence polarisation values were measured on a BMG Labtech CLARIOstar plate reader equipped with 482–16 nm and 530–40 optic filters. A target polarisation value of 180 was set for free Rho-UFM1.

### In vitro ubiquitylation assay

Ubiquitylation assays were prepared by combining 0.25 µM UBE1, 2 µM UBE2D1, 4 µM IpaH9.8, 40 µM ubiquitin, 10 mM ATP, 10 mM MgCl_2_, and either 10 µM UFM1 or GBP1 in a reaction buffer containing 25 mM sodium phosphate (pH 7.4), 150 mM NaCl. Parallel reactions that omitted IpaH9.8 were used to control for E3 dependency. For UFM1, 9 µM unlabelled UFM1 was combined with 1 µM rhodamine-labelled UFM1^91^ for tracking ubiquitylation by fluorescence. Reactions were prepared with all components except for ATP, which was subsequently added after taking a 0-min sample. The reactions were incubated, with shaking, at 37 °C, and additional samples were collected at 15 and 60 min. Samples were quenched in reducing SDS sample buffer and resolved by SDS-PAGE prior to fluorescence scanning and Coomassie staining.

### Infection of HeLa cells

HeLa cells were seeded in 6-well plates (Thermo Fisher Scientific) at a confluency of 1.5 × 10^5^ cells/well 2 days prior to infection. Cell cultures were infected with *S. flexneri* strains as described previously^8^. Briefly, HeLa cells were infected with *S. flexneri* by spin-inoculation at 110 x *g* for 10 min at a multiplicity of infection (MOI) of 100:1 (bacteria:cell). Then, plates were placed at 37 °C and 5% CO_2_ for 30 min. Infected cultures were washed three times with PBS pH 7.4 to remove extracellular bacteria and incubated with fresh DMEM containing 10% hi-FBS and 50 µg/mL gentamicin at 37 °C and 5% CO_2_ for up to 5 h. Infections with *S. flexneri* mutants lacking *rfaC* (hyperinvasive) or expressing the adhesin AfaI were performed at MOI 10 in the absence of spin inoculation, unless indicated otherwise.

For *S*. Typhimurium infections, 50 µL of bacterial subculture was added to wells containing HeLa cells and incubated for 30 min at 37 °C for an MOI 80–100. Following three washes with PBS, infected cultures were incubated with fresh DMEM containing 10% FBS and 50 µg/mL gentamicin at 37 °C and 5% CO_2_ for up to 5 h. For inhibition of DNA synthesis or UBA5, infected cells were treated with 2 μg/mL Nalidixic acid or 5μM of DKM 2-93, respectively, after PBS washes to remove extracellular bacteria.

### Intracellular bacterial growth

HeLa cells seeded in 6-well plates and infected with *S. flexneri* were lysed in 1 ml cold PBS containing 0.1% Triton X-100. Serial dilutions were plated in duplicate on TCS agar containing 0.01% of Congo red for *S. flexneri* and LB agar for *S*. Typhimurium. Plates were incubated for 24 h at 37 °C, and colony-forming units (CFUs) were enumerated. CFUs counted at 4, 5 and 8 h.p.i. were normalised to 1 h.p.i. to assess intracellular growth.

### Bacteria recovery from infected cells

HeLa cells were seeded into 15 cm dishes at a confluency of 5.0 × 10^6^ and infected two days later with the hyperinvasive *S. flexneri* expressing AfaI or Δ*rfaC ipaH9.8*::kan at MOI 10. Extraction of bacteria from infected cells was performed as described before^14^. Cells were lysed in ice-cold PBS containing 0.1% Triton X-100 at 3 h.p.i. and centrifuged at 300 x *g* for 5 min at 4 °C. Supernatant containing bacteria was collected and centrifuged at 16,100 x *g* for 10 min at 4 °C. The bacterial pellet was washed once with PBS, followed by bacterial lysis in 75 μl BugBuster (Merck, #70584) including 2 mM iodoacetamide (Sigma, #I1149) for 5 min at room temperature. Samples were boiled after the addition of 25 μl of Laemmli buffer 4x for 10 min and analysed by western blot. For bacteria recovery intended for mass spectrometry analysis, same protocol was followed except that cell lysis was performed in PBS containing 0.1% Triton X-100, 2 mM iodoacetamide and cOmplete™ Protease Inhibitor Cocktail (Roche, #4693116001), and the bacterial pellet was washed once with PBS and lysed in 5% SDS in Tris 20 mM pH 7.5, boiled for 10 min, and stored at −80 °C until further processing. As control, 1 mL of bacteria in TSB was collected by centrifugation at OD (600 nm) 0.6, washed twice with PBS and lysed as described above.

### Sample preparation for LC-MS/MS analysis for bacteria recovered from infected cells

Protein amounts per sample were normalised to 10 µg in 23 µL with a buffer containing 5% SDS (Merck, #L4509) with 100 mM triethylammonium bicarbonate (TEAB) (Merck, #T7408) at pH 8.0. Proteins were reduced and alkylated with 5 mM tris(2- carboxyethyl)phosphine (TCEP) (Thermo Fisher Scientific, #20491), combined with 20 mM chloroacetamide (CAA) (Merck, #C0267) and incubated for 60 min at 37 °C without agitation in the dark. Samples were acidified by adding 2.5 μL of 27% phosphoric acid (final 2.5%, v/v) (Merck, #438081). Samples were mixed with 90% methanol (Thermo Fisher Scientific, #A456-212) and 100 mM TEAB and loaded onto S-TrapTM micro columns (≤100 μg) (ProtiFi, #C2-micro), and the columns were washed further according to the manufacturer’s protocol. Proteins were digested with 1 μg trypsin (#V5111, Promega) in 40 μL of 50 mM TEAB at pH 8.0 for overnight at 37 °C on the S-trap. Peptides were eluted by the sequential addition of three elution buffers with a volume of 40 µL. The three buffers contained: 50 mM TEAB (buffer 1), 0.1% formic acid (FA) (Thermo Fisher Scientific, #270480010) (buffer 2) and 50% acetonitrile (ACN) (Thermo Fisher Scientific, #A955-212) with 0.1% FA (buffer 3). Peptides were dried under reduced pressure, resuspended in 0.1% FA MS-grade water and desalted a second time using OMIX C18 pipette (#A57003MB, Agilent Technologies). Briefly, peptides were loaded on OMIX C18 tips, washed with 0.1% FA and eluted with 60% ACN in 0.1% FA. Purified peptides were completely dried and stored at −20 °C until LC-MS/MS analysis. Peptides were re-dissolved in 33 µL loading solvent (0.1% FA in H2O/ACN, 98:2, (v/v)) of which 1 µL was injected for LC-MS/MS analysis on a Vanquish Neo system in-line connected to an Orbitrap Astral mass spectrometer (Thermo Fisher Scientific).

### LC-MS/MS analysis for bacteria recovered from infected cells

Trapping was performed with the trap-and-Elute mode in combined control mode with a maximum threshold set at 60 µL/min for flow and 800 bar for pressure. Peptides were trapped on a 5 mm trapping column (Thermo Fisher Scientific, Pepmap, 300 μm internal diameter (I.D.), 5 μm beads) in a solvent with 0.1% TFA in water. Peptides were separated on an in-house produced column (75 µm x 100 mm) with a laser-pulled electrospray tip using a P-2000 Laser-Based Micropipette Puller (Sutter Instruments). The column was packed with ReproSil Saphir 100 1.5 µm silica particles (Dr. Maisch, #ra115.9e). The column was kept at a constant temperature of 45 °C. Peptides were eluted by a non-linear gradient starting at 0.5% MS solvent B (0.1% FA in ACN) and MS solvent A (0.1% FA in water). Gradient reached 26% at 17 min, 44% at 20 min and 56% at 21 min of MS solvent B. The gradient was followed by a 4-min wash at 56% MS solvent B and column equilibration in Pressure Control mode (separation column: fast equilibration, maximum pressure of 1500 bar, equilibration factor = 2; Trap column: fast wash and equilibration, wash factor = 100, trap wash in pressure control with a max pressure of 800 bar). The flow rate was kept at 250 or 300 nL/min during the gradient phase or column equilibration, respectively. The mass spectrometer was operated in data-independent analysis (DIA) mode. The MS1 scans were acquired at a resolution of 240,000 with a scan range of 380 − 980 m/z and an automatic gain control (AGC) of 500%. The MS2 scans were acquired at a resolution of 80,000 with a scan range of 150–2000 m/z. Precursor isolation was performed using a 2 m/z isolation window, a maximum injection time (maxIT) of 3 ms and an AGC target of 500%. The MS2 acquisition cycle covered a scan range of 380–980 m/z, producing 300 scans. Fragmentation was performed using higher-energy collisional dissociation (HCD) at 25% normalised collision energy (NCE). A loop control time of 0.6 s was applied. QCloud was used to control the instrument's longitudinal performance during the project^86^.

### LC-MS/MS data analysis for bacteria recovered from infected cells

LC-MS/MS runs of all samples were searched using DIA-NN (version 2.2.0)^92^. Spectra were searched against a custom protein database consisting of the human (UP000005640_9606) and *Shigella flexneri* (strain 301 / Serotype 2a) (UP000001006_623) UniProtKB reference proteomes (downloaded in January 2026), and the built-in contaminant protein used by DIA-NN. A predicted spectral library was generated with the following settings: peptide length 7–30, charge state 1–4, precursor mass range 380–980 m/z and Trypsin/P enzymatic digestion allowing a single missed cleavage. Cysteine carbamidomethylation was set as a fixed modification, and N-terminal methionine excision was enabled. For spectral library searches, mass accuracy was fixed to 10 ppm and 5 ppm for MS1 and MS2, respectively, while the scan window radius was set to 5. Matching between runs was turned on, and protein inference was turned off. The false discovery rate was set at 1% on precursor peptides. Further processing was performed with an in-house script in the R programming language by loading the precursor peptide table: “report.pr_matrix.tsv”. Briefly, peptides matching contaminant: “cRAP” were removed. Only peptides matching proteins identified by at least two peptide precursors were considered. Peptide intensities were normalised by the grand median method with the R package Qfeatures^93^. Only peptides identified in at least four samples were retained. Missing peptide intensities were imputed with values close to the limit of detection by the function impute.MinProb() (parameters q = 0.0001; tune.sigma = 1) with the R package imputeLCMD^94^. Protein inference was performed with the smallest protein group rule by the function smallestUniqueGroups() with the R package MsQrob2^95^. Protein intensity summarisation was set to “robust” with the R package MsCoreUtils^96^. Protein intensities were normalised using a linear model calculated by the R package MsQrob2. MsQrob2 was used to test pair-wise contrasts and calculate the adjusted *P*-values and fold-change. Proteins with adjusted *P* values ≤ 0.05 and |log_2_(fold-change)| ≥ 2 were defined as significantly regulated. Significantly regulated proteins were selected to generate a heatmap with the R package: pheatmap^97^.

### Infection of THP-1 macrophages

THP-1 cells were differentiated in 8-well µ-Slides (ibidi) at a confluency of 1 × 10^5^ cells per well. Cells were infected with *S. flexneri* strains with a multiplicity of infection (MOI) of 50. Plates were incubated for 30 min under standard conditions before being incubated with fresh RPMI supplemented with 10% hi-FBS and 50 µg/mL gentamycin under standard conditions for 30 min, followed by changing to RPMI supplemented with 10% hi-FBS containing 5 µg/mL gentamycin.

### Immunofluorescence of human cells

Bacteria, coverslips or microslides containing adherent infected or uninfected human cells were washed three times with PBS pH 7.4 and fixed 15 min in 4% paraformaldehyde (in PBS) at room temperature. Fixed cells were washed three times with PBS and subsequently permeabilised 5 min with 0.1% Triton X-100 (in PBS). Cells were then washed three times in PBS and incubated 1 h with primary antibodies diluted in PBS supplemented with 0.1% Triton X-100 and 1% bovine serum albumin (BSA, Sigma-Aldrich, #A9418) for 1 h at room temperature or overnight at 4 °C. Cells were then washed three times in PBS and incubated for 1 h with secondary goat antibodies diluted in PBS supplemented with 0.1% Triton X-100 and 1% BSA, and Alexa Fluor-conjugated phalloidin where indicated. For LAMP1 staining, Saponin 0.05% was used instead of Triton X-100. Stained bacteria cultures and coverslips were washed three times with PBS and mounted on glass slides with ProLong™ Gold Antifade Mountant with DAPI stain (Invitrogen, # P36941).

### Detection of biotinylated proteins for fluorescence microscopy in human cells

Fixed HeLa cells infected with GFP-APEX2 coated *S. flexneri* were permeabilised for 5 min with 0.1% Triton X-100 (in PBS). Endogenous biotin was blocked with 0.05% avidin (Sigma-Aldrich, #A2667) in PBS for 15 min followed by 3 washes in PBS, and then 0.05% biotin (Sigma-Aldrich, #B4501) in PBS for 15 min followed by 3 washes in PBS. Then, proximity biotinylation was performed as described above. Biotinylated proteins were detected using Alexa Fluor 488-conjugated streptavidin, and samples were further processed for immunofluorescence with anti-GFP antibodies to increase the detection of GFP-APEX2.

### Digitonin permeabilisation assay

Selective permeabilisation of the plasma membrane with digitonin for antibody labelling of cytosolic bacteria was performed as previously described^44^. Infected HeLa cells were washed three times with KHM buffer (110 mM potassium acetate, 20 mM HEPES, 2 mM MgCl₂, pH 7.3, prewarmed to 37 °C). Cells were then incubated with 20 μg/mL digitonin in KHM buffer for 1 min at room temperature. Digitonin was removed by washing cells twice with KHM buffer prewarmed to 37 °C. Primary antibodies against *Shigella* group antigen (1:100) diluted in KHM buffer supplemented with 3% BSA were added and incubated for 15 min at 37 °C. Cells were subsequently washed twice with PBS and fixed with 4% paraformaldehyde in PBS for 15 min at room temperature. Secondary antibody labelling was then performed as described for immunofluorescence staining. *S. flexneri* showing colocalization with anti-*Shigella* group antigen antibody was scored as cytosolic by microscopy. As a control, HeLa cells were permeabilised with digitonin and labelled with an antibody against protein disulphide isomerase (1:200) in parallel to verify that endomembranes remained intact.

### Detection of de novo DNA synthesis

To label de novo DNA synthesis, Click-iT EdU Cell Proliferation Kit for Imaging (#C10337 Thermo Fisher Scientific) was used, as previously described^98^. Briefly, 10 μM EdU was added to infected HeLa cells for 1 h. Cells were fixed for immunofluorescence, and click azide/alkyne reactions were performed after permeabilization and prior to antibody staining. A custom ImageJ macro was used to quantify Click-iT EdU signal intensity (see Code availability section). Briefly, bacterial masks were generated from the DAPI channel using manual thresholding to define regions of interest for fluorescence quantification. Mean Click-iT EdU signal intensities were measured within these regions.

### Ethics statements

Animal experiments were performed according to the Animals (Scientific Procedures) Act 1986 and approved by the Home Office (PPL PP5900632).

### Zebrafish husbandry

Adult zebrafish were housed in the Biological Services Facility at LSHTM. WT AB embryos and transgenic *Tg(lyzC:DsRed2)*^*nz50*^ and *Tg(mpx:GFP)*^*i114*^ embryos (for the expression of DsRed2 and GFP in neutrophils, respectively)^99,100^ were obtained by natural spawning, and larvae were maintained at 28.5 °C in E3 media (5 mM NaCl, 0.17 mM KCl, 0.33 mM CaCl_2_, 0.33 mM MgSO_4_). For injections, larvae were anesthetised with tricaine (160 µg/mL, Sigma-Aldrich, #A5040).

### CRISPR Cas9-mediated knockdown in zebrafish larvae

Embryos were injected at the one- to two-cell stage with 1 nL of CRISPR mixture containing 1 µg/µL of gene-specific Guide (g) RNA and 500 µg/mL Cas9 (spCas9 V3, IDT, #1081058) (Supplementary Table 4). For double knockdowns to deplete myeloid cells alongside *ufl1*, the CRISPR mixture contained 1 μg/μl of each gRNA (*spi1b* and *ufl1*) and 500 µg/mL Cas9. gRNA was synthesised as previously described^101^. Briefly, primers containing gene-specific guide sites and a T7 promotor were annealed to a standard scaffold primer and purified (QIAquick PCR Purification Kit, Qiagen, #28104). DNA templates were in vitro transcribed (HiScribe T7 High Yield RNA Synthesis Kit NEB, #E2040S) and purified (GeneJET RNA Cleanup and Concentration Micro Kit, ThermoFisher, #K0841).

### Gene expression analysis

At 1 day post fertilisation (d.p.f.), groups of 10 embryos were lysed and homogenised using a 27-gauge needle in Buffer RLT and RNA extracted with the RNeasy Mini kit (Qiagen, #74104). RNA was reverse transcribed using the QuantiTect Reverse Transcription kit (Qiagen, #205311), and quantitative (q) PCR was performed on a QuantStudio Real-Time PCR system with SYBR green master mix (Applied Biosystems, #10187094). Samples were run in duplicates, and expression normalised to *ef1a1l*. Data was analysed using the 2^−ΔΔ^ Ct method. qPCR primers used were synthesised by Sigma and are listed in Supplementary Table 4.

### Zebrafish injections

To prepare the bacterial inocula, subcultures of either *S. flexneri* or *S*. Typhimurium at OD (600 nm) 0.6 were centrifuged at 4000 x *g* for 4 min at room temperature and washed in PBS. The bacterial pellet was then resuspended in the appropriate volume of injection buffer (4% polyvinyl-pyrrolidone [Sigma-Aldrich, #PVP40] in PBS and 0.5% phenol red [Sigma-Aldrich, #114537] to obtain an infection dose of 10,000 CFU/nL for *S. flexneri* or 1,500 CFU for *S*. Typhimurium. For injection, 1 nL of bacterial suspension was microinjected into either the tail musculature at 3 d.p.f. (for immunofluorescence experiments), The tail fin at 5 d.p.f. (for immunofluorescence experiments), or caudal vein at 3 d.p.f. (for survival assays).

### Wholemount zebrafish immunostaining

Zebrafish larvae were fixed at 2 h.p.i. in 4% paraformaldehyde with 0.4% triton X-100 and incubated at 4 °C overnight. Larvae were then washed three times for five minutes in 0.4% Triton X-100 in PBS, followed by one 20-min wash in 1% Triton X-100 in PBS. Samples were then blocked in blocking buffer (10% hi-FBS, 1% DMSO, 0.1% Tween-20 in PBS) for one hour at room temperature. Primary antibodies were prepared in blocking buffer, and larvae were incubated overnight at 4 °C with gentle rocking. Larvae were washed four times for 15 min with 0.1% Tween-20 in PBS, before incubation overnight at 4 °C with secondary antibodies prepared in blocking buffer with gentle rocking. Larvae were washed as described and then cleared in a glycerol series of 25, 50 and 70%, allowing the larvae to sink to the bottom of the tube between each glycerol concentration. Larvae were mounted on 35-mm glass-bottom dishes (#P35G-1.5-14-C; MatTek) in a minimal volume of 70% glycerol for subsequent imaging.

### Detection of biotinylated proteins for fluorescence microscopy in zebrafish larvae

Fixed zebrafish larvae infected with GFP-APEX2-coated *S. flexneri* were permeabilised with 0.4% triton X-100 and incubated at 4 °C overnight. Larvae were then washed three times for five minutes in 0.4% Triton X-100 in PBS, followed by one 20-min wash in 1% Triton X-100 in PBS. Endogenous biotin was blocked with 0.05% avidin in blocking buffer (10% hi-FBS, 1% DMSO, 0.1% Tween-20 in PBS) for one hour at room temperature, washed four times for 15 min, incubated with 0.05% biotin in blocking buffer for 1 h at room temperature and washed four times for 15 min. Then, proximity biotinylation was performed as described above. Biotinylated proteins were detected using Alexa Fluor 488-conjugated streptavidin, and samples were further processed for immunofluorescence with anti-GFP antibodies to increase the detection of GFP-APEX2.

### Bacterial recovery from zebrafish larvae

At the required timepoint, 5 larvae per group were collected for CFU recovery. Larvae were euthanised by anaesthetic overdose (tricaine) and individually homogenised with a 27-gauge needle in 150 µL of PBS. Homogenates were serially diluted and plated on TSA plates supplemented with 0.01% Congo red or LB agar plates. Plates were incubated for 24 h at 37 °C and CFU enumerated.

### Flow cytometry

5 × 10^4^ individual bacterial cells were analysed using flow cytometry with an LSRII flow cytometer (BD Biosciences). The data was analysed using FlowJo software, version 10.7.1. The bacterial fluorescence was normalised to the median fluorescence of the control condition (no addition of GFP-APEX2) for each biological replicate. The normalised values, median and interquartile range was plotted.

### Secretion assay

Secretion of T3SS effectors was performed as previously described^102^. Briefly, bacteria were grown overnight, subcultured and grown until OD (600 nm) of 0.4–0.5 at 37 °C. Cultures were then incubated for 3 h in the presence or absence of Congo red to induce Type 3 secretion. Secreted proteins were collected from culture supernatants, precipitated using trichloroacetic acid (Sigma-Aldrich, #T0699), and then analysed using SDS-PAGE and Coomassie Brilliant Blue R-250 (Bio-Rad, #1610436) staining.

### SDS-PAGE

Cells were lysed in ice-cold RIPA buffer and then incubated 1:1 with Laemmli buffer (2x) containing 10% (v/v) *β*-mercaptoethanol at 95 °C for 10 min. Proteins were resolved in 12% or 4–20% Mini-PROTEAN TGX precast protein gels (Biorad, #4561046EDU, #4561096EDU).

### Western blotting

Protein gels were transferred to 0.2 μm polyvinylidene difluoride membranes (PVDF, Biorad, #1704156). PVDF membranes were incubated in blocking buffer (TBST buffer containing 3% of dried skimmed milk) for 1 h. PVDF membranes were incubated with the primary antibodies diluted in blocking buffer for 1 h at room temperature or overnight at 4 °C. PVDF membranes were washed 3 × 5–7 min in PBS at room temperature and incubated with secondary goat HRP-conjugated antibodies in blocking buffer for 1 h at room temperature. Milk was replaced by 3% BSA in the blocking buffer for the detection of biotinylated proteins with Streptavidin-HRP or the detection of UFM1 with antibodies. Membranes were developed using Pierce^TM^ ECL plus western blotting substrate (Thermo Scientific, #32132).

### Microscopy

Fluorescence microscopy was performed using a 63×/1.4 C-Plan Apo oil immersion lens or a 40x/1.2 C-Apo water immersion lens on a Zeiss LSM 880 confocal microscope driven by ZEN Black software (v2.3). Microscopy images were obtained using z-stack image series, taking 8–16 slices (HeLa cells) or 40–120 slices (zebrafish larvae). When using the airyscan acquisition mode, confocal images were processed using airyscan processing (Weiner filter) using “Auto Filter” and “3D Processing” options. Alternatively, and for the purpose of quantification of host factors to bacteria, fluorescence microscopy of infected HeLa cells was performed on a Zeiss AxioObserver Z1 fluorescence microscope driven by ZEN Blue v2.3 software, or a Zeiss AxioImager M2 with Apotome 2 driven by ZEN Blue v2.6 software. Whole larvae images were acquired using a stereo fluorescent microscope, Leica M205FA (Leica, Germany). Image files were processed using the ImageJ/Fiji software for representation.

### Quantification of bacterial fluorescence loss

A custom ImageJ macro was used to quantify mCherry fluorescence and UFL1 recruitment to *S. flexneri* (see Code availability section). Briefly, bacterial masks were generated from the DAPI channel using manual thresholding and enlarged by 2 pixels to define regions of interest for fluorescence quantification. Mean mCherry and UFL1 fluorescence intensities were measured within these regions. Bacteria were classified as UFL1-positive or UFL1-negative using a fixed intensity threshold of 5000 (arbitrary units) applied to the UFL1 channel.

### Quantification and statistical analysis

Image processing and quantifications was performed using Fiji. Flow cytometry data was analysed using FlowJo (v.10). Statistical analysis was performed in GraphPad Prism (v.11). Figures were designed using Adobe Illustrator (v.30).

### Reporting summary

Further information on research design is available in the Nature Portfolio Reporting Summary linked to this article.

## Supplementary information

Supplementary Information

Description of Additional Supplementary Files

Supplementary Data 1

Supplementary Data 2

Supplementary Data 3

Supplementary Movie 1

Reporting Summary

Transparent Peer Review file

## Source data

Source Data

## Data Availability

All data are included in the manuscript. The mass spectrometry proteomics data have been deposited in the ProteomeXchange Consortium via the PRIDE^103^ partner repository. The proximity biotinylation dataset is available under accession code PXD063609, and the bacteria recovery dataset under PXD075006. All quantitative data and uncropped gels and blots are provided in the Source Data file and Supplementary Information. Source data are provided with this paper.

## Code availability

ImageJ macros used have been deposited on GitHub and archived on Zenodo. The macro to quantify Click-iT EdU labelling in functionalised *S. flexneri* (release v.1.0.0) is available under DOI 10.5281/zenodo.21964848 [https://doi.org/10.5281/zenodo.21964848]. The macro to measure *S. flexneri* mCherry fluorescence loss upon UFL1 recruitment (release v.1.0.0) is available under DOI 10.5281/zenodo.21964858 [https://doi.org/10.5281/zenodo.21964858].
