# Supplementary material for: Proximity biotinylation at the host-Shigella interface reveals UFMylation as an antibacterial pathway
Source: Nat Commun. 2026 Sep 17;17:10930. doi: 10.1038/s41467-026-77860-y (PMC13680623; doi:10.1038/s41467-026-77860-y)

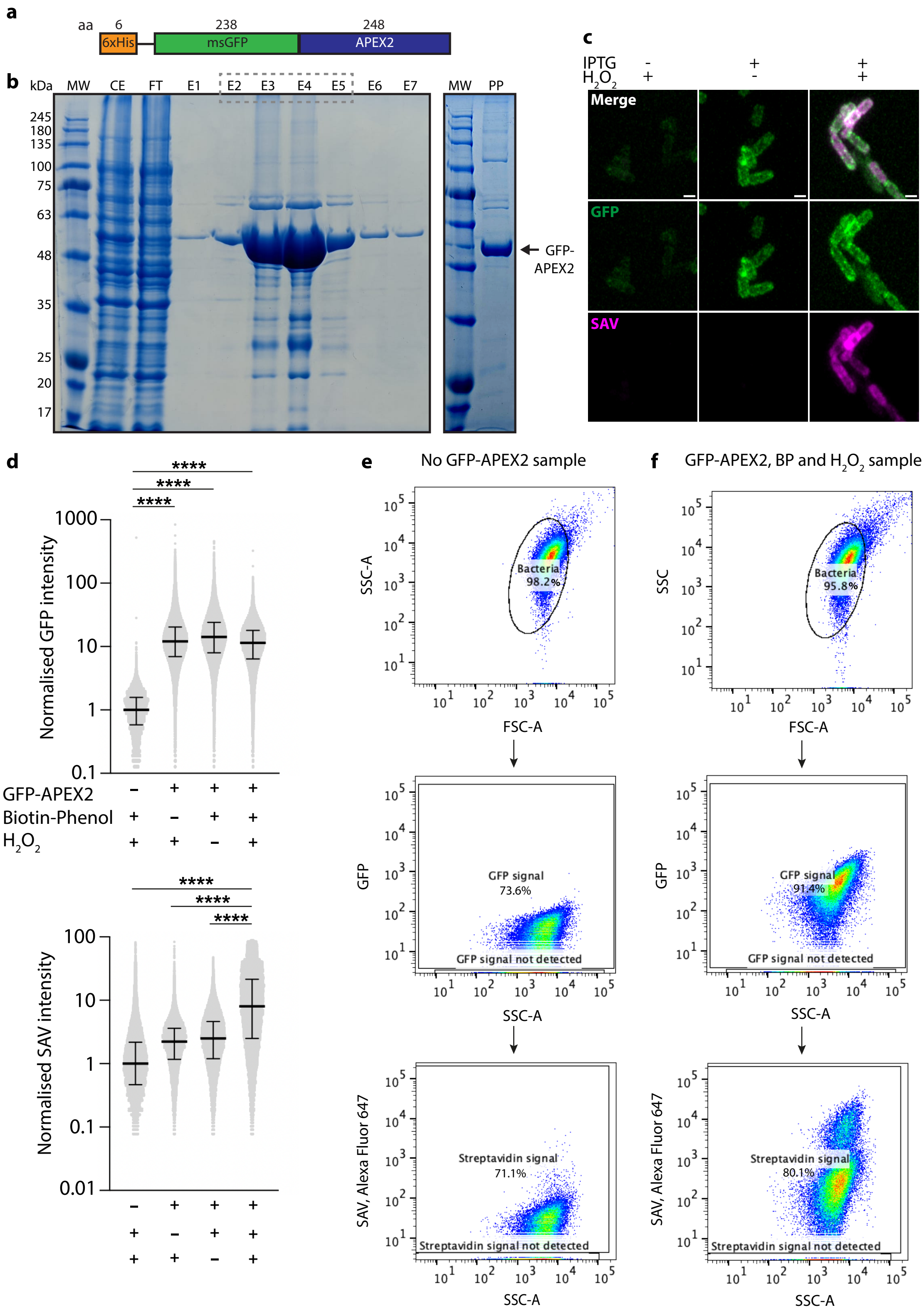

Supplementary Fig. 1. Functionalisation of *Shigella* with GFP-APEX2 and proximity biotinylation of bacterial surface in vitro. a, Diagram representing GFP-APEX2 construct for *E. coli* expression and subsequent purification. **b,** Purification of GFP-APEX2. Coomassie stained SDS-PAGE. Molecular weight marker (MW), clarified extract (CE), flowthrough (FT), elution fractions (E) and purified protein (PP). Fractions E2 to E5 pulled together. Resulting GFP-APEX2 protein has predicted molecular weight 55.69 kDa. **c,** Representative Airyscan confocal images showing biotinylation in vicinity of functionalised *Shigella* in vitro, 2 independent experiments. First, expression of anti-GFP nanobody on *Shigella* surface induced with IPTG, then bacteria coated with GFP-APEX2 and subjected to proximity biotinylation in presence or absence of H2O2. GFP-APEX2 detected using anti-GFP antibody. SAV stands for Alexa Fluor 647-conjugated streptavidin. Scale bar, 1 µm. **d,** Quantification of GFP-APEX2 coating and protein biotinylation at surface of *Shigella* in presence or absence of GFP-APEX2, BP and H2O2, measured by flow cytometry, as shown in c. More than 60,000 bacteria from total of 3 independent experiments analysed per condition. Results represented as median ± interquartile range. Kruskal-Wallis test and Dunn's test, two-sided. **e-f,** Gating strategy used to quantify GFP and Alexa Fluor 647-streptavidin fluorescence in individual bacteria using flow cytometry. Source data are provided as a Source Data file.

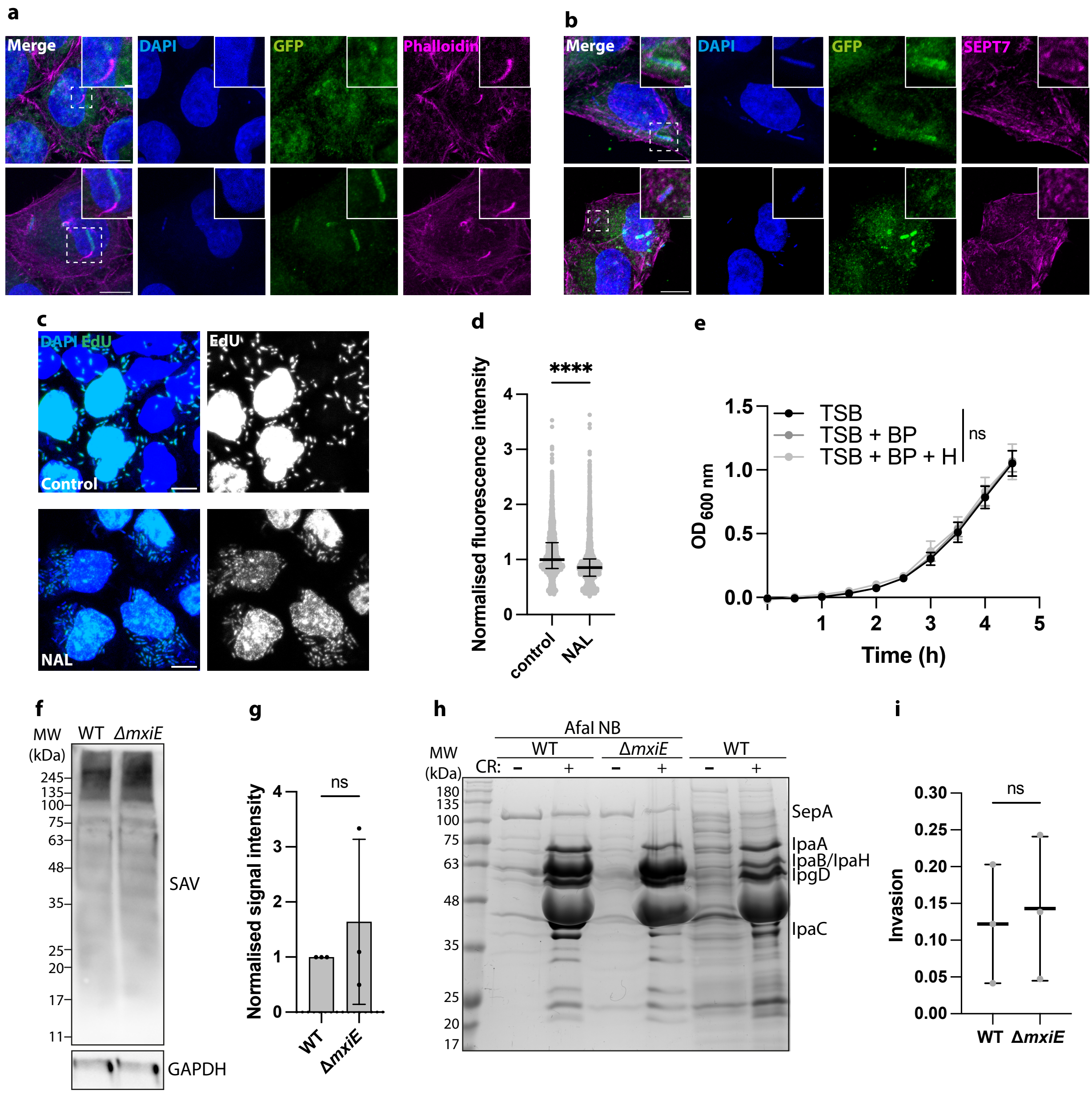

Supplementary Fig. 2. Functionalised *Shigella* infects HeLa cells. a-b, Representative confocal microscopy images of HeLa cells infected with GFP-APEX2 coated *Shigella* WT forming actin tails and entrapped in septin cages, respectively, at 1.5 h.p.i, from 1 biological experiment. **c,** Confocal microscopy images of HeLa cells infected with GFP-APEX2 coated *Shigella* WT and labelled using Click-iT EdU to reveal de novo DNA synthesis, upon DNA synthesis inhibition with nalidixic acid (NAL) or control, at 1.5 h.p.i. Representative images from 3 independent experiments. **d,** Quantified bacterial fluorescence as shown in c (control, n=2,215 bacteria; nalidixic acid, n=3,204; from 3 independent experiments). **e,** Growth of functionalised *Shigella* with GFP-APEX2 in broth (TSB), in presence of biotin phenol (BP) or biotin phenol and hemin-Cl (H), from 3 independent experiments. **f,** Western blot showing smear of biotinylated proteins from HeLa cells infected with WT or Δ*mxiE* functionalised *Shigella* at 1.5 h.p.i. Representative blot of 3 independent experiments. **g,** Quantification of intensity of biotinylated proteins as shown in f, from 3 independent experiments. **h,** In vitro secretion assay of *Shigella* WT and Δ*mxiE* displaying surface nanobodies (NB) and producing AfaI adhesin indicate a functional T3SS upon Congo red (CR) stimulation, from 1 biological experiment. **i,** *Shigella* WT and Δ*mxiE* displaying surface nanobodies and producing AfaI adhesin have similar HeLa invasion rates, 3 independent experiments. Invasion calculated as ratio of CFUs recovered at 1 h.p.i. (internalised bacteria) to CFUs of inoculum. Results are represented as mean ± SD for all panels except for panel d where median and interquartile range are shown. **d,** Mann-Whitney U test. **e,** Two-way ANOVA. **g, i,** Two-tailed Student's t-test. n.s: non-significant, *: p-value ≤ 0.05, **: p-value ≤ 0.01, ***: p-value ≤ 0.001, ****: p-value ≤ 0.0001. Source data are provided as a Source Data file.

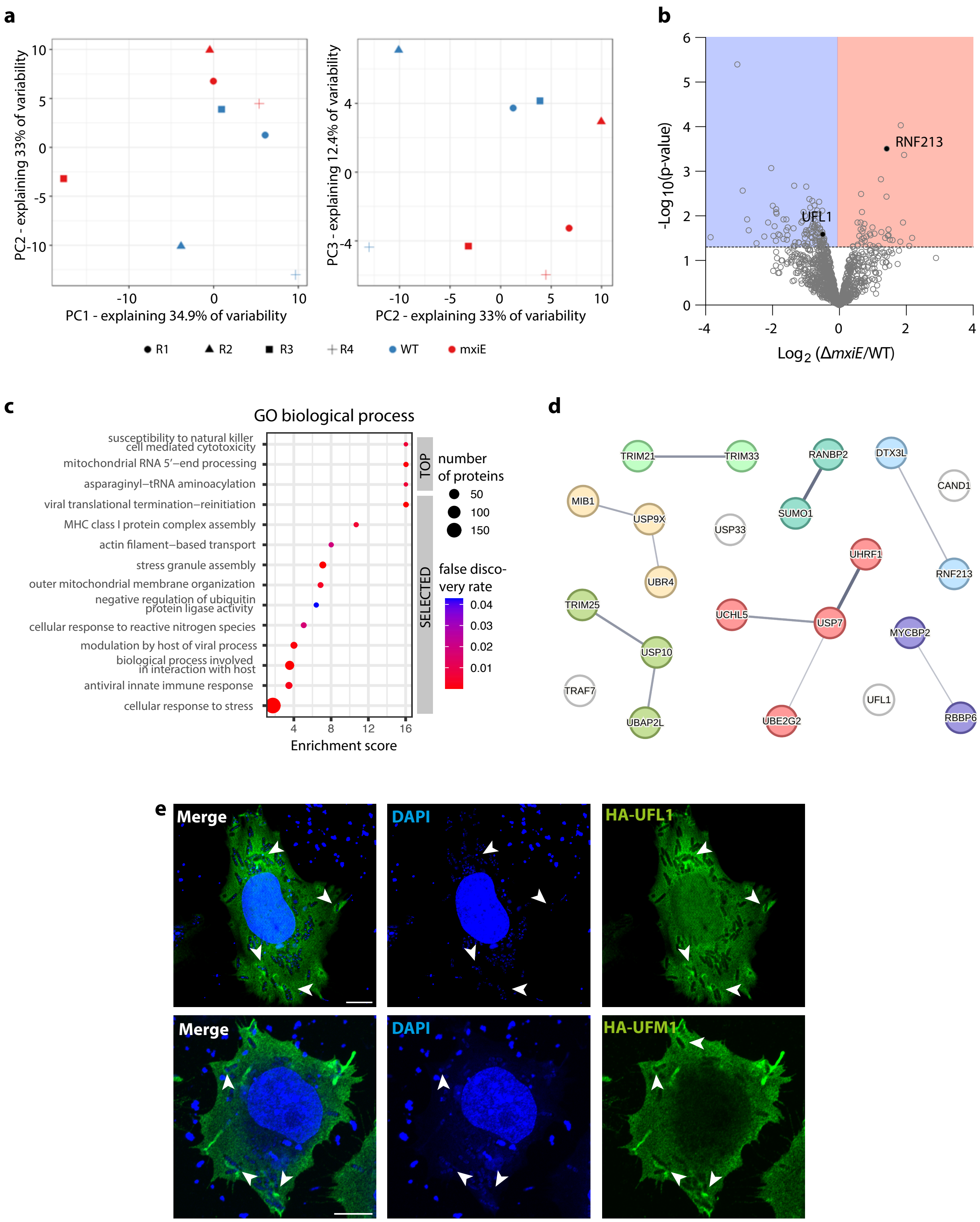

Supplementary Fig. 3. *Shigella* proximity interactome leads to identification of novel host factors recruited to bacteria. a, Principal component analysis showing different proximity interactomes identified during infection of *Shigella* WT and Δ*mxiE*. **b,** Volcano plot of proteins identified, 153 proteins differentially quantified with p-value ≤ 0.05. Blue, enriched in *Shigella* WT samples; red, enriched in *Shigella* Δ*mxiE* samples. **c,** GO terms for biological process enriched in proximity interactome of *S. flexneri* during infection. **d,** STRING diagram displaying E3 ligases and deubiquitylases identified. Edges represent shared physical complex; strength of edge represents confidence. **e,** Representative confocal images of HeLa cells expressing HA-UFL1 or HA-UFM1-dCs and infected with *Shigella* WT expressing AfaI at 3 h.p.i., from 1 biological experiment. Arrows show localisation of UFL1 and UFM1 to intracellular bacteria. Scale bar, 10 μm.

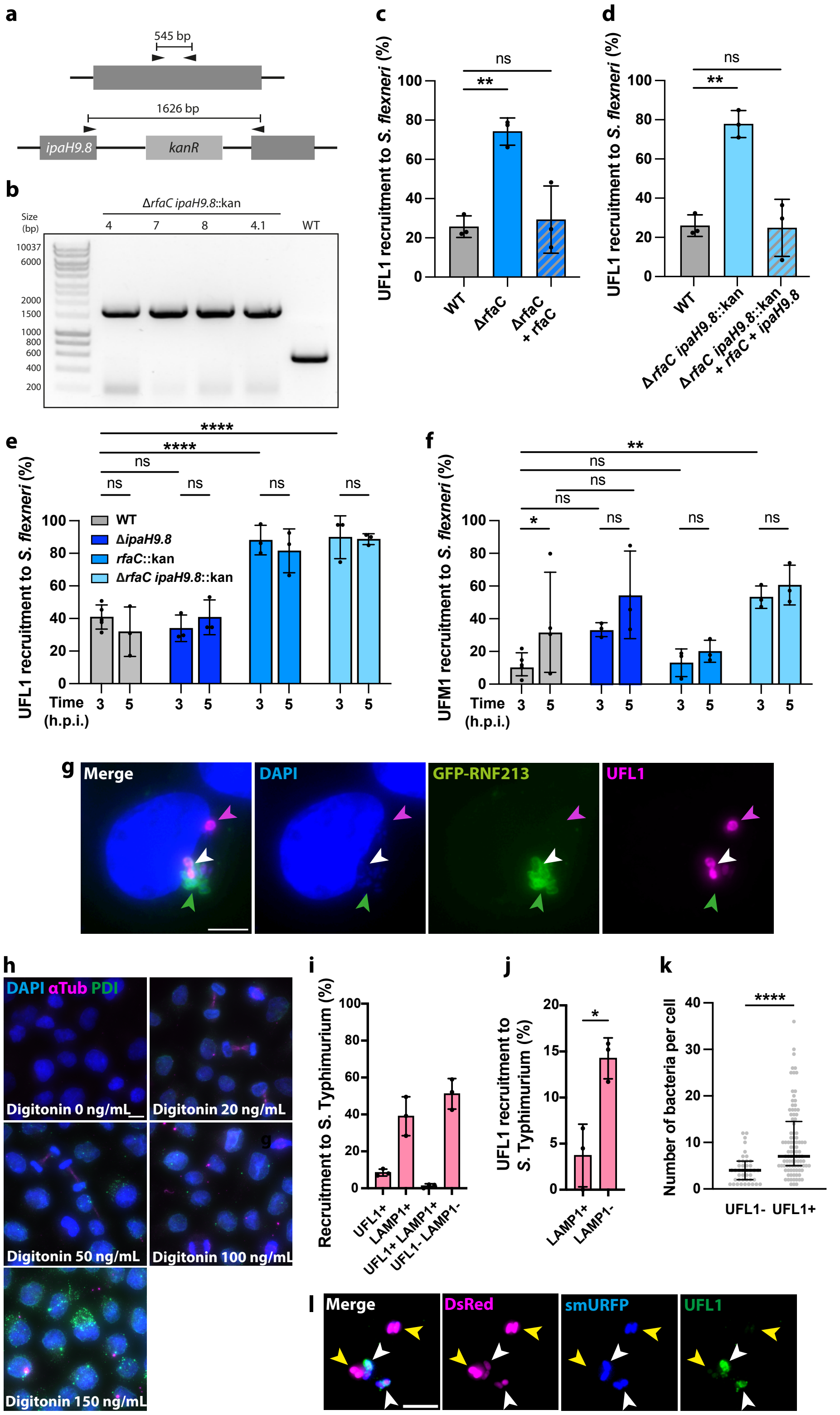

Supplementary Fig. 4. Deletion of *ipaH9.8* and UFL1 and UFM1 recruitment. a, Kanamycin cassette insertion into *ipaH9.8*. Primer annealing prior (top) and after (bottom) insertion. **b,** Cassette insertion by PCR. **c-d**, UFL1 recruitment in *Shigella* WT (n=490), Δ*rfaC* (n=619), Δ*rfaC ipaH9.8*::kan (n=481) and complemented *Shigella* Δ*rfaC* + *rfaC* (n=538), Δ*rfaC ipaH9.8*::kan + *rfaC* + ipaH9.8 (n=675) at 3 h.p.i., 3 independent experiments. **e, f,** Percentage of UFL1- and UFM1- positive bacteria at 3 h.p.i and 5 h.p.i. mCherry *Shigella* WT (UFL1: n=1311, n=837, UFM1: n=882, n=566), Ruby *Shigella* Δ*ipaH9.8* (UFL1: n=463, n=576, UFM1: n=370, n=531), mCherry *Shigella rfaC*::kan (UFL1: n=630, n=721, UFM1: n=643, n=851) and mCherry *Shigella* Δ*rfa* Δ*ipaH9.8*::kan (UFL1: n=553, n=473, UFM: n=526, n=650) from at least 3 independent experiments. **g,** Representative widefield image of GFP-RNF213-expressing HeLa cell infected with *Salmonella* and immunostained for UFL1 at 3 h.p.i., 3 independent experiments. Green, magenta and white arrows: GFP-RNF213-positive, UFL1-positive, or both, respectively. Scale bar, 5 µm. **h,** Representative images of digitonin-permeabilised HeLa cells, 1 biological replicate. Anti-αTubulin (αTub) shows cytosolic access of antibody, anti-protein disulfide isomerase (PDI) shows endoplasmic reticulum integrity. 20 ng/mL-50 ng/mL experiments were independently repeated 3 times. Scale bar, 10 µm. **i,** Percentage of GFP-expressing *Salmonella* (n=663) colocalising with UFL1 and LAMP1-Scarlet-I at 3 h.p.i., 3 independent experiments. **j,** Percentage of UFL1-positive *Salmonella* (n=663) in *Salmonella*-containing vacuoles (LAMP1-mScarlet-I-positive) or cytosol (LAMP1-mScarlet-I-negative). **k,** Bacterial number per infected HeLa cell at 3 h.p.i., grouped by the presence of UFL1-positive bacteria. UFL1-positive HeLa cells (n=97), UFL1-negative HeLa cells (n=42), 3 independent experiments. **l,** Representative widefield image of HeLa cells infected with *Salmonella* harbouring SINA1.7 plasmid at 3 h.p.i., 3 independent experiments. White arrows: smURFP-positive (cytosolic) and UFL1-positive bacteria. Yellow arrows: smURFP-negative bacteria. Scale bar, 5 µm. **c, d, e, f, h, i,** Mean ± SD. k: Median and interquartile range. **c, d,** One-way ANOVA and Dunnet's test, two-sided. **e, f,** Two-way ANOVA and Tukey's test, two-sided. **j,** Two-sided Student's t-test. **k,** Mann-Whitney U test. n.s: non-significant, *: p-value ≤ 0.05, **: p-value ≤ 0.01, ***: p-value ≤ 0.001, ****: p-value ≤ 0.0001. Source data are provided as a Source Data file.

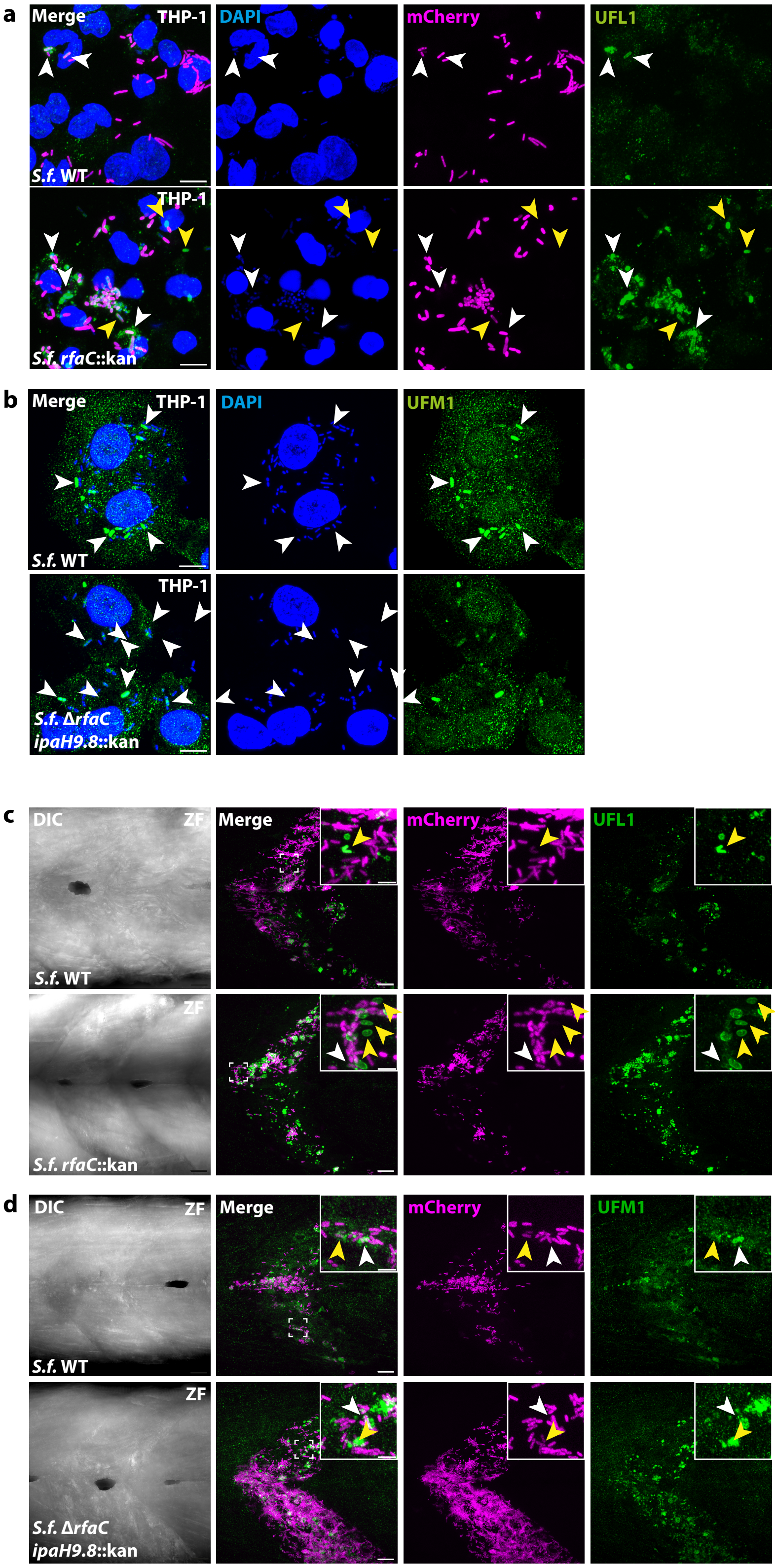

Supplementary Fig. 5. UFL1 and UFM1 recruited to *Shigella* in THP-1 macrophages and zebrafish larvae. a, Representative confocal image of THP-1 macrophages infected with mCherry *Shigella* WT and mCherry *Shigella* rfaC::kan, at 3 h.p.i. from 2 independent experiments. Arrows show localisation of UFL1 to intracellular bacteria. Scale bar, 10 µm. **b,** Representative confocal image of THP-1 macrophages infected with *Shigella* WT and Δ*rfaC ipaH9.8*::kan, at 3 h.p.i. from 1 biological experiment. Arrows show localisation of UFM1 to intracellular bacteria. **c,** Representative confocal image of zebrafish larvae infected at tail musculature with mCherry *Shigella* WT (n=6 larvae) and mCherry *Shigella rfaC*::kan (n=6 larvae) at 2 h.p.i. and immunostained against UFL1, from 2 independent experiments. Scale bar, 20 µm; inset, 5 µm. **d,** Representative confocal image of zebrafish larvae infected at tail musculature with mCherry *Shigella* WT (n=8 larvae) and mCherry *Shigella* ΔrfaC *ipaH9.8*::kan (n=7 larvae) at 2 h.p.i. and immunostained against UFM1, from 2 independent experiments. Scale bar, 20 µm; inset, 5 µm. Arrows show localisation of UFL1 or UFM1 to bacteria. Yellow arrows show bacteria that partially or completely lost mCherry fluorescence. Source data are provided as a Source Data file.

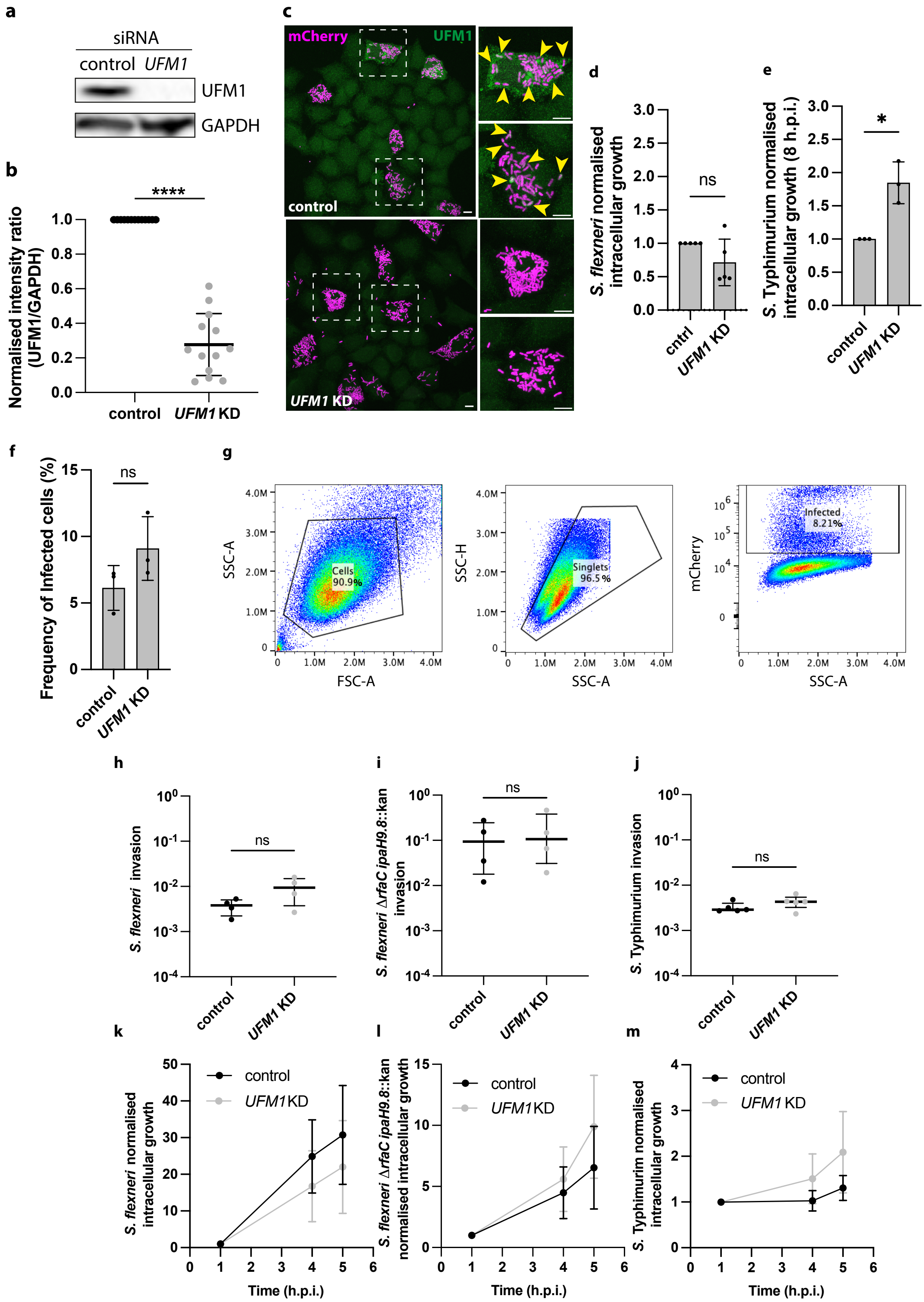

Supplementary Fig. 6. Knockdown of *UFM1* in HeLa cells. a, Representative western blot showing reduced *UFM1* levels after siRNA depletion in HeLa cells, using anti-UFM1 antibodies (Proteintech). **b,** Quantification of UFM1 upon siRNA depletion detected by western blot, from 13 independent experiments. **c,** Representative microscopy images of HeLa cells infected with mCherry *Shigella ΔrfaC ipaH9.8*::kan immunostained against UFM1 at 3 h.p.i., under control conditions or *UFM1* siRNA depletion, confirming antibody specificity, from 1 biological experiment. Yellow arrows show colocalization of UFM1 with *Shigella ΔrfaC ipaH9.8*::kan. **d,** Intracellular growth of *Shigella* WT in *UFM1* depleted HeLa cells normalised to control after 5 h.p.i., from 5 independent experiments. **e,** Intracellular growth of *Salmonella* in *UFM1* depleted HeLa cells normalised to control after 8 h.p.i., from 3 independent experiments. **f,** Percentage of HeLa cells infected with mCherry *Shigella* at 3 h.p.i. after treatment with UFM1 or control siRNA, quantified by flow cytometry. **g,** Gating strategy used to quantify frequency of infected cells according to mCherry *Shigella* fluorescence using flow cytometry. **h-j,** Invasion of *Shigella* WT, *Shigella ΔrfaC ipaH9.8*::kan and *Salmonella* in HeLa cells upon *UFM1* depletion. Invasion calculated as ratio of CFUs recovered at 1 h.p.i. (internalised bacteria) to CFUs of inoculum, from at least 4 independent experiments. **k-m,** Intracellular growth of *Shigella* WT, *Shigella ΔrfaC ipaH9.8*::kan and *Salmonella* in HeLa cells upon UFM1 depletion, normalised to 1 h.p.i., from at least 5 independent experiments. Results are represented as mean ± SD for all panels except h-j where median and interquartile range are shown. **b-j,** Two-sided Student's t-test. n.s: non-significant, *: p-value ≤ 0.05, **: p-value ≤ 0.01, ***: p-value ≤ 0.001, ****: p-value ≤ 0.0001. Source data are provided as a Source Data file.

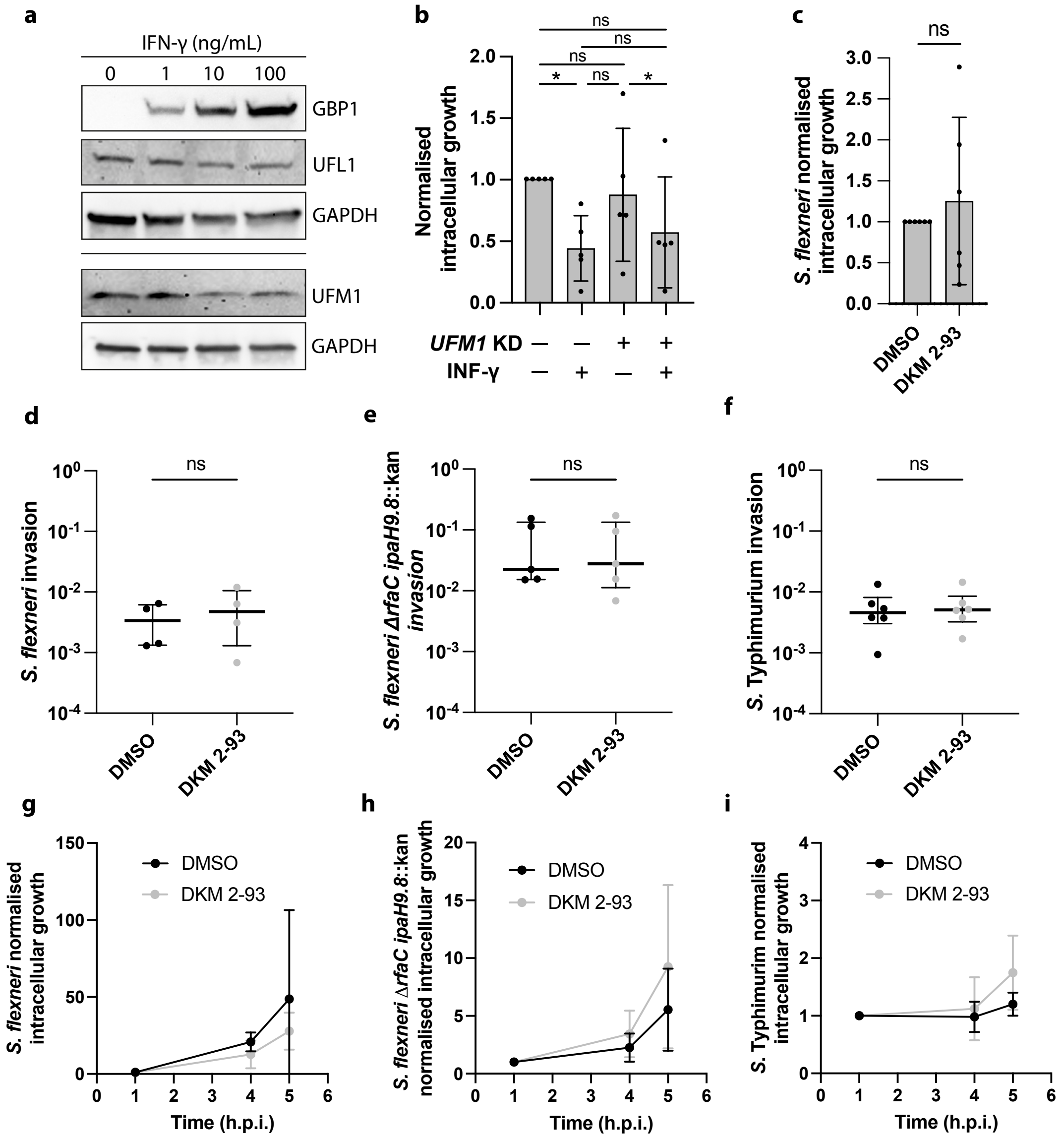

Supplementary Figure 7. Treatment with IFN-γ and DKM 2-93 in HeLa cells. **a,** Western blot of HeLa cell samples treated with increasing concentrations of IFN-γ and immunoblotted against UFL1 and UFM1. GBP1 used as positive control of induction. Representative blot from 2 independent experiments. **b,** Intracellular growth of *Shigella* in HeLa cells treated with *UFM1* siRNA and IFN-γ normalised to control (control siRNA, mock treatment) at 5 h.p.i., from 5 independent experiments. **c,** Intracellular growth of *Shigella* in HeLa cells treated with DKM 2-93 normalised to control at 5 h.p.i., from 5 independent experiments. **d-f,** Invasion of *Shigella* WT, *Shigella* Δ*rfaC ipaH9.8*::kan and *Salmonella* in HeLa cells upon DKM 2-93 treatment, from at least 4 independent experiments. Invasion is calculated as ratio of CFUs recovered at 1 h.p.i. (internalised bacteria) to CFUs of inoculum. **g-i,** Intracellular growth of *Shigella* WT, *Shigella* Δ*rfaC ipaH9.8*::kan and *Salmonella* in HeLa cells upon DKM 2-93 treatment, normalised to 1 h.p.i., from at least 5 independent experiments. Results represented as mean ± SD for all panels except d-f where median and interquartile range are shown. **b,** Repeated Measures one-way ANOVA and Tukey's test, two-sided. **c-f** Two-sided Student's t-test. n.s: non-significant, *: p-value ≤ 0.05, **: p-value ≤ 0.01, ***: p-value ≤ 0.001, ****: p-value ≤ 0.0001. Source data are provided as a Source Data file.

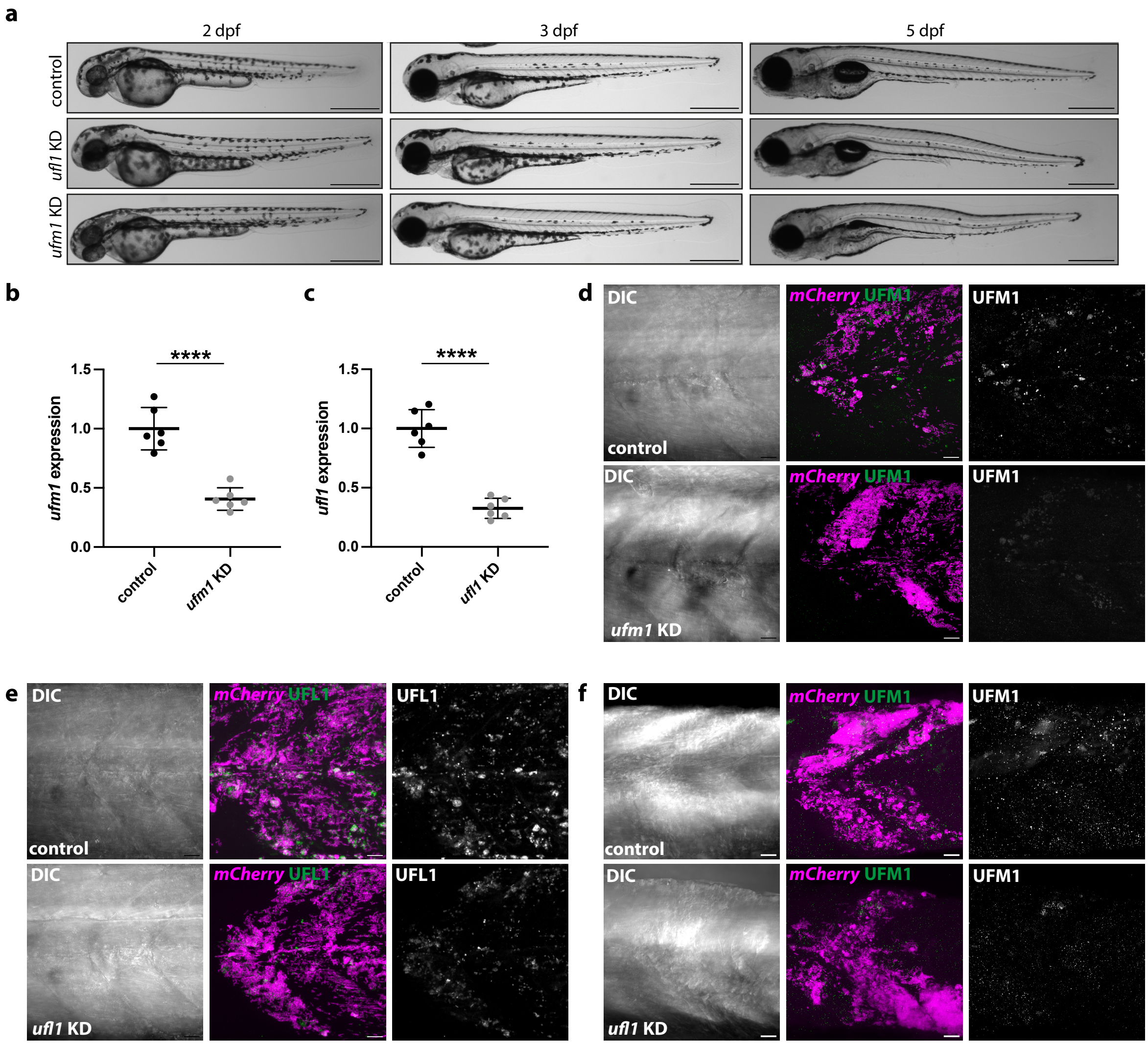

Supplementary Fig. 8. Characterisation of *ufl1* and *ufm1* CRISPR/Cas9 knockdown zebrafish larvae. a, Representative images of CRISPR Cas9 generated zebrafish larvae with scramble, *ufl1* or *ufm1* gRNAs at 2-, 3- and 5 d.p.f. Scalebar, 500 µm. **b-c,** Quantification of *ufm1* and *ufl1* expression in zebrafish larvae upon CRISPR/Cas9 knockdown by qRT-PCR. n=6 larvae tested per condition. Values normalised to median of control (scramble) condition. Results represented as mean ± SD. Two-sided Student's t-test. **d-f,** Representative microscopy images of zebrafish larvae infected with mCherry *Shigella* at tail musculature at 2 h.p.i. and immunostained against UFM1 and UFL1, upon *ufm1* and *ufl1* depletion or control conditions, confirming specificity of antibodies. At least n=2 larvae analysed per condition. Scale bar, 20 µm. Source data are provided as a Source Data file.

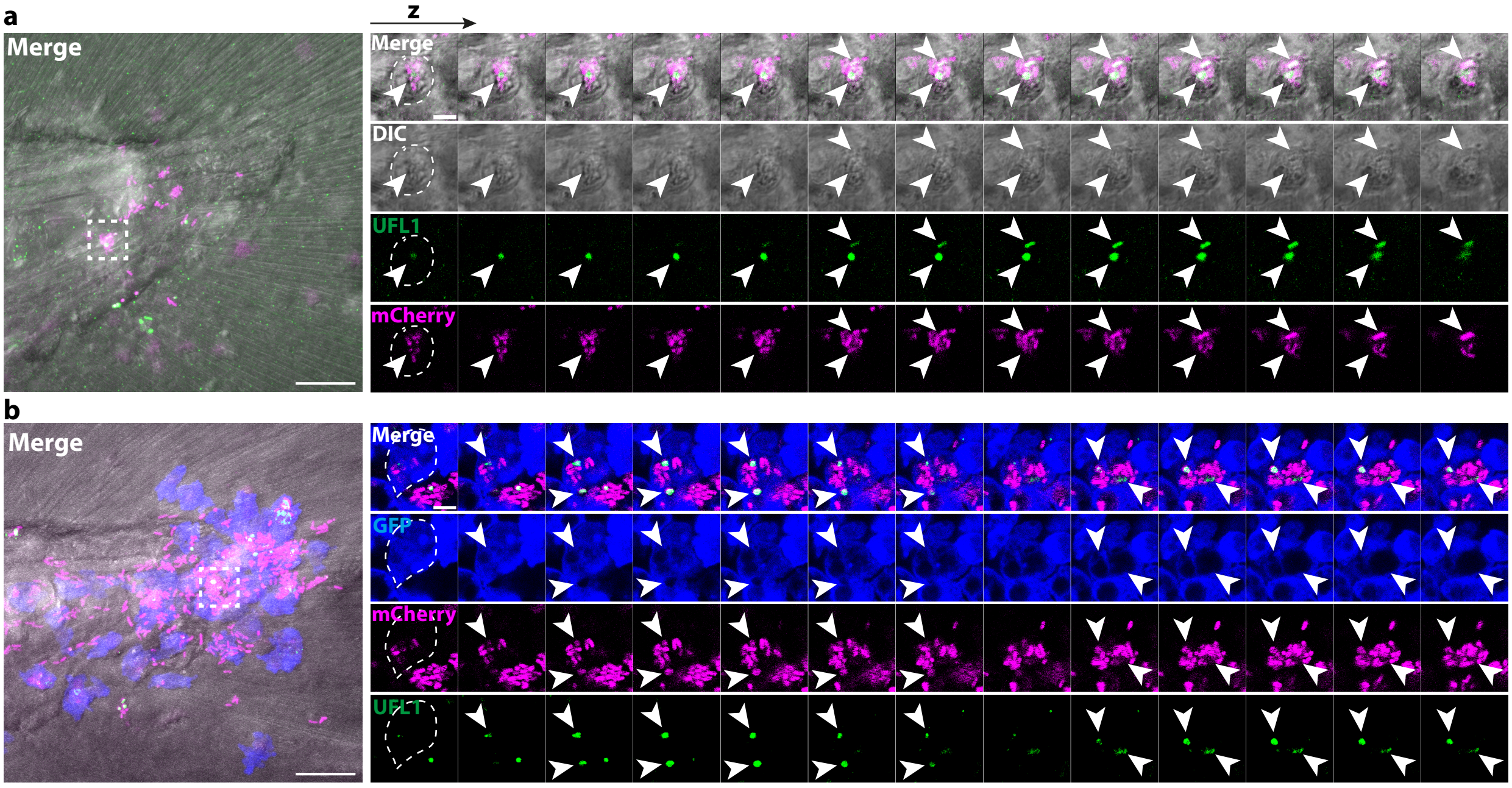

Supplementary Fig. 9. Intracellular recruitment of UFL1 to *S. flexneri* in zebrafish larvae. a-b, Confocal images of zebrafish tail fin infection of *Shigella* showing recruitment of UFL1 to intracellular *Shigella* at 2 h.p.i., representative examples from n=6 larvae. High magnification images represent different z-planes of same z-stack. Zebrafish in b shows fluorescently labelled neutrophils using *Tg(mpx:GFP)*^{i114} larvae. Arrows show localisation of UFL1 to mCherry *Shigella*. Scalebar, 20 µm; inset, 5 µm.

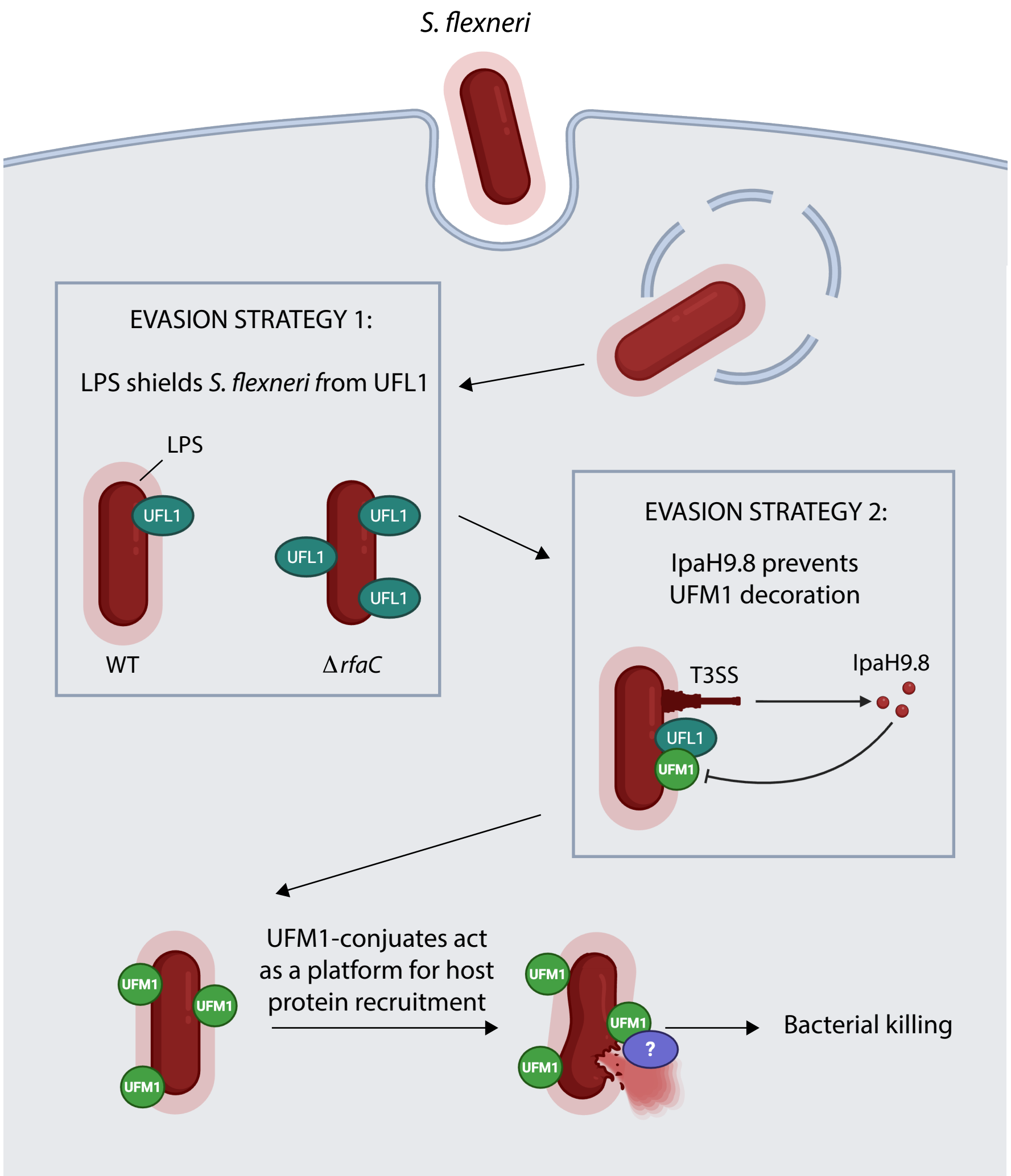

Supplementary Fig. 10. Proposed model for intracellular bacteria UFMylation. After *Shigella* internalisation and escape to the cytosol, UFL1 is recruited to the *Shigella* surface, where it mediates decoration of bacteria with UFM1 to target them for degradation in a mechanism independent of autophagy. UFL1 recruitment is hampered by bacterial LPS, and the Type 3 secreted effector IpaH9.8 binds to UFM1 to prevent *Shigella* decoration. Bacteria associated with UFM1 serve as a platform to recruit downstream UFMylation effectors, which leads to bacterial killing. Created in BioRender. Lopez, A. (2026) https://BioRender.com/py3vl8q.

Frequency	Gene ID	Description
23	ATP1B3	ATPase, Na+/K+ transporting, beta 3 polypeptide
11	ARHGDIB	Rho GDP dissociation inhibitor (GDI) beta
7	KRT222	keratin 222
7	CCT3	chaperonin containing TCP1, subunit 3 (gamma)
6	ATP1B1	ATPase, Na+/K+ transporting, beta 1 polypeptide
6	MRVI1	murine retrovirus integration site 1 homolog
5	PCNA	proliferating cell nuclear antigen
5	ZKSCAN3	zinc finger with KRAB and SCAN domains 3
4	C17orf46	chromosome 17 open reading frame 46
4	PGM1	phosphoglucomutase 1
3	C9orf78	chromosome 9 open reading frame 78
3	GBP4	guanylate binding protein 4
3	EIF1B	eukaryotic translation initiation factor 1B
3	MAP3K1	mitogen-activated protein kinase kinase kinase 1
2	TMED7	transmembrane emp24 protein transport domain containing 7
2	MPHOSPH8	M-phase phosphoprotein 8
2	MYBPC1	myosin binding protein C, slow type
2	RPS20	ribosomal protein S20
2	ZBTB38	zinc finger and BTB domain containing 38
1	LOC284412	hypothetical LOC284412, non-coding RNA
1	PRDX6	peroxiredoxin 6
1	AP1G1	adaptor-related protein complex 1
1	C14orf45	gamma 1 subunit chromosome 14 open reading frame 45
1	COPS5	COP9 constitutive photomorphogenic homolog subunit 5
1	CUL1	cullin 1
1	MRPS9	mitochondrial ribosomal protein S9
1	N4BP2L2	NEDD4 binding protein 2-like 2
1	ZNF350	zinc finger protein 350
1	ANKRD7	ankyrin repeat domain 7
1	FMO2	flavin containing monooxygenase 2 (non-functional)
1	PEAK1	NKF3 kinase family member
1	PNISR	PNN-interacting serine/arginine-rich protein
1	SV2B	synaptic vesicle glycoprotein 2B
1	intergenic	chromosome 3 genomic contig
1	ANAPC1	anaphase promoting complex subunit 1
1	ATP2C1	ATPase, Ca++ transporting, type 2C, member 1
1	CD109	CD109 molecule
1	COMMD8	COMM domain containing 8
1	F13A1	coagulation factor XIII, A1 polypeptide
1	OLFML3	olfactomedin-like 3
1	SSR1	signal sequence receptor, alpha
1	UFM1	ubiquitin-fold modifier 1
1	VDAC2	voltage-dependent anion-selective channel protein
1	WWOX	2 WW domain containing oxidoreductase
1	ZBRK1	zinc-finger protein ZBRK1
1	ZNF177	zinc finger protein 177
1	ZNF237	zinc finger protein 237
1	ZNF251	zinc finger protein 251

Supplementary Table 1. Top 50 mammalian proteins shown to interact with IpaH9.8 by Yeast-Two-Hybrid protein- protein interaction screen, ordered by the number of clones detected.

Bacterial strain	Genotype	Reference
Shigella flexneri srv. 5a str. M90T	Naturally Sm^R	[1]
S. flexneri mCherry	Constitutively producing mCherry, $Carb^R$, Sm^R	[2]
S. flexneri GFP	Constitutively producing GFP, $Carb^R$	[1]
S. flexneri AfaI	Constitutively producing the adhesin AfaI, Amp^R, Sm^R	[1]
S. flexneri pNVgfp AfaI	Displaying an anti-GFP nanobody on the bacterial surface upon induction with IPTG, constitutively producing the adhesin AfaI, Amp^R, Cm^R, Sm^R	This study
S. flexneri Δ*mxiE* Ruby	*mxiE*$^-$, constitutively producing Ruby, $Carb^R$, Sm^R	(Gift from Felix Randow lab)
S. flexneri Δ*mxiE pNVgfp* AfaI	*mxiE*$^-$, displaying an anti-GFP nanobody on the bacterial surface upon induction with IPTG, constitutively producing the adhesin AfaI, Amp^R, Cm^R, Sm^R	This study
S. flexneri Δ*rfaC*	*rfaC*$^-$, Sm^R	[3]
S. flexneri Δ*rfaC ipaH9.8::kan*	*rfaC*$^-$, *ipaH9.8*$^-$, Kan^R, Sm^R	This study
S. flexneri rfaC::kan mCherry	*rfaC*$^-$, constitutively producing mCherry, $Carb^R$, Kan^R, Sm^R	This study
S. flexneri Δ*ipaH9.8* Ruby	*ipaH9.8*$^-$, constitutively producing Ruby, $Carb^R$, Kan^R, Sm^R	(Gift from Felix Randow lab)
S. flexneri Δ*rfaC ipaH9.8*::kan mCherry	*rfaC*$^-$, *ipaH9.8*$^-$, constitutively producing mCherry, $Carb^R$, Kan^R, Sm^R	This study
Escherichia coli DH5a	F– **φ**80lacZΔM15 Δ(lacZYA-argF) U169 *rec*A1 *end*A1 *hsd*R17(r_K^-, m_K^+) *pho*A *sup*E44 **λ**$^-$ *thi*-1 *gyr*A96 *rel*A1	Thermo Fisher Scientific (#18265017)
E. coli BL21 (DE3)	F– *ompT gal dcm lon hsdSB* (*rB*$^-$ *mB*$^-$) pLysS, Cm^R	Thermo Fisher Scientific (#EC0114)
Salmonella enterica subs. *enterica* srv. Typhimurium str. SL1344 pmCherry	For the arabinose inducible expression of mCherry	This study
Salmonella enterica subs. *enterica* srv. Typhimurium str. SL1344 GFP	Constitutively producing GFP, $Carb^R$	[4]

Supplementary Table 2. Bacterial strains used in this study.

Plasmid	Relevant characteristics	Reference
pKD46	*repA101*(ts) *oriR101* bla *ParaB*l-Red recombinase, Carb^R.	[5]
pKD4	oriR$_{\gamma R6k}$ *FRT::kan::FRT*, Kan^R.	[5]
pCP20	For elimination of resistance gene.	[5]
pFPV25.2	For constitutive expression of GFP.	[6]
pFPV-mCherry	For constitutive expression of mCherry.	[7]
pmCherry	Derivative of pFUS-PBAD[8]. For arabinose inducible expression of mCherry.	This study
pAC*afaI*	For constitutive expression of the bacterial adhesin AfaI, ori p15A, Amp^R.	[9]
pNVgfp	For IPTG inducible expression of anti-GFP nanobody on *S. flexneri* surface [Intimin$_{EHEC}$ (1-659)-E-Vgfp-myc tag].	[10]
prfaC	pFUS-PBAD derivative for complementation experiments with rfaC, arabinose inducible. Kan^R.	[3]
pUC19IpaH9.8	For complementation experiments WT IpaH9.8. Amp^R.	This study
pUC19IpaH9.8C337A	For complementation experiments with catalytically dead IpaH9.8. Amp^R.	This study
pTRC-GFP-APEX2	Derivative of pTRC-APEX2[38], msGFP fused the N-terminus of APEX2.	This study
pGEX6P-1 GST-IpaH9	For the purification of GST-IpaH9.8. Amp^R	(Gift from Neal Alto lab)
pGEX6P-1 GST-ubiquitin	For the purification of GST-Ubiquitin. Amp^R	(Gift from David Komander lab)
pETM11 His-GBP1	For the purification of His-GBP1. Kan^R	[11]
pJB179-10	Derivative of pGKBT7 vector. IpaH9.8 C337A bait vector for Yeast-Two-Hybrid. Neo^R.	This study
pLenti-X1-Neo-HA-UFL1	Lentiviral expression of HA-tagged human UFL1. Carb^R.	[11]
pRK5-HA-UFM1-dCS	Mammalian expression of HA-tagged human UFM1-cCS Carb^R.	[11]
pDEST-eGFP-RNF213	For mammalian expression of eGFP-RNF213, Kan^R.	[12]
Lamp1-mScarlet-I	For mammalian expression of mScarlet-I-tagged Lamp1	[13]

Supplementary Table 3. Plasmids used in this study.

Primer	Sequence (5'-3')	Use
del-ipaH9.8-fw	gtaatttcctcactgagctaccagcattttctgagggaaatagtgtaggctggagct gcttc	Deletion of *ipaH9.8*
del-ipaH9.8-rv	accaggagggttttccggagattgttccatgtgagcgcgacacatgggaattagcc atggtcc	Deletion of *ipaH9.8*
comp-ipaH9.8-fw	gtaatttcctcactgagctaccagcattttctgagggaaata	Confirmation of *ipaH9.8* deletion
comp-ipaH9.8-rv	accaggagggttttccggagattgttccatgtgagcgcgac	Confirmation of *ipaH9.8* deletion
msGFP-fw	ccaccaccacatgagtaaaggtgaagaac	Amplification/cloning msGFP into pTRC-APEX2
msGFP-rv	aagactttccaaagtagagttcatccatg	Amplification/cloning msGFP into pTRC-APEX2
pTRC-APEX2-fw	actctacaaaggaaagtcttacccaactgtgag	Amplification/cloning of pTRC-APEX2
pTRC-APEX2-rv	ctttactcatgtggtggtggtggtggtg	Amplification/cloning of pTRC-APEX2
scramble target 1	taatacgactcactataggcaggcaaagaatccctgccgttttagagctagaaatag c	Zebrafish Cas9-mediated KD
scramble target 2	taatacgactcactataggtacagtggacctcggtgtcgttttagagctagaaatag c	Zebrafish Cas9-mediated KD
scramble target 3	taatacgactcactataggcttcatacaatagacgatggttttagagctagaaatagc	Zebrafish Cas9-mediated KD
scramble target 4	taatacgactcactataggtcgttttgcagtaggatcggtttagagctagaaatagc	Zebrafish Cas9-mediated KD
ufl1 target_1	taatacgactcactataggagctcatcacgaatttccgttttagagctagaaatagc	Zebrafish Cas9-mediated KD
ufl1 target_2	taatacgactcactatagggctgggcagaataatccagttttagagctagaaatagc	Zebrafish Cas9-mediated KD
ufl1 target_3	taatacgactcactataggagacgacgcacagctcacgttttagagctagaaatag c	Zebrafish Cas9-mediated KD
ufl1 target_4	taatacgactcactataggttcatacctcgctggtcagttttagagctagaaatagc	Zebrafish Cas9-mediated KD
ufm1 target_1	taatacgactcactataggacgctgacgtcggacccggttttagagctagaaatag c	Zebrafish Cas9-mediated KD
ufm1 target_2	taatacgactcactatagggcagaagaggtgagttgagttttagagctagaaatag c	Zebrafish Cas9-mediated KD
ufm1 target_3	taatacgactcactatagggatggcactggtagctgcgttttagagctagaaatagc	Zebrafish Cas9-mediated KD
ufm1 target_4	taatacgactcactataggcaatccggcgcagacagcgttttagagctagaaatag c	Zebrafish Cas9-mediated KD
spi1b target 1	taatacgactcactatagggcatccgtacagaatggagttttagagctagaaatagc	Zebrafish Cas9-mediated KD
spi1b target 2	taatacgactcactataggcgtgtctagatcactcttgttttagagctagaaatagc	Zebrafish Cas9-mediated KD
spi1b target 3	taatacgactcactataggcttacttgccccggacatgttttagagctagaaatagc	Zebrafish Cas9-mediated KD
spi1b target 4	taatacgactcactatagggcttgaccttctgcgaaagttttagagctagaaatagc	Zebrafish Cas9-mediated KD
sgRNA Scaffold Primer	aaaagcaccgactcggtgccactttttcaagttgataacggactagccttattttaact tgctatttctagctctaaaac	Zebrafish Cas9-mediated KD
ufl1 fw	acgtgcagttcctctcagtg	qRT-PCR
ufl1 rv	acttgtcgacaggaaggctg	qRT-PCR
ufm1 fw	gcggacctctacctttgctt	qRT-PCR
ufm1 rv	acttctcccctttccccagt	qRT-PCR
il6 fw	tcaacttctccagcgtgatg	qRT-PCR
il6 rv	tctttccctcttttcctcctg	qRT-PCR
tnfa fw	agaccttagactggagagatgac	qRT-PCR
tnfa rv	caaagacacctggctgtagac	qRT-PCR
ef1a1l fw	aagcttgaagacaaccccaagagc	qRT-PCR
ef1a1l rv	actcctttaatcactcccaccgca	qRT-PCR

Supplementary Table 4. Primers used in this study.

Supplementary references.

1. Mostowy, S. *et al.* Entrapment of intracytosolic bacteria by septin cage-like structures. *Cell Host Microbe* 8, 433–444 (2010).

2. Mostowy, S. *et al.* The zebrafish as a new model for the in vivo study of *Shigella flexneri* interaction with phagocytes and bacterial autophagy. *PLoS Pathog*. 9, e1003588 (2013

3. Lobato-Márquez, D. *et al.* Mechanistic insight into bacterial entrapment by septin cage reconstitution. *Nat Commun* 12:1 12, 1–14 (2021).

4. Fisch, D. *et al.* Human GBP 1 is a microbe-specific gatekeeper of macrophage apoptosis and pyroptosis. The *EMBO Journal,* 38(13), e100926, (2019).

5. Molina-García, L. & Giraldo, R. Aggregation interplay between variants of the RepA-WH1 prionoid in *Escherichia coli. J Bacteriol* 196, 2536–2542 (2014).

6. Valdivia, R. H. & Falkow, S. Bacterial genetics by flow cytometry: Rapid isolation of *Salmonella* Typhimurium acid-inducible promoters by differential fluorescence induction. *Mol Microbiol* 22, 367–378 (1996).

7. Drecktrah, D. *et al.* Dynamic behavior of *Salmonella*-induced membrane tubules in epithelial cells. *Traffic* 9, 2117–2129 (2008).

8. Lobato-Márquez, D., Moreno-Córdoba, I., Figueroa, V., Diáz-Orejas, R. & Garciá-Del Portillo, F. Distinct type I and type II toxin-antitoxin modules control *Salmonella* lifestyle inside eukaryotic cells. *Sci Rep* 5, 1–10 (2015).

9. López-Jiménez, A. T. *et al.* High-content high-resolution microscopy and deep learning-assisted analysis reveals host and bacterial heterogeneity during *Shigella* infection. *Elife* 13, RP97495 (2025)

10. Salema, V. *et al.* Selection of single domain antibodies from immune libraries displayed on the surface of *E. coli* cells with two β-domains of opposite topologies. *PLoS One* 8, e75126 (2013).

11. Liang, J. R. *et al.* A genome-wide ER-phagy screen highlights key roles of mitochondrial metabolism and ER-resident UFMylation. *Cell* 180, 1160-1177.e20 (2020).

12. Thery, F. *et al.* Ring finger protein 213 assembles into a sensor for ISGylated proteins with antimicrobial activity. *Nat Commun* 12, 5772 (2021).

13. Chertkova, A. *et al.* Robust and bright genetically encoded fluorescent markers for highlighting structures and compartments in mammalian cells. *bioRxiv* 160374 (2020).

Source gels and blots in Supplementary Figures.

Supplementary Fig. 1b

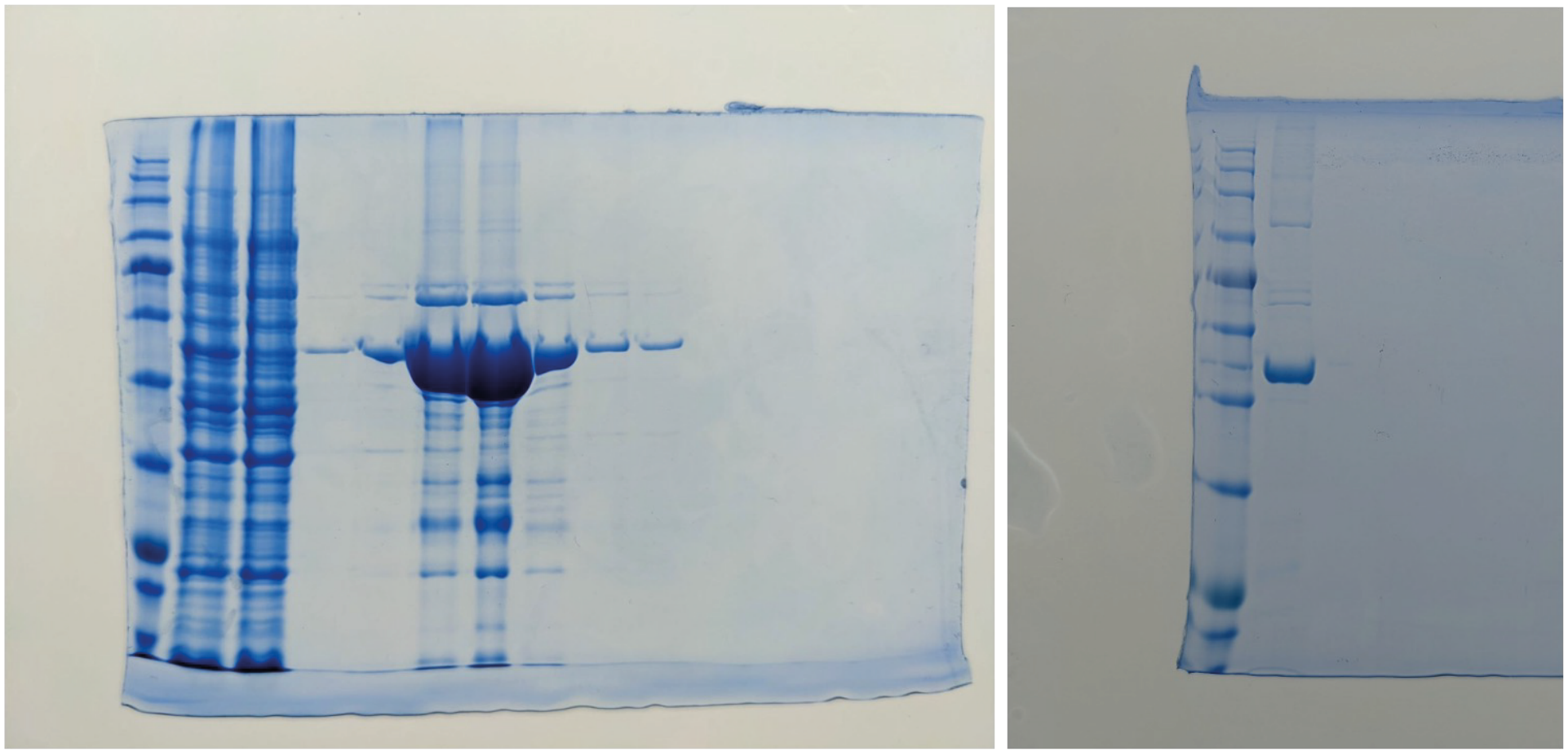

Supplementary Fig. 2f

Streptavidin-HRP

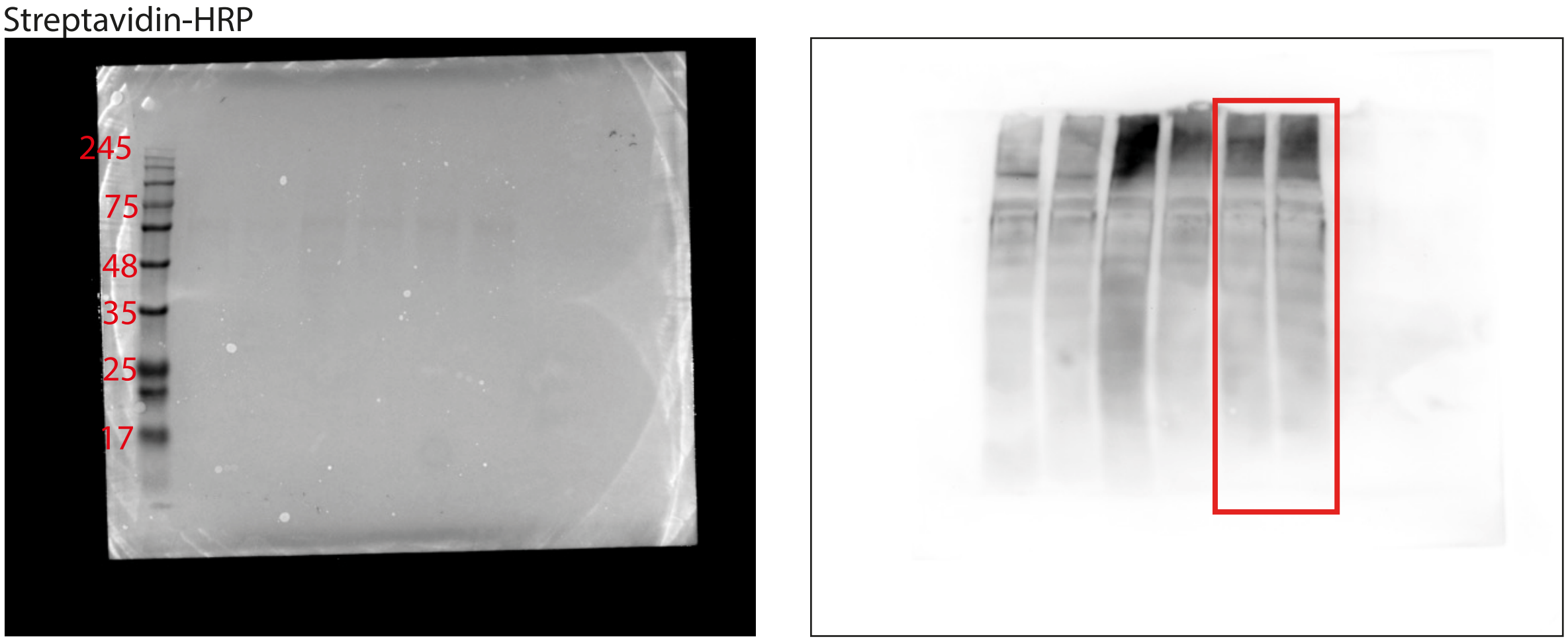

anti-GAPDH

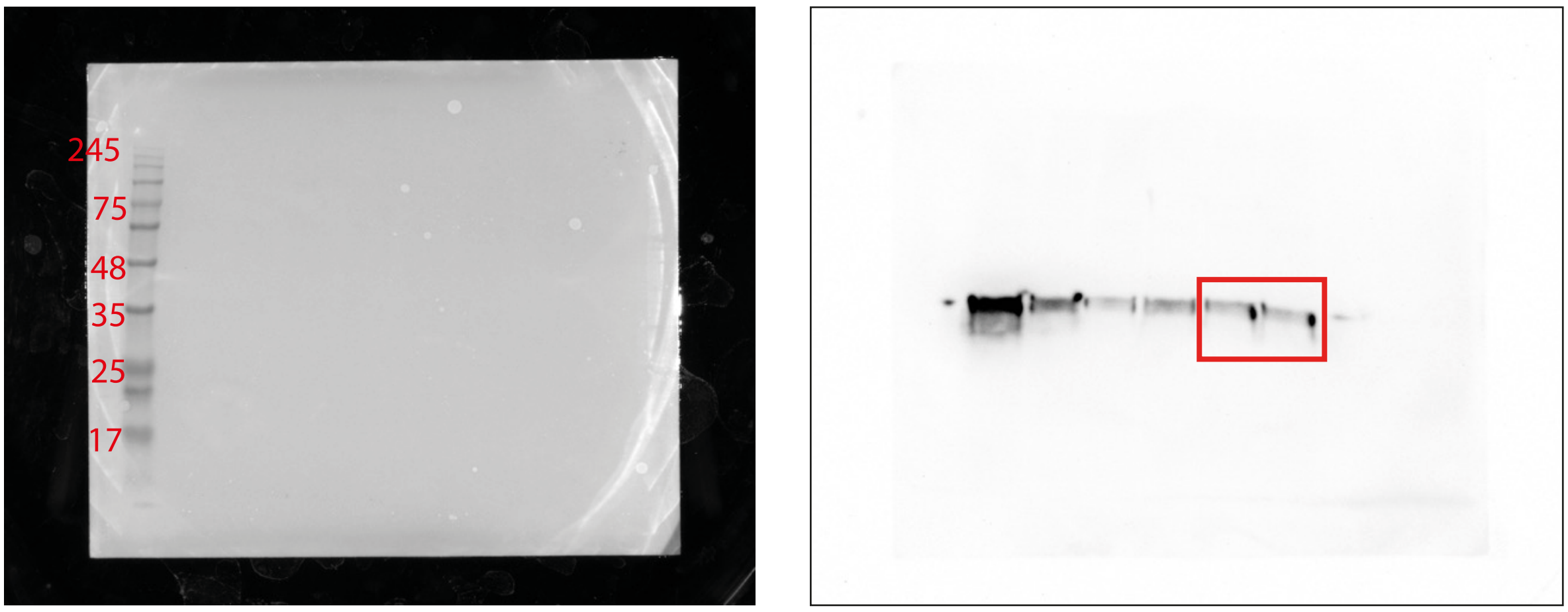

Supplementary Fig. 2h

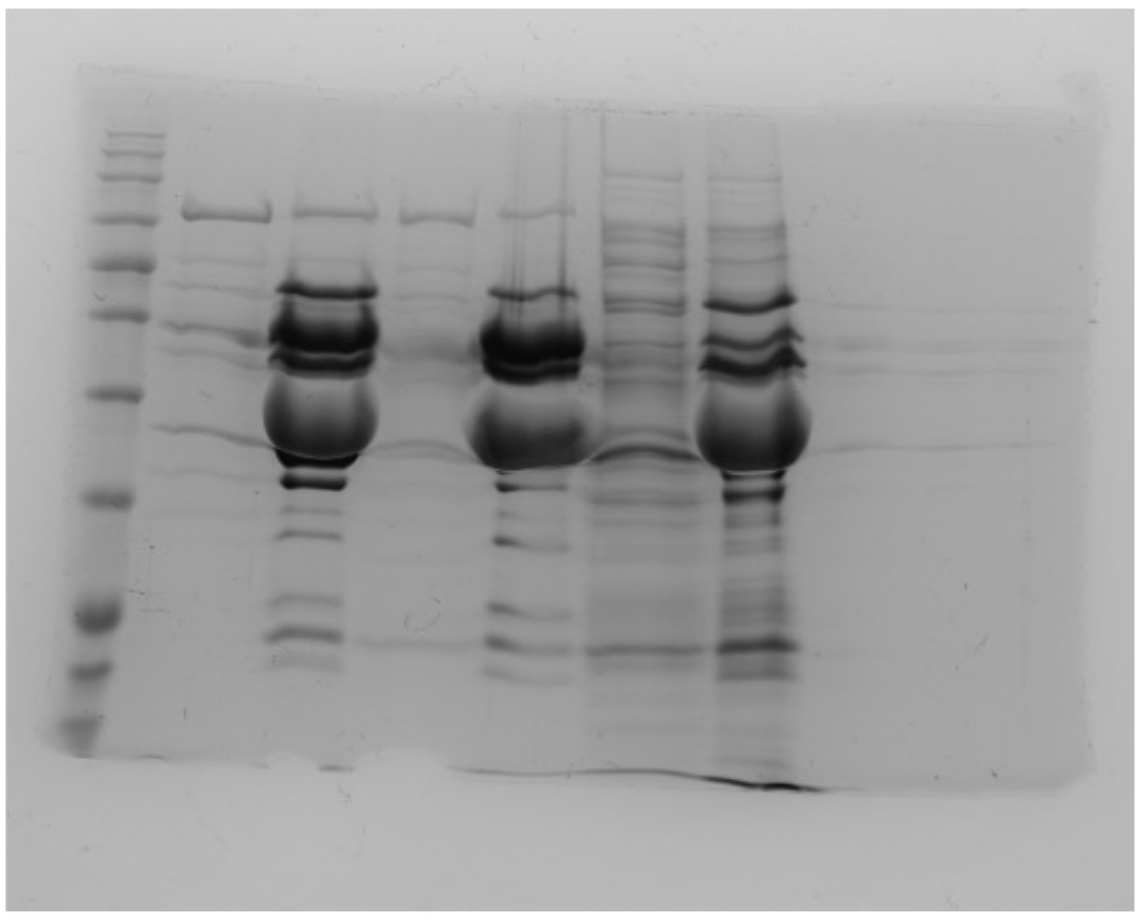

Supplementary Fig. 6a

anti-UFM1

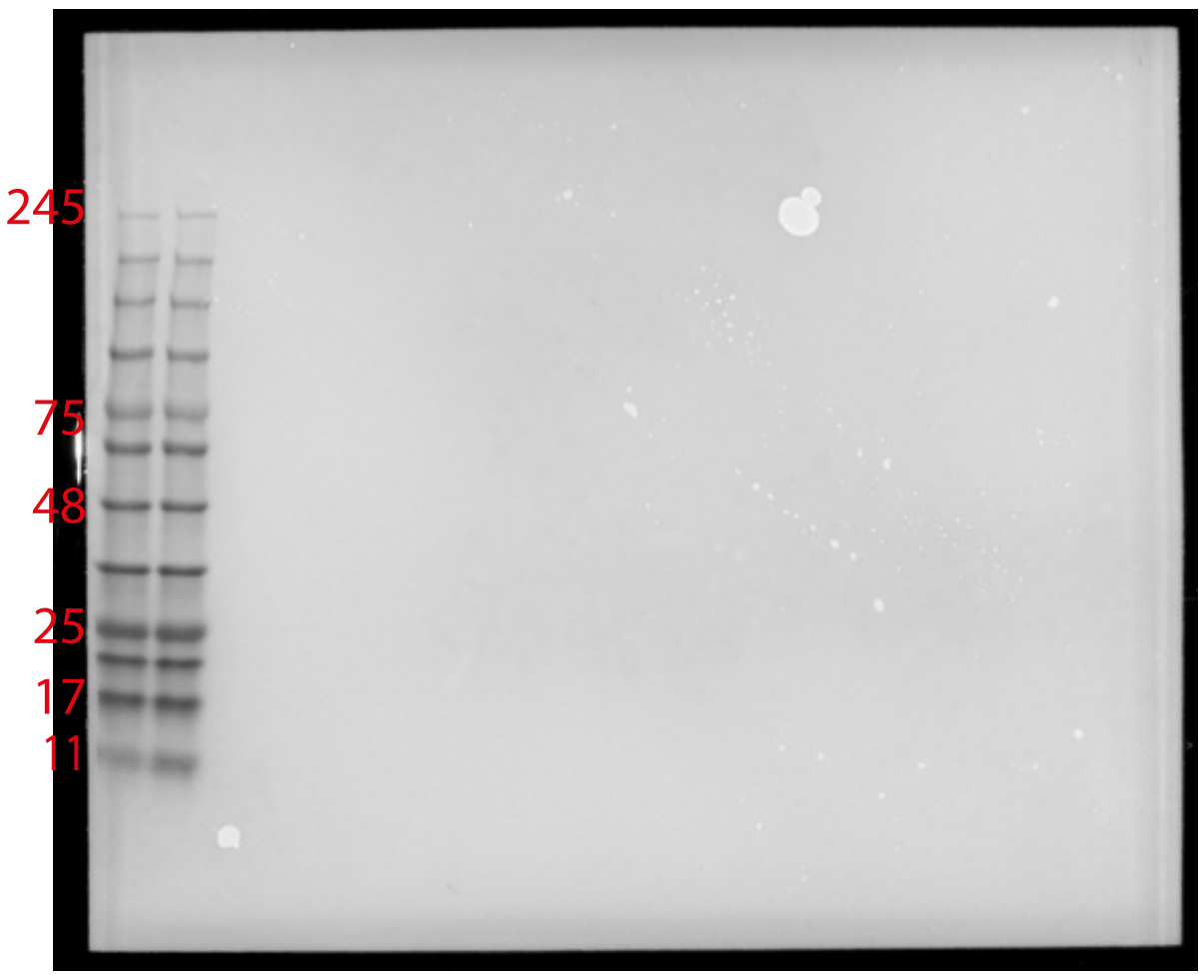

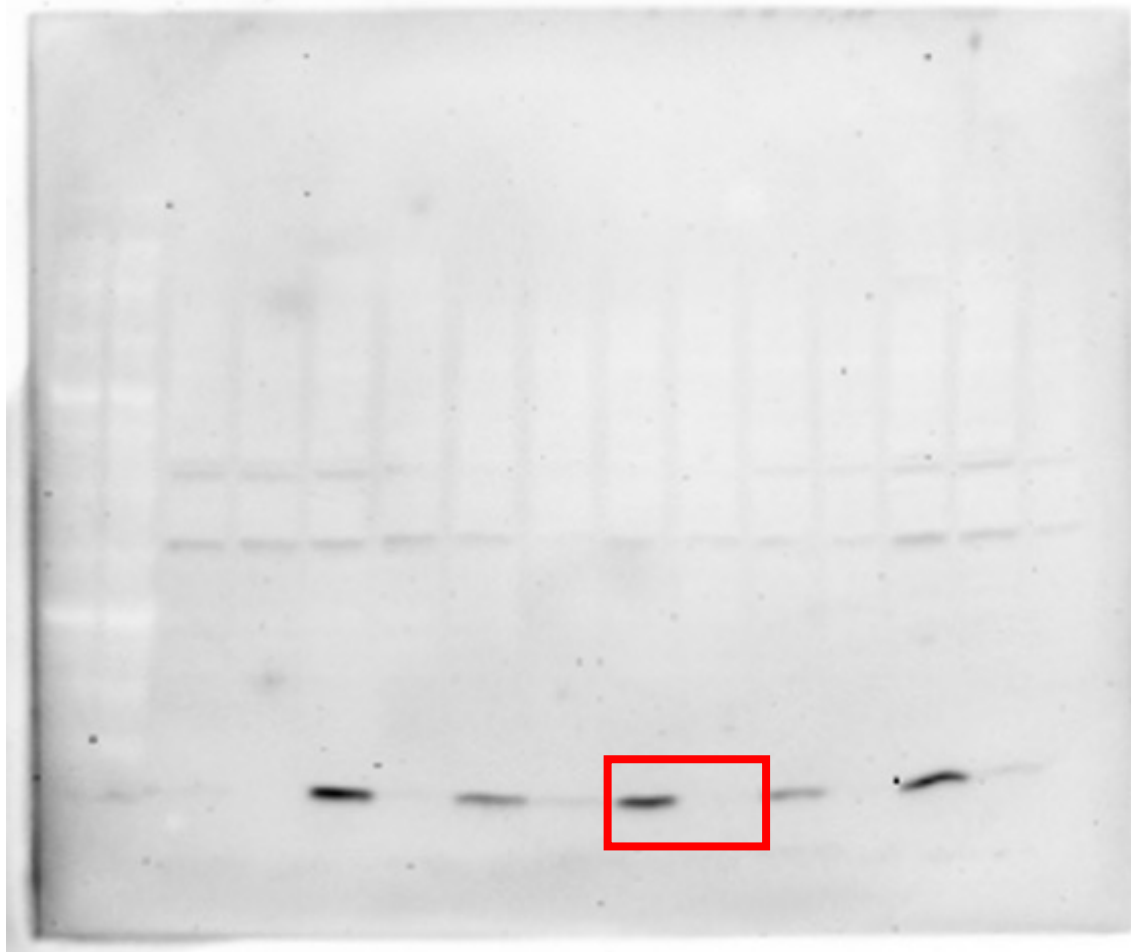

anti-GAPDH

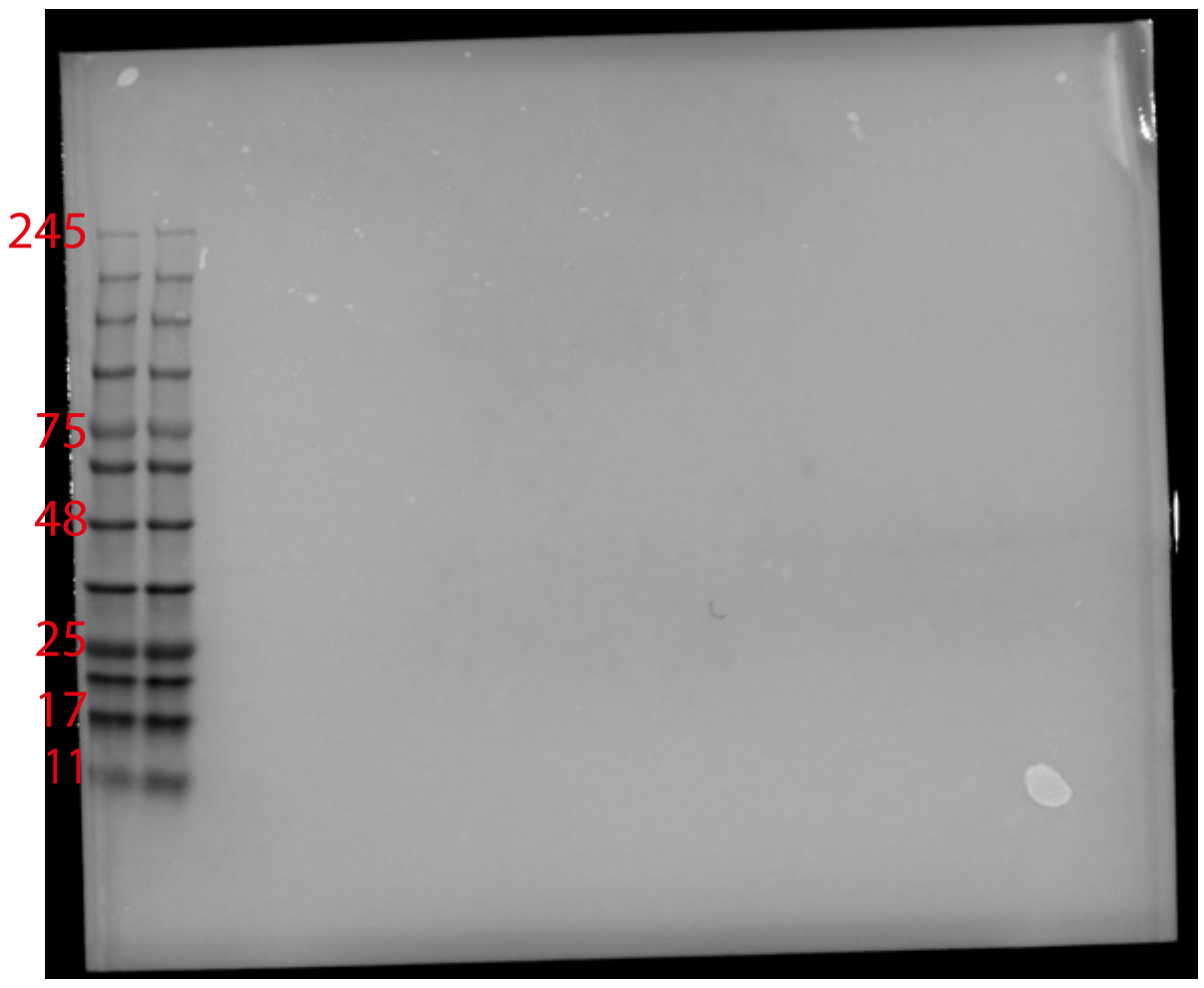

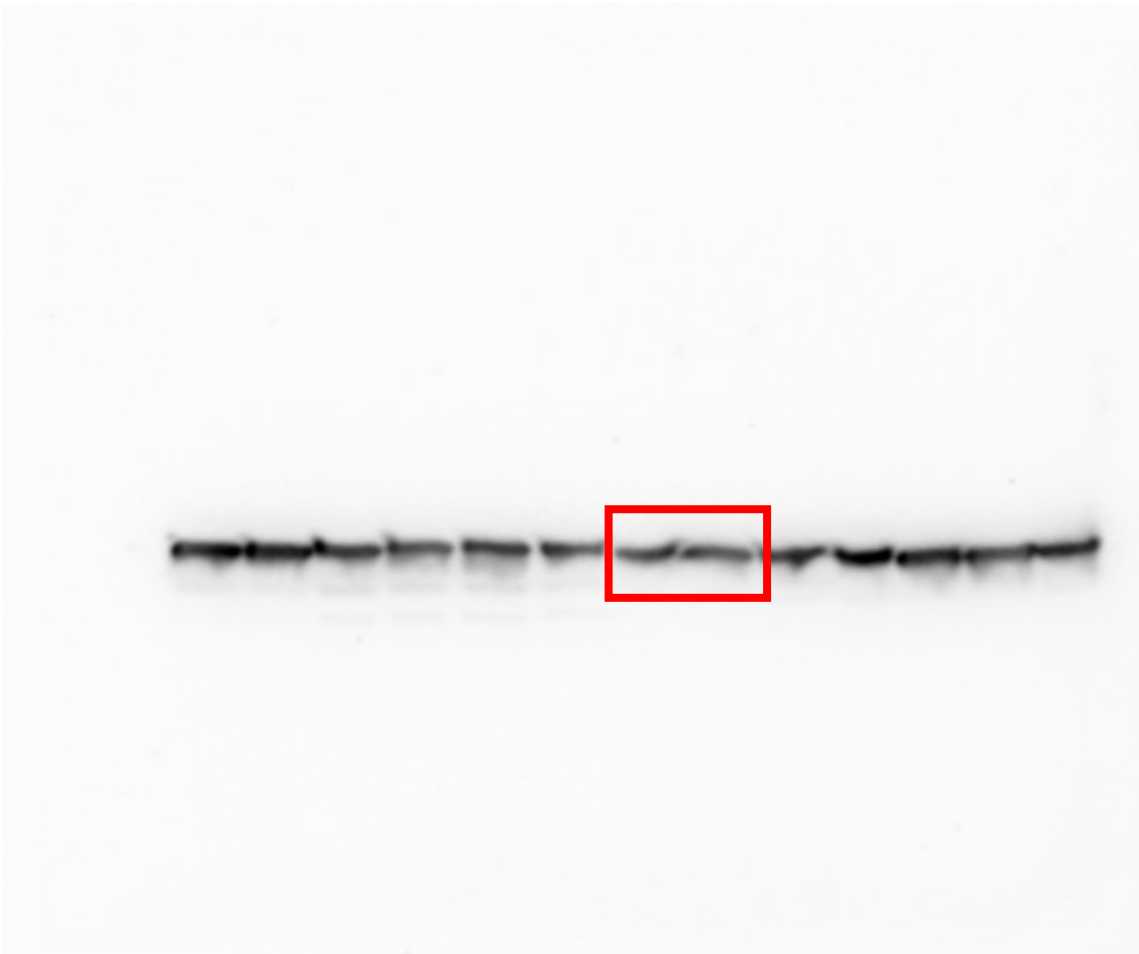

Supplementary Fig. 7a

anti-GBP1

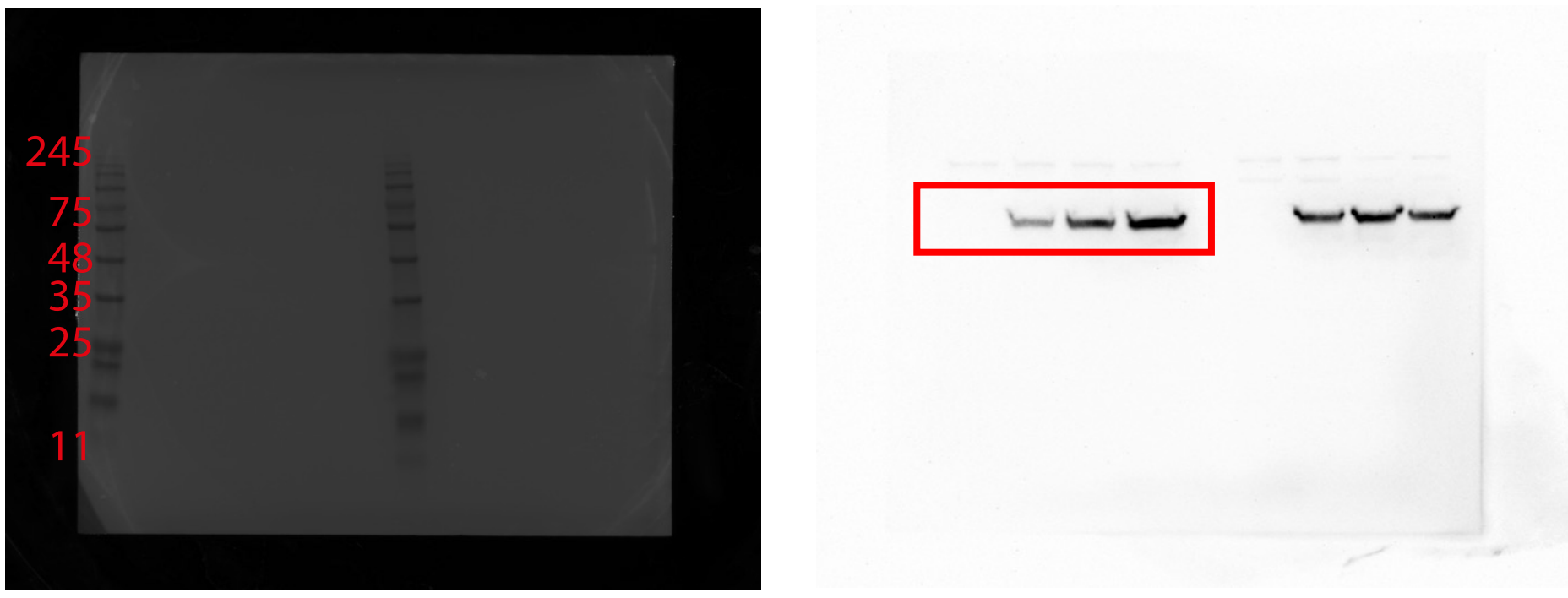

anti-UFL1

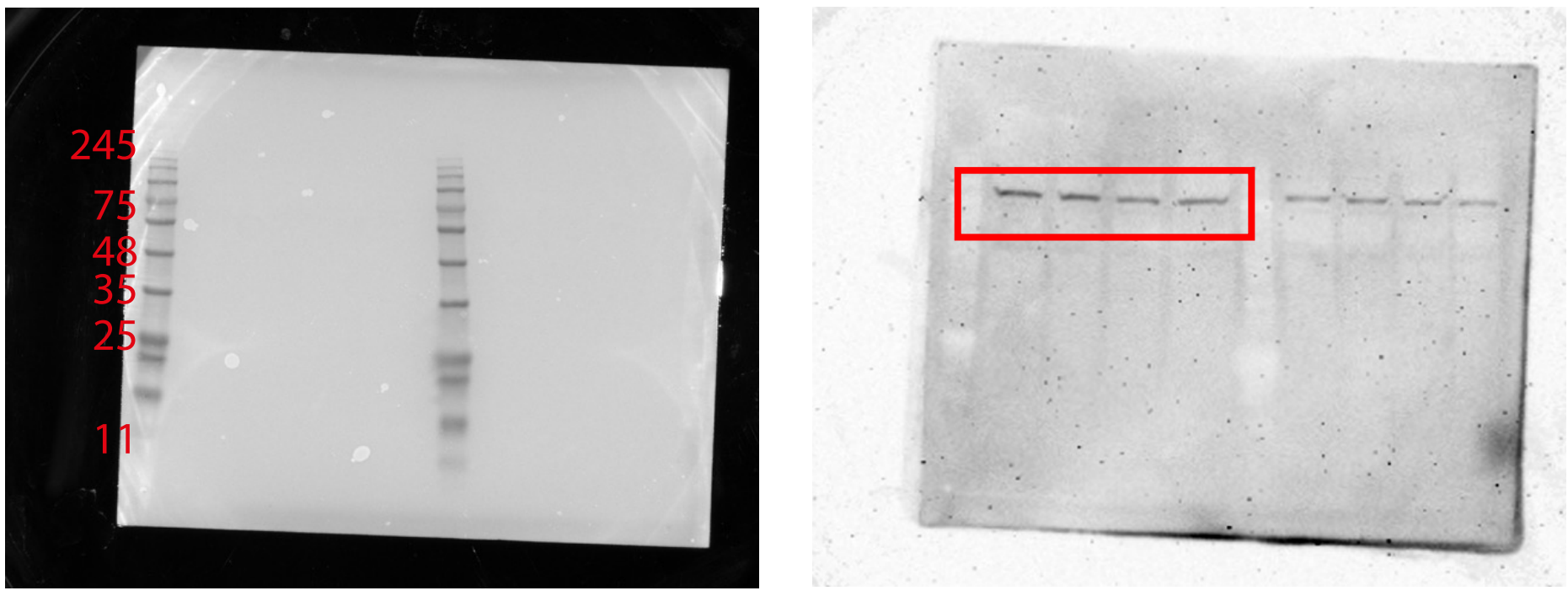

anti-GAPDH

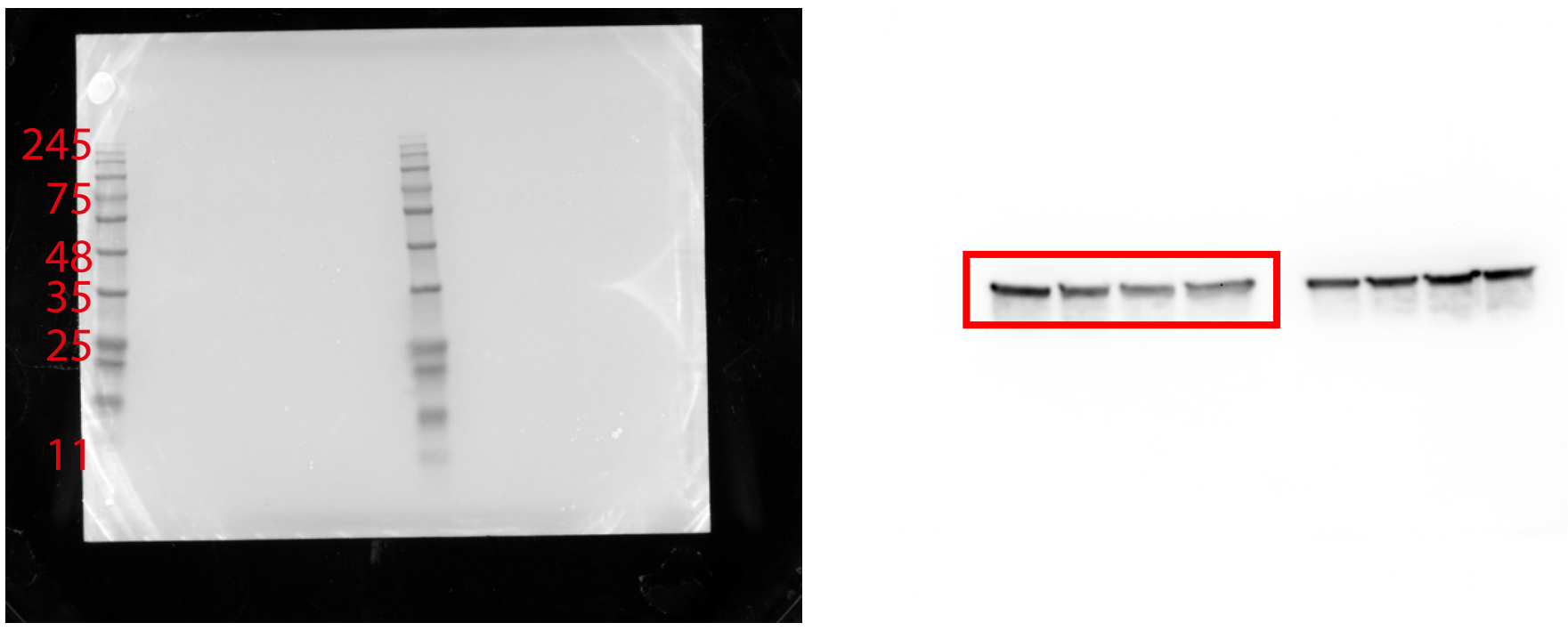

anti-UFM1

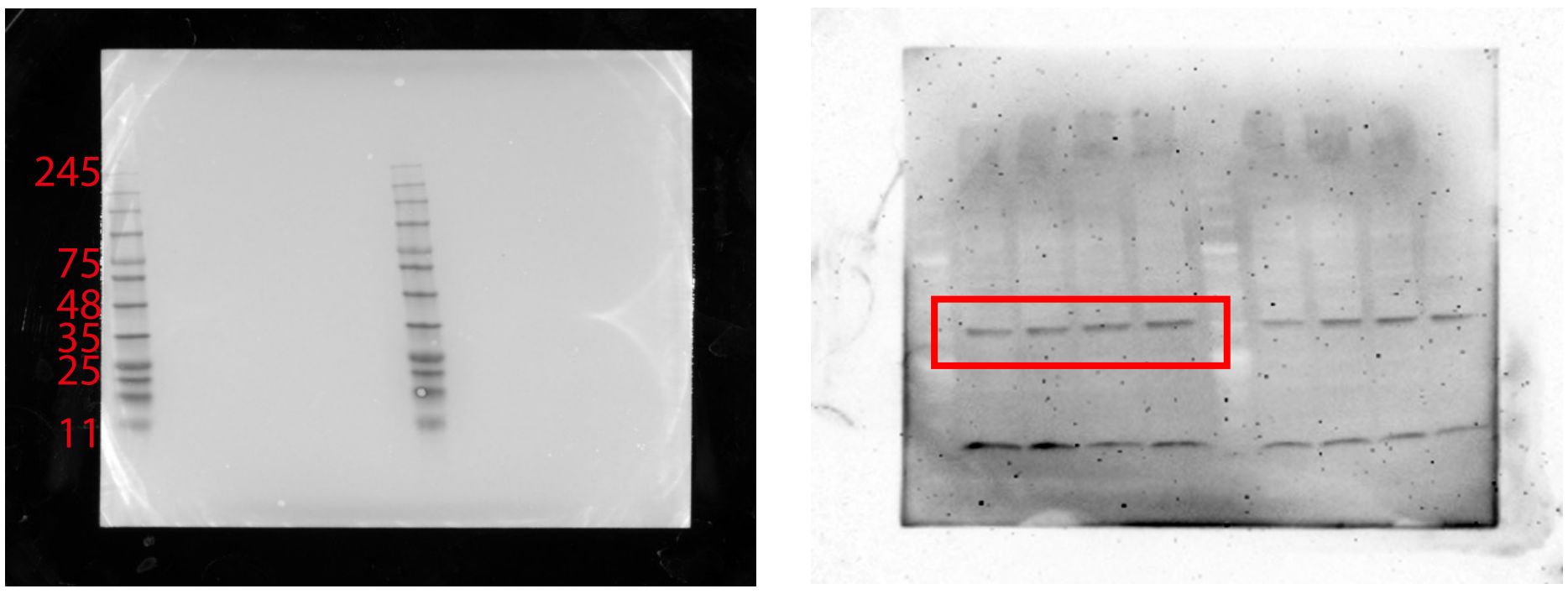

anti-GADPH

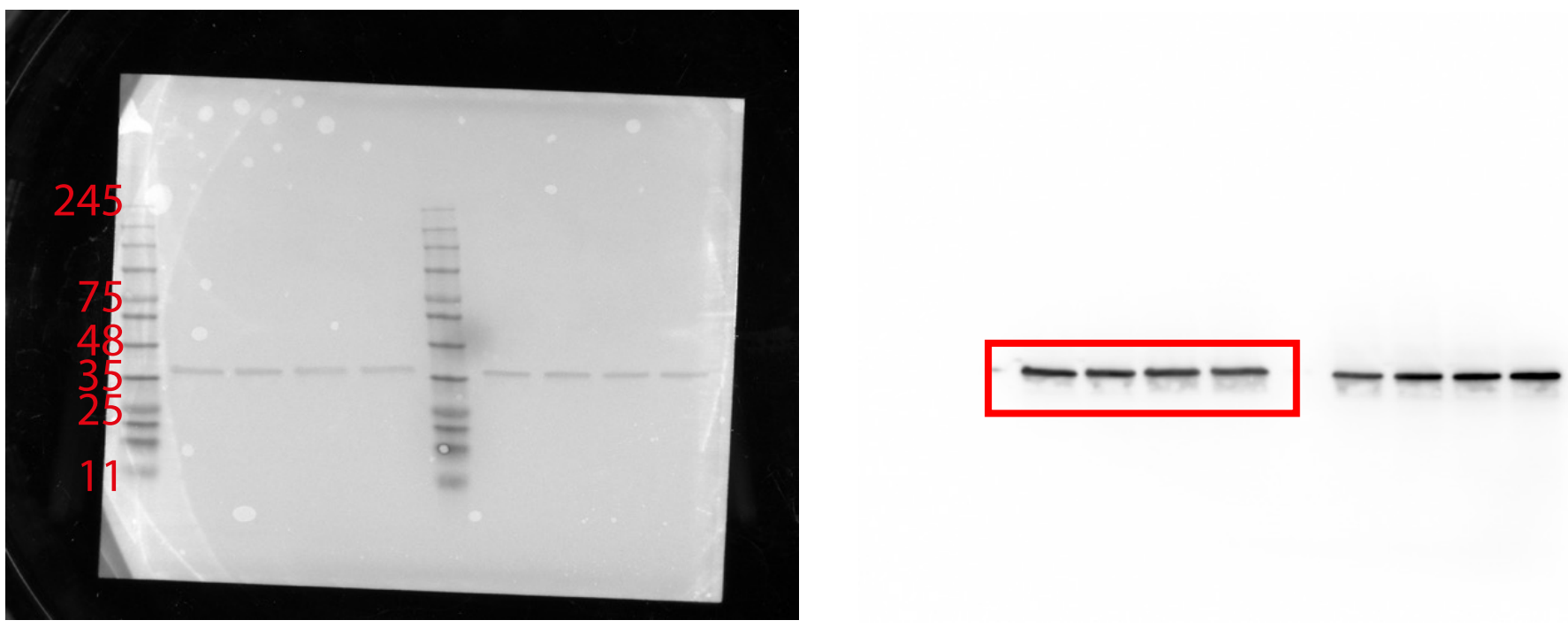

Supplement: Supplementary file 1 [file 41467_2026_77860_MOESM1_ESM.pdf]
