# Supplementary material for: Proximity biotinylation at the host-Shigella interface reveals UFMylation as an antibacterial pathway
Source: Nat Commun. 2026 Sep 17;17:10930. doi: 10.1038/s41467-026-77860-y (PMC13680623; doi:10.1038/s41467-026-77860-y)
Supplement: Supplementary file 2 [file 41467_2026_77860_MOESM2_ESM.pdf]

Description of Additional Supplementary Files.

Supplementary Data File 1. Mass spectrometry protein identification and quantification of *Shigella* WT or Δ*mixE* proximity interactome during infection of HeLa cells. Statistical significance was determined with a moderated t-test implemented in the R package Limma.

Supplementary Data File 2. Proteins identified in the *Shigella* WT or Δ*mixE* proximity interactome that have previously been reported to localise at or near the bacterial surface during infection. Statistical significance was determined with a moderated t-test implemented in the R package Limma.

Supplementary Data File 3. Mass spectrometry identification and quantification of proteins in *Shigella* WT or Δ*rfaC ipaH9.8*::kan samples after recovery from HeLa cells under *UFM1* depletion or control conditions. Statistical significance was calculated with a moderated t-test and multiple testing correction with the Benjamini-Hochberg procedure, both implemented in the R package MsQrob2.

Supplementary Movie 1. Proximity biotinylation in zebrafish larvae. 3D reconstruction of a confocal microscopy z-stack showing a *Tg(lyzC:DsRed2)*^{nz50} zebrafish larva with fluorescently labelled neutrophils infected at the tail musculature with *Shigella* WT functionalised for proximity biotinylation. GFP-APEX2 coated bacteria (green) have been phagocytosed by neutrophils (red). Biotinylated proteins (magenta) appear around phagocytosed bacteria. The 3D grid overlay corresponds to 30 μm intervals.
