# Supplementary material for: Proximity biotinylation at the host-Shigella interface reveals UFMylation as an antibacterial pathway
Source: Nat Commun. 2026 Sep 17;17:10930. doi: 10.1038/s41467-026-77860-y (PMC13680623; doi:10.1038/s41467-026-77860-y)
Supplement: Supplementary file 4 [file 41467_2026_77860_MOESM8_ESM.pdf]

nature portfolio

Peer Review File

Proximity biotinylation at the host-*Shigella* interface reveals UFMylation as an antibacterial pathway

Corresponding Author: Professor Serge Mostowy

This manuscript has been previously reviewed at another journal. This document only contains information relating to versions considered at Nature Communications.

This file contains all reviewer reports in order by version, followed by all author rebuttals in order by version.

Version 0:

Reviewer comments:

Reviewer #1

(Remarks to the Author)
The revised manuscript benefitted from the addition of the Salmonella LAMP1 staining quantification analysis. This new data raises a few questions what would benefit from addressing:
- Previous experiments showed a mean recruitment frequency of UFL1 to Salmonella of ~25% (Fig 2i and 2k), however in the new experiment under comparable conditions, the number decreases to ~10%. What could this mean?
- If this occurrence is at ~10%, would one expect that is this phenomenon important for Salmonella?
- Single marker quantifications were an important addition for the reader. The LAMP1 frequency observed is much lower than one would expect for Salmonella (most bacteria should be LAMP1-positive). Why is this?

(Remarks on code availability)

Open Access This Peer Review File is licensed under a Creative Commons Attribution 4.0 International License, which permits use, sharing, adaptation, distribution and reproduction in any medium or format, as long as you give appropriate credit to the original author(s) and the source, provide a link to the Creative Commons license, and indicate if changes were made.
In cases where reviewers are anonymous, credit should be given to 'Anonymous Referee' and the source.
The images or other third party material in this Peer Review File are included in the article's Creative Commons license, unless indicated otherwise in a credit line to the material. If material is not included in the article's Creative Commons license and your intended use is not permitted by statutory regulation or exceeds the permitted use, you will need to obtain permission directly from the copyright holder.
To view a copy of this license, visit https://creativecommons.org/licenses/by/4.0/

Reviewers' comments:

Reviewer #1 (Remarks to the Author):

The revised manuscript benefitted from the addition of the Salmonella LAMP1 staining quantification analysis.

These additional data were in agreement with our previous review experiments using cytosolic reporters (*Salmonella*) and digitonin permeabilization assays (*Shigella*), and further confirm our manuscript message that UFMylation targets cytosolic bacteria.

This new data raises a few questions what would benefit from addressing:
- Previous experiments showed a mean recruitment frequency of UFL1 to Salmonella of ~25% (Fig 2i and 2k), however in the new experiment under comparable conditions, the number decreases to ~10%. What could this mean?

A key methodological difference between the two sets of experiments is how intracellular bacteria were identified for quantification. In quantifications from the original submission (Fig. 2i, k), *S.* Typhimurium was identified using DAPI, which captures the intracellular bacterial population in full. In quantifications from the revised submission (Fig. 2o), we used GFP-expressing *S.* Typhimurium, with bacterial identification based on the presence of fluorescent reporter. Given our observations with *S. flexneri* that UFL1 recruitment is associated with loss of fluorescent reporter (Fig. 5a, b), it is highly likely that any difference in bacterial identification (DAPI vs fluorescent reporter) accounts for the lower recruitment frequency observed with GFP-based quantification.

- If this occurrence is at ~10%, would one expect that is this phenomenon important for Salmonella?

Our data show that UFL1 recruitment to *S.* Typhimurium is important because genetic and/or pharmacological inhibition of the UFMylation pathway results in significantly increased bacterial burden in both epithial cells and zebrafish larvae, where we also observed significantly increased mortality upon infection. These results highlight UFMylation as an important component of cell-autonomous immunity alternative to ubiquitin-mediated autophagy.

Most of our experimental work was performed using *S. flexneri*, a cytosolic-dwelling pathogen. Our experiments with *S.* Typhimurium are performed to further support UFL1 targetting cytosolic pathogens and to generalise findings to other bacterial species.

Our values of 10-25% mean recruitment frequency of UFL1 represents an early timepoint for *S.* Typhimurium (3 h.p.i., Fig. 2) to be consistent with our *S. flexneri* experiments (3 h.p.i., Fig. 2). Future work will address in depth *S.* Typhimurium timecourse including later timepoints (and also a wide variety of intracellular pathogens), where we predict a higher number of bacteria would be UFL1-positive.

- Single marker quantifications were an important addition for the reader. The LAMP1 frequency observed is much lower than one would expect for Salmonella (most bacteria should be LAMP1-positive). Why is this?

LAMP1 frequency is well known to vary across differences in precise experimental conditions being tested; in our case we used MOI of 100, infection of Hela cells for 3h, membrane permeabilization using saponin. Importantly, we employed strict identification criteria and considered bacteria to be LAMP1-positive only for bacteria in a fully intact compartment (not just associated to LAMP1).

To support data obtained using LAMP1, we assessed recruitment of UFL1 to cytosolic *S.* Typhimurium using using the cytosolic reporter SINA1.7 (Supplementary Fig. 4k) and by quantifying bacterial numbers per cell (Supplementary Fig. 4l). In all cases we show that UFL1 detects cytosolic *S.* Typhimurium, in agreement with our results for *S. flexneri*.
